# Synthesis and *in vitro* anticancer activity of certain novel 1-(2-methyl-6-arylpyridin-3-yl)-3-phenylureas as apoptosis-inducing agents

**DOI:** 10.1080/14756366.2018.1547286

**Published:** 2019-02-05

**Authors:** Wagdy M. Eldehna, Ghada S. Hassan, Sara T. Al-Rashood, Tarfah Al-Warhi, Ahmed E. Altyar, Hamad M. Alkahtani, Abdulrahman A. Almehizia, Hatem A. Abdel-Aziz

**Affiliations:** a Department of Pharmaceutical Chemistry, Faculty of Pharmacy, Kafrelsheikh University, Kafrelsheikh, Egypt;; b Department of Medicinal Chemistry, Faculty of Pharmacy, Mansoura University, Mansoura, Egypt;; c Department of Pharmaceutical Chemistry, College of Pharmacy, King Saud University, Riyadh, Saudi Arabia;; d Department of Chemistry, College of Science, Princess Nourah bint Abdulrahman University, Riyadh, Saudi Arabia;; e Department of Clinical Pharmacy, Faculty of Pharmacy, King Abdulaziz University, Jeddah, Saudi Arabia;; f Department of Applied Organic Chemistry, National Research Center, Cairo, Egypt

**Keywords:** Anticancer agents, apoptosis, cell cycle, pyridine-urea, synthesis

## Abstract

In connection with our research program on the development of novel anticancer candidates, herein we report the design and synthesis of novel series of 1-(2-methyl-6-arylpyridin-3-yl)-3-phenylureas **5a–l**. The target pyridins were evaluated for their *in vitro* anticancer activity against two cancer cell lines: non-small cell lung cancer A549 cell line and colon cancer HCT-116 cell line. Compound **5l** emerged as the most active congener towards both A549 and HCT-116 cell lines with IC_50_ values equal to 3.22 ± 0.2 and 2.71 ± 0.16 µM, respectively, which are comparable to those of Doxorubicin; 2.93 ± 0.28 and 3.10 ± 0.22, respectively. Furthermore, compound **5l** stood out as the most potent pyridine derivative (mean % GI = 40), at US-NCI Developmental Therapeutic Program anticancer assay, with broad-spectrum antitumor activity against the most tested cancer cell lines from all subpanels. Compound **5l** was able to provoke apoptosis in HCT-116 cells as evidenced by the decreased expression of the anti-apoptotic Bcl-2 protein, and the enhanced expression of the pro-apoptotic proteins levels; Bax, cytochrome C, p53, caspase-3 and caspase-9. Moreover, **5l** disrupted the HCT-116 cell cycle via alteration of the Sub-G_1_ phase and arresting the G_2_-M stage. Also, **5l** showed a significant increase in the percent of annexinV-FITC positive apoptotic cells from 1.99 to 15.76%.

## Introduction

Apoptosis, a self-automated cell death, represents the principal pathway in tissue homeostasis and in animal development; in addition, it is the main pathway for the clearance of aged or defective cells in the body. Mainly, two major signaling pathways for apoptotic cell death have been signified. The first one is the extrinsic cytoplasmic pathway that is triggered via pro-apoptotic ligands binding to the cell surface death receptor. Whereas, the second is the intrinsic mitochondrial apoptotic pathway that results from an intracellular cascade of events that are mainly produced by cellular stress, in which mitochondrial permeabilization plays a crucial role. Both extrinsic and intrinsic pathways converge onto the activation of effector caspases, resulting in apoptotic cell death program. During cancer pathogenesis, apoptosis deregulation has been widely recognized as a hallmark of cancer. Accordingly, induction of apoptosis in tumor cells has stood out as a successful tactic for combating different human malignancies, in the current medical era^1–3^.

**Figure 1:**
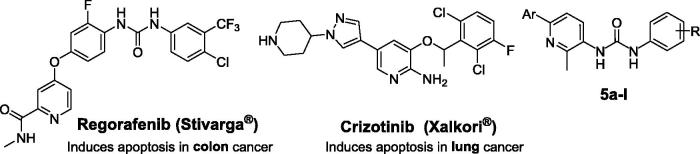
Structures of certain pyridine-based approved anticancer drugs, and the target pyridines **5a–l**.

On the other hand, non-fused pyridines have stood out as a promising class of anticancer agents with efficient pro-apoptotic activity. Regorafenib (Stivarga®, Figure 1), a pyridine-based biphenyl urea derivative developed by Bayer^4^, inhibits angiogenickinases VEGFR-1/3, FGFR1, PDGFRb, and Tie-2. Regorafenib was approved by FDA, in September 2012, for the treatment of metastatic colorectal cancer (mCRC)^5^. The anticancer effect of Regorafenib is thought to be mediated by apoptosis induction, in addition to its anti-angiogenic and anti-proliferative effects^6^^,^^7^. Crizotinib (Xalkori^®^, Figure 1) is an orally active inhibitor of multiple receptor tyrosine kinases, including anaplastic lymphoma kinase (ALK), Hepatocyte Growth Factor Receptor (HGFR, c-Met), and Recepteur d’Origine Nantais (RON)^8^. Crizotinib was approved for the treatment of adults with previously treated, ALK-positive, advanced non-small cell lung cancer (NSCLC)^9^. Crizotinib likely exerts its anticancer activity via multiple distinct mechanisms such as apoptosis^10^.

Recently, our research group has explored the anticancer activity for novel series of 1–(2-methyl-6-(4-methoxy/3,4-dimethoxyphenyl)-pyridin-3-yl)-3-phenylureas^11^. All these derivatives were evaluated for their growth inhibitory activity against the proliferation of breast cancer cell line (MCF-7), where they displayed promising anti-proliferative activity. On the other hand, examination of their potential anti-angiogenic activity towards vascular endothelial growth factor receptor 2 (VEGFR-2) tyrosine kinase unveiled their incompetence to inhibit VEGFR-2 significantly^11^.

Based on the aforementioned findings and as a part of our ongoing quest towards developing potent anticancer agents^12–20^, herein we report the synthesis and biological evaluation of novel series of 1-(2-methyl-6-arylpyridin-3-yl)-3-phenylureas **5a–l**. Ten selected pyridines **5a**, **5c**–**j** and **5l** were chosen to be *in vitro* evaluated for their antitumor activity at one dose (concentration 10^−5 ^M) primary anticancer assay towards a panel including 85 cancer lines according to US-NCI protocol. In addition, all pyridines **5a–l** were examined for their potential anti-proliferative activity against non-small cell lung cancer A549 cell line and colon cancer HCT-116 cell line. Furthermore, apoptosis induction potential of the target pyridines was examined in HCT-116 cells, in order to acquire more mechanistic insights and to verify and enlighten the antitumor properties of the investigated pyridines.

## Materials and methods

### Chemistry

Melting points were measured with a Stuart melting point apparatus and were uncorrected. Infrared (IR) Spectra were recorded as KBr disks using Schimadzu FT-IR 8400S spectrophotometer. ^1^H-NMR and ^13^C-NMR experiments were carried out using Bruker NMR spectrometer (400/100 MHz). Chemical shifts (*δ*_H_) are reported relative to TMS as the internal standard. All coupling constant (*J*) values are given in hertz. Chemical shifts (*δ*_C_) were reported as follows: s, singlet; d, doublet; m, multiplet. High-resolution mass spectra were recorded using a Bruker MicroTOF spectrometer (Bruker Daltonics, Bremen, Germany). All reagents and solvents were dried and purified by the standard techniques. Compounds 2-methyl-6-arylnicotinohydrazides **2a–c**^21–23^ were previously prepared.

### General procedures for preparation of the target pyridines *5a–l*

A solution of hydrazides **2a–c** (10 mmol) and sodium nitrite (1 g, 14 mmol) in hydrochloric acid was stirred for 1 h in an ice bath, then stirring was continued for an additional 1 h at room temperature. The reaction mixture was poured over crushed ice. The precipitated solid was filtered off and air-dried to yield 2-methyl-6-arylnicotinoyl azides **3a**–**c**, which were used in the next step without further purification. Azides **3a**–**c** were heated in refluxing dry xylene for 1 h, then the appropriate aniline derivative was added to this xylene solution. The reaction mixture was heated under reflux temperature for 4 h. After cooling to room temperature, the formed precipitate was filtered, washed with ether and recrystallized from ethanol to afford the target pyridines **5a**–**l**.

### 1-(6-(4-Fluorophenyl)-2-methylpyridin-3-yl)-3-(3-(trifluoromethyl)phenyl)urea *(5a)*

White crystals (yield 70%), m.p. 223–225 °C; IR (KBr, *ν* cm^−1^) 3393 (NH), 1731 (C=O); ^1^H NMR (CDCl_3_-d) *δ* ppm: 2.64 (s, 3H, CH_3_), 6.30 (s, 1H, NH, D_2_O exchangeable), 6.61 (s, 1H, NH, D_2_O exchangeable), 7.15 (t, 2H, *J* = 8.8 Hz, Ar-H), 7.38 (d, 1H, *J* = 8.4 Hz, Ar-H), 7.46 (t, 1H, *J* = 8.0 Hz, Ar-H), 7.60–7.65 (m, 2H, Ar-H), 7.71 (s, 1H, Ar-H), 7.98 (dd, 2H, *J* = 8.8 Hz, *J* = 5.6 Hz, Ar-H), 8.06 (d, 1H, *J* = 8.4 Hz, Ar-H);^13^C NMR (DMSO-d_6_) *δ* ppm: 21.34 (CH_3_), 115.36, 115.53, 117.69, 121.75, 128.04, 128.67, 130.06, 132.47, 135.00, 140.42, 148.02, 148.45, 152.62 (CO), 161.43, 163.38 (=C-F); HRMS (ESI) *m/z* calcd for [M + H]^+^ (C_20_H_16_N_3_OF_4_): 390.12240, found: 390.12286.

### 1-(3,5-Bis(trifluoromethyl)phenyl)-3-(6-(4-fluorophenyl)-2-methylpyridin-3-yl)urea* (5b)*

White crystals (yield 65%), m.p. 235–237 °C; IR (KBr, *ν* cm^−1^) 3390 (NH), 1733 (C=O); ^1^H NMR (CDCl_3_-d) *δ* ppm: 2.58 (s, 3H, CH_3_), 6.31 (s, 1H, NH, D_2_O exchangeable), 6.59 (s, 1H, NH, D_2_O exchangeable), 7.17 (t, 2H, *J* = 8.8 Hz, Ar-H), 7.59 (s, 1H, Ar-H), 7.63 (d, 1H, *J* = 8.4 Hz, Ar-H), 7.89 (s, 2H, Ar-H), 8.02–8.10 (m, 3H, Ar-H); ^13 ^C NMR (DMSO-d_6_) *δ* ppm: 21.58 (CH_3_), 115.44, 115.61, 117.75, 128.09, 128.15, 128.66, 132.72, 135.10, 147.79, 148.34, 152.94 (C=O), 161.49, 163.44 (=C–F).

### Ethyl 4-(3-(6-(4-fluorophenyl)-2-methylpyridin-3-yl)ureido)benzoate* (5c)*

White crystals (yield 73%), m.p. 209–211 °C; IR (KBr, *ν* cm^−1^) 3389 (NH), 1733 (C=O); ^1^H NMR (CDCl_3_-d) *δ* ppm: 1.39 (t, 3H*, J* = 7.2 Hz, –OCH_2_CH_3_), 2.62 (s, 3H, –CH_3_), 4.37 (q, 2H, *J* = 7.2 Hz, –OCH_2_CH_3_), 6.36 (s, 1H, NH, D_2_O exchangeable), 6.72 (s, 1H, NH, D_2_O exchangeable), 7.14 (t, 2H, *J* = 8.8 Hz, Ar-H), 7.49 (d, 2H, *J* = 8.4 Hz, Ar-H), 7.60 (d, 1H, *J* = 8.4 Hz, Ar-H), 7.98–8.10 (m, 5H, Ar-H); ^13 ^C NMR (DMSO-d_6_) *δ* ppm: 14.30 (CH_3_), 21.37 (CH_3_), 60.39 (CH_2_), 115.41, 115.58, 117.34, 117.75, 122.98, 128.08, 128.50, 130.51, 132.50, 135.04, 144.16, 147.93, 148.47, 152.35 (C=O), 161.48, 163.43 (=C–F), 165.48 (–COO–) HRMS (ESI) *m/z* calcd for [M + H]^+^ (C_22_H_21_N_3_O_3_F): 394.15615, found: 394.15628.

### 1-(Benzo[d][1, 3]dioxol-5-yl)-3-(6-(4-fluorophenyl)-2-methylpyridin-3-yl)urea *(5d)*

White crystals (yield 62%), m.p. 254–256 °C; IR (KBr, *ν* cm^−1^) 3394 (NH), 1733 (C=O); ^1^H NMR (CDCl_3_-d) *δ* ppm: 2.48 (s, 3H, CH_3_), 6.04 (s, 2H, CH_2_), 6.23 (s, 1H, NH, D_2_O exchangeable), 6.34 (s, 1H, NH, D_2_O exchangeable), 6.84 (d, 1H, *J* = 8.0 Hz, Ar-H), 6.97–7.02 (m, 2H, Ar-H), 7.12 (t, 2H, *J* = 8.4 Hz, Ar-H), 7.54–7.57 (m, 1H, Ar-H), 7.94–7.98 (m, 2H, Ar-H), 8.19 (d, 1H, *J* = 8.0 Hz, Ar-H); ^13^C NMR (DMSO-d_6_) *δ* ppm: 21.36 (CH_3_), 100.82 (O–CH_2_–O), 108.20, 110.93, 115.33, 115.50, 117.65, 127.99, 132.94, 133.89, 135.08, 142.16, 147.27, 147.83, 152.66 (C=O), 161.35, 163.30 (=C–F); HRMS (ESI) *m/z* calcd for [M + H]^+^ (C_20_H_17_N_3_O_3_F): 366.12485, found: 366.12405.

### 1-(6-(4-Chlorophenyl)-2-methylpyridin-3-yl)-3-(3-(trifluoromethyl)phenyl)urea *(5e)*

White crystals (yield 68%), m.p. 241-242 °C; IR (KBr, *ν* cm^−1^) 3378 (NH), 1733 (C=O); ^1^H NMR (CDCl_3_-d) *δ* ppm: 2.48 (s, 3H, CH_3_), 6.25 (s, 1H, NH, D_2_O exchangeable), 6.36 (s, 1H, NH, D_2_O exchangeable), 7.38 (d, 1H, *J* = 8.4 Hz, Ar-H), 7.41 (d, 2H, *J* = 8.8 Hz, Ar-H), 7.52-7.58 (m, 3H, Ar-H), 7.78 (s, 1H, Ar-H), 7.91 (d, 2H, *J* = 8.4 Hz, Ar-H), 8.24 (d, 1H, *J* = 8.4 Hz, Ar-H); ^13 ^C NMR (DMSO-d_6_) *δ* ppm: 21.57 (CH_3_), 1117.91, 127.69, 128.41, 128.68, 133.05, 137.32, 147.75, 147.84, 152.82 (C=O); HRMS (ESI) *m/z* calcd for [M-H]^+^ (C_20_H_14_N_3_OClF_3_): 404.07830, found: 404.07779.

### 1-(6-(4-Chlorophenyl)-2-methylpyridin-3-yl)-3-(4-methoxyphenyl) urea *(5f)*

White crystals (yield 55%), m.p. 264-265 °C; IR (KBr, *ν* cm^−1^) 3392 (NH), 1733 (C=O); ^1^H NMR (CDCl_3_-d) *δ* ppm: 2.41 (s, 3H, CH_3_), 3.86 (s, 3H, –OCH_3_), 6.27 (s, 1H, NH, D_2_O exchangeable), 6.33 (s, 1H, NH, D_2_O exchangeable), 6.97 (d, 2H, *J* = 8.4 Hz, Ar-H), 7.31 (d, 2H, *J* = 8.8 Hz, Ar-H), 7.41 (d, 2H, *J* = 8.8 Hz, Ar-H), 7.57 (d, 1H, *J* = 8.0 Hz, Ar-H), 7.91 (d, 2H, *J* = 8.4 Hz, Ar-H), 8.26 (d, 1H, *J* = 8.4 Hz, Ar-H); ^13 ^C NMR (DMSO-d_6_) *δ* ppm: 21.37 (CH_3_), 55.18 (OCH_3_), 114.08, 117.88, 119.92, 127.45, 127.57, 127.66, 128.61, 132.49, 132.87, 133.47, 137.30, 137.39, 147.13, 147.23, 147.69, 147.79, 152.67 (C=O), 154.58 (=C–OCH_3_); HRMS (ESI) *m/z* calcd for [M − H]^+^ (C_20_H_17_N_3_O_2_Cl): 366.10148, found: 366.10152.

### 1-(Benzo[d][1,3]dioxol-5-yl)-3-(6-(4-chlorophenyl)-2-methylpyridin-3-yl)urea *(5g)*

White crystals (yield 63%), m.p. 271–273 °C; IR (KBr, *ν* cm^−1^) 3388 (NH), 1733 (C=O); ^1^H NMR (CDCl_3_-d) *δ* ppm: 2.47 (s, 3H, CH_3_), 6.04 (s, 2H, –OCH_2_O–), 6.28 (s, 1H, NH, D_2_O exchangeable), 6.38 (s, 1H, NH, D_2_O exchangeable), 6.79–6.87 (m, 2H, Ar-H), 6.96 (d, 1H, *J* = 2.1 Hz, Ar-H), 7.42 (d, 2H, *J* = 8.4 Hz, Ar-H), 7.57 (d, 1H, *J* = 8.4 Hz, Ar-H), 7.91 (d, 2H, *J* = 8.8 Hz, Ar-H), 8.22 (d, 1H, *J* = 8.4 Hz, Ar-H); ^13 ^C NMR (DMSO-d_6_) *δ* ppm: 21.36 (CH_3_), 100.84 (O–CH_2_–O), 108.21, 110.96, 117.88, 127.59, 128.62, 132.90, 133.32, 133.83, 137.36, 142.20, 147.28, 152.60 (C=O); HRMS (ESI) *m/z* calcd for [M-H]^+^ (C_20_H_15_N_3_O_3_Cl): 380.08074, found: 380.08115.

### 1-(4-Fluorophenyl)-3-(2-methyl-6-(thiophen-2-yl)pyridin-3-yl)urea *(5h)*

White crystals (yield 60%), m.p. 217–219 °C; IR (KBr, *ν* cm^−1^) 3393 (NH), 1733 (C=O); ^1^H NMR (CDCl_3_-d) *δ* ppm: 2.50 (s, 3H, CH_3_), 6.20 (s, 1H, NH, D_2_O exchangeable), 6.33 (s, 1H, NH, D_2_O exchangeable), 7.07–7.13 (m, 3H, Ar-H), 7.35–7.39 (m, 3H, Ar-H), 7.54–7.56 (m, 2H, Ar-H), 8.05 (d, 1H, *J* = 8.4 Hz, Ar-H); ^13 ^C NMR (DMSO-d_6_) *δ* ppm: 21.09 (CH_3_), 115.30, 115.47, 116.47, 119.85, 119.91, 123.81, 127.07, 127.11, 128.16, 128.21, 132.55, 135.86, 144.65, 145.36, 147.51, 152.64 (C=O); HRMS (ESI) *m/z* calcd for [M-H]^+^ (C_17_H_13_N_3_OFS): 326.07688, found: 326.07718.

### 1-(4-Chlorophenyl)-3-(2-methyl-6-(thiophen-2-yl) pyridin-3-yl)urea *(5i)*

White crystals (yield 71%), m.p. 234–236 °C; IR (KBr, *ν* cm^−1^) 3398 (NH), 1733 (C=O); ^1^H NMR (CDCl_3_-d) *δ* ppm: 2.53 (s, 3H, CH_3_), 6.18 (s, 1H, NH, D_2_O exchangeable), 6.36 (s, 1H, NH, D_2_O exchangeable), 7.06–7.14 (m, 1H, Ar-H), 7.35-7.40 (m, 5H, Ar-H), 7.52-7.54 (m, 2H, *J* = 6.5 Hz, Ar-H), 7.99 (d, 1H, *J* = 8.4 Hz, Ar-H); ^13 ^C NMR (DMSO-d_6_) *δ* ppm: 21.08 (CH_3_), 116.48, 119.67, 123.88, 125.51, 127.13, 128.30, 128.72, 132.38, 138.53, 144.61, 145.51, 147.66, 152.46 (C=O); HRMS (ESI) *m/z* calcd for [M-H]^+^ (C_17_H_13_N_3_OClS): 342.04733, found: 342.04752.

### Ethyl 4-(3-(2-methyl-6-(thiophen-2-yl) pyridin-3-yl)ureido)benzoate *(5j)*

White crystals (yield 69%), m.p. 203–204 °C; IR (KBr, *ν* cm^−1^) 3393 (NH), 1733 (C=O); ^1^H NMR (CDCl_3_-d) *δ* ppm: 1.39 (t, 3H*, J* = 7.2 Hz, –OCH_2_CH_3_), 2.55 (s, 3H, –CH_3_), 4.35 (q, 2H, *J* = 7.2 Hz, –OCH_2_CH_3_), 6.53 (s, 1H, NH, D_2_O exchangeable), 6.98 (s, 1H, NH, D_2_O exchangeable), 7.10 (t, 1H, *J* = 4.4 Hz, Ar-H), 7.38 (d, 1H, *J* = 5.2 Hz, Ar-H), 7.46 (d, 2H, *J* = 8.4 Hz, Ar-H), 7.52–7.55 (m, 2H, Ar-H), 7.99–8.02 (m, 3H, Ar-H); ^13 ^C NMR (DMSO-d_6_) *δ* ppm: 14.26 (CH_3_), 21.08 (CH_3_), 60.32 (O–CH_2_), 116.49, 117.28, 122.91, 123.97, 127.21, 128.25, 128.50, 130.45, 132.17, 144.12, 144.57, 145.73, 147.86, 152.26 (C=O), 165.40 (–COO–); HRMS (ESI) *m/z* calcd for [M − H]^+^ (C_20_H_18_N_3_O_3_S): 380.10744, found: 380.10764.

### 1-(Benzo[d][1, 3]dioxol-5-yl)-3-(2-methyl-6-(thiophen-2-yl)pyridin-3-yl)urea *(5k)*

White crystals (yield 58%), m.p. 239–241 °C; IR (KBr, *ν* cm^−1^) 3388 (NH), 1733 (C=O); ^1^H NMR (CDCl_3_-d) *δ* ppm: 2.44 (s, 3H, CH_3_), 6.03 (s, 2H, –OCH_2_O–), 6.23 (s, 1H, NH, D_2_O exchangeable), 6.31 (s, 1H, NH, D_2_O exchangeable), 6.77 (dd, 1H, *J* = 2.0 Hz, *J* = 8.0 Hz, Ar-H), 6.83 (d, 1H, *J* = 8.0 Hz, Ar-H), 6.97 (d, 1H, *J* = 2.0 Hz, Ar-H), 7.08 (dd, 1H, *J* = 4.0 Hz, *J* = 5.2 Hz, Ar-H), 7.35 (d, 1H, *J* = 5.0 Hz, Ar-H), 7.53-7.54 (m, 2H, Ar-H), 8.12 (d, 1H, *J* = 8.4 Hz, Ar-H); ^13 ^C NMR (DMSO-d_6_) *δ* ppm: 21.08 (CH_3_), 100.87 (O–CH_2_–O), 108.20, 110.96, 116.47, 123.74, 127.01, 127.92, 128.21, 132.66, 133.86, 142.18, 144.68, 145.20, 147.27, 152.61 (C=O); HRMS (ESI) *m/z* calcd for [M − H]^+^ (C_18_H_14_N_3_O_3_S): 352.07614, found: 352.07642.

### 2-(3-(2-Methyl-6-(thiophen-2-yl)pyridin-3-yl)ureido)benzenesulfonamide *(5l)*

White crystals (yield 60%), m.p. 265–266 °C; IR (KBr, *ν* cm^−1^) 3369, 3207 (NH, NH_2_), 1733 (C=O), 1330, 1157 (SO_2_); ^1^H NMR (DMSO-d_6_, 400 MHz) *δ* ppm: 2.50 (s, 3H, CH_3_), 7.11 (t, 1H, H-4 of 2-thienyl, *J* = 4.0 Hz), 7.18 (t, 1H, H-4 of 2-(H_2_NO_2_S)-C_6_H_4_, *J* = 7.6 Hz), 7.52-7.56 (m, 2H, H-5 of 2-(H_2_NO_2_S)-C_6_H_4_, and H-5 of 2-thienyl), 7.60 (s, 2H, SO_2_NH_2_), 7.67 (d, 1H, H-3 of 2-thienyl, *J* = 4.0 Hz), 7.71 (d, 1H, H-5 pyridine, *J* = 8.4 Hz), 7.82 (d, 1H, H-6 of 2-(H_2_NO_2_S)–C_6_H_4_, *J* = 7.6 Hz), 7.97 (d, 1H, H-3 of 2-(H_2_NO_2_S)-C_6_H_4_, *J* = 8.0 Hz), 8.04 (d, 1H, H-4 pyridine, *J* = 8.4 Hz), 8.73 (s, 1H, 8.21 (s, 1H, NH, D_2_O exchangeable), 9.15 (s, 1H, NH, D_2_O exchangeable).

### Biological evaluation

#### In vitro antitumor activity towards 60 cancer cell lines (NCI, USA)

The antitumor assay was performed according to the protocol of the Drug Evaluation Branch, NCI, Bethesda^24–26^. A 48 h drug exposure protocol was adopted, and sulforhodamine B (SRB) assay^27^ was utilized to assess the cell growth and viability, as reported earlier^17^^,^^28^.

#### In vitro anti-Proliferative activity towards A549 and HCT-116 cell lines

A549 (non-small cell lung cancer cell line) and HCT-116 (human colon cancer cell line), were obtained from American Type Culture Collection (Manassas, VA, USA). The cells were maintained in Dulbecco’s Modified Eagle’s Medium (DMEM) (Sigma-Aldrich, St. Louis, MO), and supplemented with 10% heat-inactivated FBS (Hyclone), 10 μg/mL of insulin (Manufacturer, Sigma, St. Louis, MO, USA), and 1% penicillin-streptomycin. MTT assay^29^ was adopted to assess the *in vitro* antitumor activity of the newly synthesized pyridines **5a–l** according to the reported procedures^30^, using Doxorubicin as a standard treatment. Experimental conditions were tested using three replicates (three wells of the 96-well plate per experimental condition) and all experiments were carried out in triplicates. IC_50_ values were calculated by the use of the equation for Boltzman sigmoidal concentration–response curve using the nonlinear regression fitting models by Graph Pad, Prism version 5 (GraphPad Software Inc., La Jolla, CA).

#### ELISA immunoassay

The levels of the apoptotic markers Bax, cytochrome C, p53, caspase-3 and caspase-9 as well as the anti-apoptotic protein Bcl-2 were evaluated using ELISA colorimetric kits per the manufacturer’s instructions, as reported earlier^31^^,^^32^.

#### Cell cycle analysis

HCT-116 cells were treated with pyridine **5l** at its IC_50_ concentration (IC_50_ = 2.71 μM) for 24 h, then cells were washed with ice-cold phosphate-buffered saline (PBS). The treated cells were collected by centrifugation, fixed in ice-cold 70% (*v*/*v*) ethanol, washed with PBS, re-suspended with 100 μg/mL RNase, stained with 40 μg/mL PI, and analyzed by flow cytometry using FACS Calibur (Becton Dickinson, BD, USA). The cell cycle distributions were calculated using CellQuest software 5.1 (Becton Dickinson)^33^.

#### Annexin V-FITC apoptosis assay

Phosphatidylserine externalization was assayed using Annexin V-FITC/PI apoptosis detection kit (BD Biosciences, USA) according to the manufacturer’s instructions, as reported earlier^33^^,^^34^.

## Results and discussion

### Chemistry

**Scheme 1:**
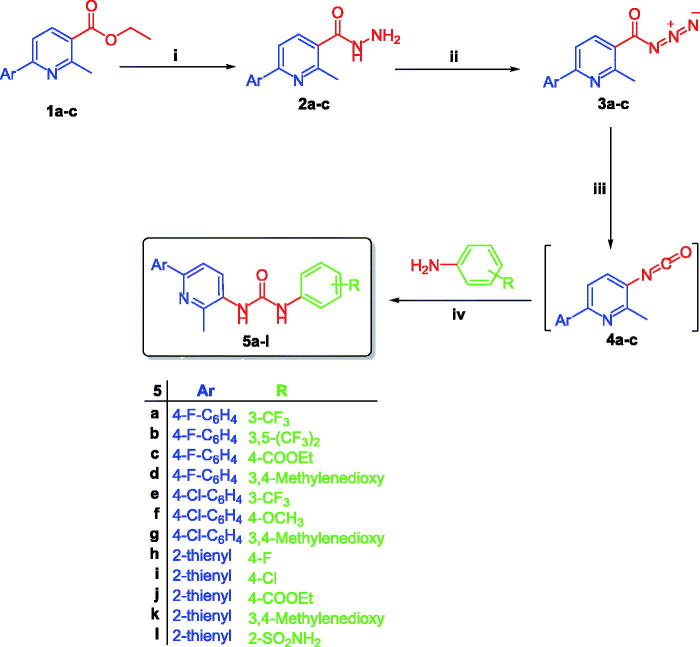
Synthesis of target derivatives **5a**–**l**; (**i**) Ethyl alcohol, NH_2_NH_2_·H_2_O, reflux 3 h.; (**ii**) NaNO_2_, HCl, stirring 2 h.; (**iii**) Xylene, reflux 1 h.; (**iv**) Xylene, reflux 4 h.

The method adopted for preparation of the target pyridines **5a**–**l** is depicted in Scheme 1. Firstly, esters **1a**–**c** were hydrazinolyzed via reaction with hydrazine hydrate in methanol under reflux temperature to furnish 2-methyl-6-arylnicotinohydrazides **2a**–**c** in 75, 71 and 80% yields, respectively. Treatment of hydrazides **2a**–**c** with sodium nitrite in cold hydrochloric acid afforded 2-methyl-6-arylnicotinoyl azides **3a**–**c**, which subsequently subjected to Curtius rearrangement upon heating in xylene to give the corresponding isocyanates derivatives **4a**–**c**. The target hybrids **5a–l** was obtained by reaction of isocyanates derivatives **4a**–**c** with the appropriate aniline derivative in xylene with 55–73% yield (Scheme 1).

The structures of the newly prepared pyridines **5a**–**l** were confirmed under the basis of spectral and elemental analyses which were in full agreement with the postulated structures (Supplementary Material).

### Biological evaluation

#### In vitro antitumor activity towards 60 cancer cell lines (NCI, USA)

The structures of all the newly synthesized pyridines **5a**–**l** were submitted to the National Cancer Institute (NCI) Developmental Therapeutic Program (www.dtp.nci.nih.gov). Ten pyridines **5a**, **5c**–**j** and **5l** were chosen to be *in vitro* evaluated for their antitumor activity. The selected pyridines **5a**, **5c**–**j** and **5l** were examined at one dose (concentration 10^−5 ^M) primary anticancer assay towards a panel including 85 cancer lines. Nine different types of cancer were tested in this assay: colon, ovarian, prostate, leukemia, melanoma, CNS, renal, breast and lung cancers. A 48 h drug exposure protocol was adopted, and sulforhodamine B (SRB) assay^27^ was utilized to assess the cell growth and viability. The results were reported as mean-graph of the percentage growth of the treated cells, and displayed as percentage growth inhibition (GI%) caused by the test pyridines (Tables 1 and 2). Investigation of data in Tables 1 and 2 revealed that the examined pyridines exhibited distinctive patterns of sensitivity and selectivity against the different NCI cancer cell panels.

**Table 1.** Percentage growth inhibition (GI%) of *in vitro* subpanel tumor cell lines at 10 μM concentration for pyridines **5a** and **5c**–**f**.

Subpanel/Cell Line		Compound^a^				
		**5a**	**5c**	**5d**	**5e**	**5f**
Leukemia	CCRF-CEM	25	–	–	22	18
	HL-60(TB)	11	13	–	21	12
	K-562	50	–	23	20	13
	MOLT-4	33	–	–	12	14
	RPMI-8226	52	13	–	15	14
	SR	44	16	11	41	28
Non-Small Cell Lung Cancer	A549/ATCC	53	25	13	10	25
	EKVX	17	–	–	–	–
	HOP-62	31	–	–	–	–
	HOP-92	–	–	–	–	–
	NCI-H226	18	12	–	–	19
	NCI-H23	24	–	–	–	–
	NCI-H322M	15	–	–	–	–
	NCI-H460	34	–	–	34	–
	NCI-H522	60	24	45	48	40
Colon Cancer	COLO 205	–	–	–	–	–
	HCC-2998	–	–	–	–	–
	HCT-116	51	–	17	26	21
	HCT-15	42	–	–	18	–
	HT29	43	18	14	25	23
	KM12	37	–	11	–	–
	SW-620	–	–	–	11	–
CNS Cancer	SF-268	–	–	–	–	–
	SF-295	17	–	–	–	–
	SF-539	–	–	–	–	–
	SNB-19	15	–	–	–	–
	SNB-75	–	–	–	–	–
	U251	31	–	–	24	–
Melanoma	LOX IMVI	37	24	34	–	21
	MALME-3M	–	–	–	–	–
	M14	45	38	37	–	38
	MDA-MB-435	19	–	–	–	–
	SK-MEL-2	–	–	–	–	–
	SK-MEL-28	20	–	–	–	13
	SK-MEL-5	32	38	19	–	38
	UACC-257	38	29	15	–	26
	UACC-62	40	29	20	–	27
Ovarian Cancer	IGROV1	–	–	–	–	–
	OVCAR-3	41	–	–	–	–
	OVCAR-4	30	–	–	–	–
	OVCAR-5	–	–	–	–	–
	OVCAR-8	29	–	–	–	–
	NCI/ADR-RES	20	–	–	–	–
	SK-OV-3	13	–	–	–	–
Renal Cancer	786-0	–	–	–	–	–
	A498	–	–	–	–	–
	RXF 393	–	–	–	–	21
	SN12C	26	–	–	–	–
	TK-10	–	–	–	–	–
	UO-31	15	–	–	–	–
Prostate	PC-3	55	–	–	20	–
	DU-145	11	–	–	–	–
Breast Cancer	MCF7	30	20	–	20	23
	MDA-MB-231	28	–	–	–	–
	HS 578T	–	–	–	–	–
	BT-549	–	–	–	–	–
	T-47D	45	–	–	13	–
	MDA-MB-468	32	–	–	–	–
Sensitive cell lines no.		42	13	12	17	19

a Only GI% higher than 10% are shown.

**Table 2.** Percentage growth inhibition (GI%) of *in vitro* subpanel tumor cell lines at 10 μM concentration for pyridines **5g**–**j** and **5l**.

Subpanel/Cell Line		Compound^a^				
		**5g**	**5h**	**5i**	**5j**	**5l**
Leukemia	CCRF-CEM	–	10	–	50	60
	HL-60(TB)	24	15	20	10	44
	K-562	21	22	–	42	68
	MOLT-4	18	29	15	44	80
	RPMI-8226	–	24	–	56	55
	SR	22	18	–	44	54
Non-Small Cell Lung Cancer	A549/ATCC	32	32	29	35	61
	EKVX	–	–	–	–	23
	HOP-62	–	–	–	–	50
	HOP-92	–	–	–	–	–
	NCI-H226	–	–	–	–	–
	NCI-H23	–	16	–	–	22
	NCI-H322M	–	–	–	17	18
	NCI-H460	–	–	–	43	82
	NCI-H522	38	39	28	52	41
Colon Cancer	COLO 205	–	–	–	–	53
	HCC-2998	–	14	–	–	27
	HCT-116	–	22	–	27	74
	HCT-15	–	26	–	30	71
	HT29	11	12	–	19	65
	KM12	–	18	–	18	51
	SW-620	–	–	–	–	54
CNS Cancer	SF-268	–	–	–	–	42
	SF-295	–	–	–	–	51
	SF-539	–	–	–	–	54
	SNB-19	–	–	–	–	42
	SNB-75	–	–	–	–	46
	U251	–	–	–	21	65
Melanoma	LOX IMVI	34	–	–	10	86
	MALME-3M	–	–	–	–	–
	M14	29	–	–	38	42
	MDA-MB-435	–	–	–	–	41
	SK-MEL-2	–	24	–	11	10
	SK-MEL-28	–	–	–	–	38
	SK-MEL-5	17	12	–	22	48
	UACC-257	25	20	23	–	25
	UACC-62	27	18	19	–	43
Ovarian Cancer	IGROV1	–	–	–	–	40
	OVCAR-3	–	–	–	–	46
	OVCAR-4	–	–	–	–	25
	OVCAR-5	–	–	–	–	–
	OVCAR-8	–	–	–	–	59
	NCI/ADR-RES	–	–	–	–	33
	SK-OV-3	–	–	–	–	36
Renal Cancer	786-0	–	–	–	12	40
	A498	–	–	–	–	–
	RXF 393	–	–	–	–	48
	SN12C	–	–	–	–	36
	TK-10	–	–	–	–	31
	UO-31	–	13	–	–	36
Prostate	PC-3	–	19	–	52	51
	DU-145	–	–	–	–	34
Breast Cancer	MCF7	–	22	12	21	75
	MDA-MB-231	–	–	–	–	21
	HS 578T	–	–	–	–	–
	BT-549	–	–	–	15	33
	T-47D	–	15	13	–	26
	MDA-MB-468	–	16	17	13	23
Sensitive cell lines no.		12	23	9	24	52

a Only GI% higher than 10% are shown.

**Figure 2:**
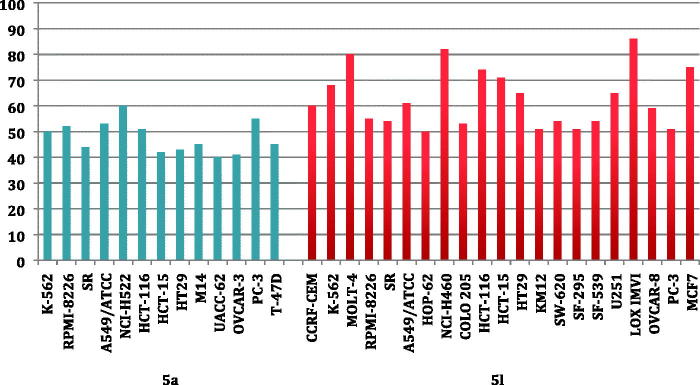
The most susceptible cancer cell lines towards the impact of target pyridines **5a** and **5l** according to the GI%.

Inspecting the GI% values in Tables 1 and 2, highlighted that compound **5l** stood out as the most potent pyridine derivative assayed in this study (mean % GI = 40). Pyridine **5l** possessed broad spectrum antitumor activity against all tested cancer cell lines from all subpanels with an exception to non-small cell lung cancer (HOP-92 and NCI-H226), melanoma (MALME-3M), ovarian cancer (OVCAR-5), renal cancer (A498) and breast cancer (HS 578T) cell lines. In particular, **5l** showed a potent growth inhibitory activity towards leukemia MOLT-4, non-small cell lung cancer NCI-H460, colon cancer HCT-116 and HCT-15, melanoma LOX IMVI and breast cancer MCF7 cell lines with inhibition % 80, 82, 74, 71, 86 and 75, respectively. In addition, it displayed GI more than 50% over leukemia (CCRF-CEM, K-562, RPMI-8226 and SR), non-small cell lung cancer (A549 and HOP-62), colon cancer (COLO205, HT29, KM12 and SW-620), CNS (SF-295, SF-539 and U251), ovarian (OVCAR-8 and prostate (PC-3) cell lines, Figure 2.

Furthermore, pyridine **5a** was found to be the second most active member (mean % GI = 22) with broad spectrum activity against 42 cell lines represent all subpanels. Compound **5a** exerted cytotoxic activity with GI more than 40% against leukemia (K-562, RPMI-8226 and SR), non-small cell lung cancer (A549 and NCI-H522), colon cancer (HCT-116, HCT-15 and HT29), melanoma (M14 and UACC-62), ovarian (OVCAR-3), prostate (PC-3) and breast (T-47D) cell lines (Figure 2).

Further investigation of results in Tables 1 and 2 unveiled that all cell lines of the leukemia subpanel were sensitive to six tested pyridines **5a, 5e, 5f, 5h, 5j and 5l** with GI ranging from 10% to 91%. It is noteworthy that only non-small cell lung cancer A549 and NCI-H522 cells were sensitive to all the tested pyridines with GI% range of 10–61% and 24–60%, respectively. Additionally, leukemia SR (except **5i**), leukemia HL-60 (except **5d**) and colon cancer HT29 (except **5i**) cell line were susceptible to nine tested pyridines. The most susceptible cell lines towards the impact of pyridines **5a** and **5l** are displayed in Figure 2.

#### In vitro anti-proliferative activity against A549 and HCT-116 cell lines

All newly synthesized pyridines **5a–l** were examined for their anti-proliferative activity towards two cancer cell lines: non-small cell lung cancer A549 cell line and colon cancer HCT-116 cell line. The MTT colorimetric assay was adopted to assess the anti-proliferative activity as described by Mosmann^29^. Doxorubicin was used as a control in this assay. The results were expressed as median growth inhibitory concentration (IC_50_) values that represent the compound concentration required to produce a 50% inhibition of cell growth after 48 h of incubation (Table 3).

**Table 3.** *In vitro* anti-proliferative activity of target pyridines **5a–l** against A549 and HCT-116 cell lines. 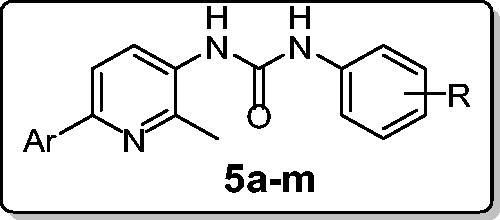

Compound	Ar	R	IC_50_ (µM)^a^	
			A549	HCT-116
**5a**	4-F-C_6_H_4_	3-CF_3_	6.83 ± 0.42	5.49 ± 0.30
**5b**	4-F-C_6_H_4_	3,5-(CF_3_)_2_	24.05 ± 1.78	16.03 ± 1.52
**5c**	4-F-C_6_H_4_	4-COOEt	9.61 ± 1.03	NT^b^
**5d**	4-F-C_6_H_4_	3,4-Methylenedioxy	12.48 ± 0.85	10.37 ± 0.84
**5e**	4-Cl-C_6_H_4_	3-CF_3_	11.87 ± 0.92	7.05 ± 0.72
**5f**	4-Cl-C_6_H_4_	4-OCH_3_	7.90 ± 0.54	12.61 ± 1.08
**5g**	4-Cl-C_6_H_4_	3,4-Methylenedioxy	6.72 ± 0.38	NT^b^
**5h**	2-thienyl	4-F	10.64 ± 0.86	8.25 ± 0.84
**5i**	2-thienyl	4-Cl	8.73 ± 0.71	NT^b^
**5j**	2-thienyl	4-COOEt	8.04 ± 0.59	9.38 ± 0.67
**5k**	2-thienyl	3,4-Methylenedioxy	19.17 ± 2.05	16.43 ± 1.30
**5l**	2-thienyl	2-SO_2_NH_2_	3.22 ± 0.25	2.71 ± 0.16
**Dox.**	–	–	2.93 ± 0.28	3.10 ± 0.22

a IC_50_ values are the mean ± SD of three separate experiments.

b NA: Not tested.

The results of the MTT assay listed in Table 3 suggested that the examined pyridines **5a–l** exhibited excellent to moderate growth inhibitory activity against the tested A549 and HCT-116 cancer cell lines. Also, HCT-116 cells were found to be more sensitive to the impact of the tested compounds than A549 cells, except compound **5j** which is more effective towards A549 cells. Interestingly, compound **5l** emerged as the most active one towards both A549 and HCT-116 cell lines with IC_50_ values equal 3.22 ± 0.2 and 2.71 ± 0.16 µM, respectively, which are comparable to those of Doxorubicin: 2.93 ± 0.28and 3.10 ± 0.22, respectively.

Regarding activity against A549 cells, pyridines **5a**, **5c**, **5f**, **5g**, **5i** and **5j** displayed potent antitumor activity with IC_50_ values in the range of 6.72–9.61 μM, whereas the remaining tested pyridines exhibited moderate potency towards A549 cell line (IC_50_ range: 10.64–24.05 μM). On the other hand, investigation of the anti-proliferative activity against HCT-116 cell line elucidated that **5a**, **5e**, **5 h** and **5j** had potent anti-proliferative activity with IC_50_ values equal 5.49 ± 0.30, 7.05 ± 0.72, 8.25 ± 0.84 and 9.38 ± 0.67 μM, respectively. Furthermore, pyridines **5b**, **5d**, **5f** and **5k** were moderately active towards HCT-116 cells with IC_50_ values of 16.03 ± 1.52, 10.37 ± 0.84, 12.61 ± 1.08 and 16.43 ± 1.30 μM, respectively.

#### In vitro cytotoxicity towards non-tumorigenic human WI-38 cells

The cytotoxic activity of all synthesized pyridines **5a–l** were assessed against non-tumorigenic human lung fibroblast WI-38 cell line to investigate their safety, using the MTT colorimetric assay^29^. The results were expressed as IC_50_ values and the calculated selectivity index are presented in Table 4.

**Table 4.** Cytotoxicity of pyridines **5a–l** towards non-tumorigenic human lung fibroblast WI-38 cell line and their selectivity index (S. I.) towards lung A549 cancer cells.

Compound	IC_50_ (µM)^a^	S. I.
	WI-38	WI-38/A549
**5a**	93.55 ± 5.28	13.7
**5b**	151.37 ± 8.12	6.3
**5c**	122.61 ± 10.17	12.8
**5d**	107.28 ± 7.03	8.6
**5e**	130.44 ± 9.22	11.0
**5f**	115.86 ± 9.61	14.7
**5g**	63.48 ± 5.08	9.4
**5h**	142.60 ± 8.38	13.4
**5i**	129.31 ± 11.95	14.8
**5j**	107.29 ± 7.02	13.3
**5k**	138.74 ± 10.40	7.2
**5l**	67.05 ± 3.82	17.6

a IC_50_ values are the mean ± SD of three separate experiments.

The examined pyridines **5a–l** displayed non-significant cytotoxic impact towards human lung fibroblast WI-38 cell line with IC_50_ values spanning from 63.48 to 151.08 µM, thereby providing a good safety profile as anticancer agents with selectivity index range (6.3–17.6).

#### Induction of apoptosis in colorectal cancer HCT-116 cells

To investigate the mechanism of antitumor activity of the target pyridines and in continuation of our efforts to develop potent pro-apoptotic anticancer agents^35–39^, the ability of sulfonamide **5l** to provoke apoptosis in HCT-116 cells was evaluated.

#### Effects on mitochondrial apoptosis pathway proteins Bcl-2 and Bax

Bcl-2 and Bax are two discrete members of a gene family involved in the regulation of cellular apoptosis known as BcL-2 family, which finely tune the apoptotic switch on/off mechanism and considered as an important gatekeeper to the apoptotic response. While Bcl-2 protein is functionally characterized as an apoptosis-suppressing factor, the Bax protein is more functionally characterized as an apoptosis-promoting factor. So, the intracellular Bax/Bcl-2 ratio can profoundly influence the ability of a cell to respond to an apoptotic signal^40^^,^^41^.

In this study, treatment of HCT-116 cells with the IC_50_ of pyridine **5l** (IC_50_ = 3.22 ± 0.25 µM) resulted in a significant up-regulation of the expression level of the pro-apoptotic Bax protein by 6-fold compared to untreated control, with a concomitant significant decrease in the expression level of the anti-apoptotic Bcl-2 protein by approximately 75% compared to control (Table 5). These results revealed that pyridine **5l** significantly boosted the Bax/Bcl-2 ratio 25-fold in compared to control.

**Table 5.** Impact of pyridine **5l** on the expression levels of Bax and Bcl-2 in HCT-116 cancer cells treated with the compound at its IC_50_ concentration.

Comp.	Bax	Bcl-2	Bax/Bcl-2 ratio
	Pg/mL	ng/mL	
**5l**	256.7*	1.24*	207
Control	41.9	5.11	8.2

Data are represented as mean ± SD of three separate experiments.

* Significantly different from control at *p* < .05.

#### Effect on the level of cytochrome C

The interplay between the pro-apoptotic Bax and anti-apoptotic Bcl-2 proteins triggers the activated Bax to bind to the mitochondrial outer membrane which induces the opening of the mitochondrial voltage-dependent anion channel (VDAC), resulting in the release of cytochrome C from mitochondria into cytosol where it activates the caspase-dependent signaling and subsequent apoptosis. Involvement of cytochrome C release from mitochondria is an indicator of activation of the intrinsic apoptotic pathway^42^.

Herein, we assessed the expression level of cytochrome C to assure the adoption of the intrinsic pathway. As shown in Table 6, the level of cytochrome C was induced significantly higher (12-folds) in HCT-116 cells treated with pyridine **5l**, compared to untreated control (Table 6).

**Table 6.** Impact of pyridine **5l** on the expression levels of cytochrome C, p53, active caspases-3 and -9, in HCT-116 cancer cells treated with the compound at its IC_50_ concentration.

Comp.	Cyt-c	p53	Caspase-9	Caspase-3
	Pg/mL	Pg/mL	ng/mL	Pg/mL
**5l**	858*	961.2*	21.3*	458.4*
Control	67	44.3	2.34	46.8

Data are mean ± SD of three separate experiments.

* Significantly different from control at *p* < 0.05.

#### Effect on the level of p53

One of the major apoptosis signaling pathways involves the p53 tumor suppressor. The ability of p53 to control apoptosis in response to abnormal proliferative signals and stress is crucial for its tumor suppression role. p53 tumor suppressor protein is a nuclear transcription factor that regulates the expression of a wide variety of genes involved in apoptosis. p53 is able to induce Bax oligomerization and cytochrome c release from mitochondria^43^.

The effect of pyridine **5l** on p53 expression in HCT-116 cells was evaluated in this study. Results in Table 5 highlighted that treatment of HCT-116 cells with pyridine **5l** led to 21-fold enhanced expression levels of p53, compared to control (Table 6).

#### Effects on the levels of active caspase-3 and caspase-9

Caspases, cysteine-dependent aspartate-directed proteases, are key factors in apoptotic cell death that have been shown to play an important role in cleavage of vital structural and regulatory proteins important for cells survival, so activation of caspases is a hallmark for apoptosis induction^44^. The leading upstream caspases are caspase-9 in the intrinsic pathway and caspase-8 in the extrinsic pathway, where both converge to caspase-3 which is the key executioner of apoptosis^45^.

In comparison with the untreated control, the expression levels of active caspase-3 and caspases-9 in HCT-116 cells were 5.1- and 2.5-fold increased, respectively, in response to pyridine **5l** treatment with its IC_50_ concentration (Table 6).

#### Cell cycle analysis

**Figure 3:**
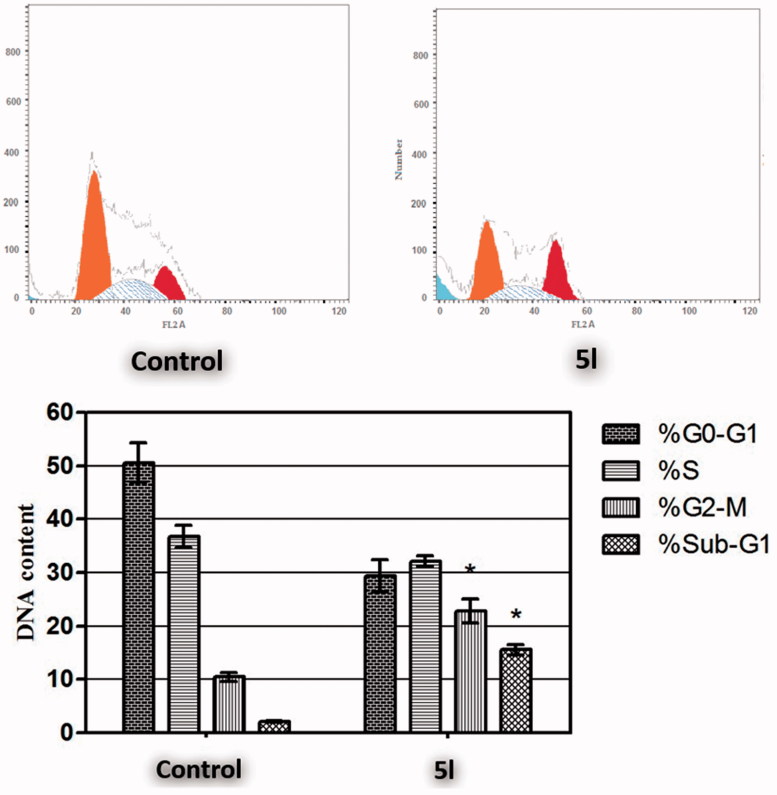
Effect of compound **5l** on the phases of cell cycle of HCT-116 cells. *Significantly different from control at *p* < 0.05. (Two-way ANOVA test).

Targeting the cell cycle of cancer cells has emerged as a promising approach for cancer therapy^46^. In the current study, pyridine **5l** was examined for its effect on the cell cycle distribution in HCT-116 cells (Figure 3). The results of the DNA flow cytometric assay showed that treatment of HCT-116 cells with pyridine **5l** at its IC_50_ concentration for 24 h resulted in a significant 7.3-fold increased percentage of HCT-116 cells at Sub-G_1_, with concurrent significant reduction in the G_2_-M phase by approximately 2.2-fold. Both arrest of G_2_-M phase and alteration of the Sub-G_1_ phase are considered significant remarks for pyridine **5l** to induce apoptosis in HCT-116 cells.

#### AnnexinV-FITC/propidium iodide analysis of apoptosis

Translocation of phosphatidylserine (PS) from the inner to the outer membrane leaflet of the cell is an early apoptotic event, which could be detected by fluorescein-labeled annexinV (annexinV-FITC), a Ca^2+^-dependent phospholipid-binding protein with high affinity for PS. Combined with propidium iodide PI (an indicator of cell integrity), a measure of percentage cell population in early apoptosis can be achieved. Cells displaying increased annexinV-FITC fluorescence without a concurrent increase in PI fluorescence are considered to be in early apoptosis, whereas an increase is seen in both fluorescence channels, signifies a late apoptosis^47^.

**Figure 4:**
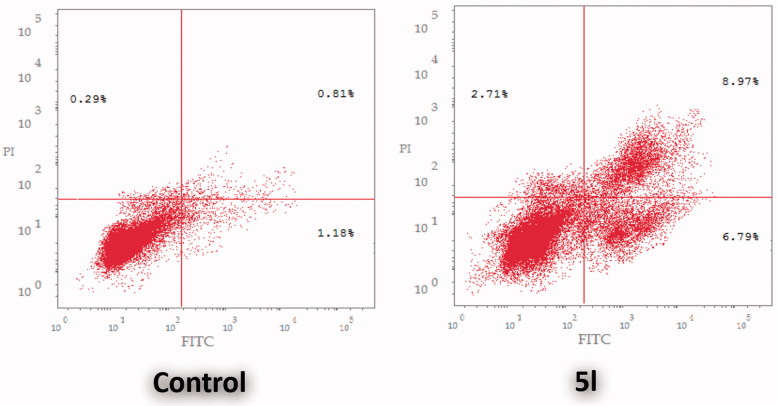
Effect of sulfonamide **5l** on the percentage of annexin V-FITC-positive staining in HCT-116 cells. The experiments were done in triplicates. The four quadrants identified as: **LL:** viable; **LR:** early apoptotic; **UR:** late apoptotic; **UL:** necrotic.

In this study, AnnexinV-FITC/PI dual staining assay was performed to evaluate the effect of compound **5l** on both early and late apoptosis percentages in HCT-116 cells (Figure 4, Table 7). As presented in Figure 4, the assay outcomes clearly indicate that the treatment of HCT-116 cells with **5l** resulted in a significant increase in the percentage of annexinV-FITC-positive apoptotic cells, including both the early and late apoptotic phases (LR; from 1.18% to 6.79%, and UR; from 0.81% to 8.97%), that represents about eightfold total increase in comparison with control (Table 7).

**Table 7.** Distribution of apoptotic cells in the annexin V-FITC experiment.

Comp.	Early Apoptosis	Late Apoptosis	Total
	(Lower Right %)	(Upper Right %)	(L.R % + U.R %)
**5l**	6.79	8.97	15.76
Control	1.18	0.81	1.99

## Conclusion

In summary, herein we report the synthesis of novel series of 1-(2-methyl-6-arylpyridin-3-yl)-3-phenylureas **5a–l**. All the prepared pyridins were evaluated for their *in vitro* anticancer activity against two cancer cell lines: non-small cell lung cancer A549 cell line and colon cancer HCT-116 cell line. Compound **5l** was found to be the most active congener towards both A549 and HCT-116 cell lines with IC_50_ values equal to 3.22 ± 0.2 and 2.71 ± 0.16 µM, respectively, which are comparable with those of Doxorubicin: 2.93 ± 0.28 and 3.10 ± 0.22, respectively. Furthermore, compound **5l** stood out as the most potent pyridine derivative (mean % GI = 40), at US-NCI Developmental Therapeutic Program anticancer assay, with broad-spectrum antitumor activity against the most tested cancer cell lines from all subpanels. The ability of sulfonamide **5l** to provoke apoptosis in HCT-116 cells was evaluated. Results revealed that pyridine **5l** significantly boosted the Bax/Bcl-2 ratio 25-fold compared to control. Also, the expression levels of cytochrome C, p53, active caspase-3 and caspases-9 in HCT-116 cells were 12-, 21-, 5.1- and 2.5-fold increased, respectively, in response to pyridine **5l** treatment. Furthermore, treatment of HCT-116 cells with pyridine **5l** at its IC_50_ concentration resulted in a significant 7.3-fold increased percentage of HCT-116 cells at Sub-G_1_, with concurrent significant reduction in the G_2_-M phase by approximately 2.2-fold, in addition to a significant increase in the percentage of annexinV-FITC-positive apoptotic cells, including both the early and late apoptotic phases (LR; from 1.18% to 6.79%, and UR; from 0.81% to 8.97%) that represent about eightfold total increase in comparison with control.

## Supplementary Material

Supplemental Material

Supplemental Material

## References

[1] Delbridge AR, Grabow S, Strasser A, Vaux DL Thirty years of BCL-2: translating cell death discoveries into novel cancer therapies. Nat Rev Cancer 2016;16:99 26822577 10.1038/nrc.2015.17

[2] Hu W, Kavanagh JJ Anticancer therapy targeting the apoptotic pathway. Lancet Oncol 2003;4:721–9. 14662428 10.1016/s1470-2045(03)01277-4

[3] Lopez J, Tait SWG Mitochondrial apoptosis: killing cancer using the enemy within. Br J Cancer 2015;112:957 25742467 10.1038/bjc.2015.85 PMC4366906

[4] Wilhelm S, Dumas J, Ladouceur G, Lynch M, Scott W Diaryl ureas with kinase inhibiting activity. 2007; U.S. Pat 20070020704.

[5] Skårderud MR, Polk A, Vistisen KK, et al. Efficacy and safety of regorafenib in the treatment of metastatic colorectal cancer: a systematic review. Cancer Treat Rev 2017;62:61–73. 29175677 10.1016/j.ctrv.2017.10.011

[6] Zhang L, Yu J Role of apoptosis in colon cancer biology, therapy, and prevention. Curr Colorectal Cancer Rep 2013;9:331–40. 10.1007/s11888-013-0188-z PMC3836193 24273467

[7] Chen D, Wei L, Yu J, Zhang L Regorafenib inhibits colorectal tumor growth through PUMA-mediated apoptosis. Clin Cancer Res. 2014;20:3472–84. 24763611 10.1158/1078-0432.CCR-13-2944 PMC4079733

[8] Sahu A, Prabhash K, Noronha V, et al. Crizotinib: a comprehensive review. South Asian J Cancer 2013;2:91. 24455567 10.4103/2278-330X.110506 PMC3876666

[9] Frampton JE Crizotinib: a review of its use in the treatment of anaplastic lymphoma kinase-positive, advanced non-small cell lung cancer. Drugs 2013;73:2031–51. 24288180 10.1007/s40265-013-0142-z

[10] Dai X, Guo G, Zou P, et al. (S)-crizotinib induces apoptosis in human non-small cell lung cancer cells by activating ROS independent of MTH1. J Exp Clin Cancer Res 2017;36:120. 28882182 10.1186/s13046-017-0584-3 PMC5590185

[11] El-Naggar M, Almahli H, Ibrahim HS, et al. Pyridine-ureas as potential anticancer agents: synthesis and in vitro biological evaluation. Molecules 2018;23:1459. 10.3390/molecules23061459 PMC6100082 29914120

[12] Eldehna WM, Fares M, Ibrahim HS, et al. Synthesis and biological evaluation of certain hydrazonoindolin-2-one derivatives as new potent antiproliferative agents. J Enz Inhib Med Chem 2018;33:867–78. 10.1080/14756366.2018.1462802 PMC7011955 29707975

[13] Sabt A, Abdelhafez OM, El-Haggar RS, et al. Novel coumarin-6-sulfonamides as apoptotic anti-proliferative agents: synthesis, *in vitro* biological evaluation, and QSAR studies. J Enz Inhib Med Chem 2018;33:1095–107. 10.1080/14756366.2018.1477137 PMC6022226 29944015

[14] Al-Ansary GH, Eldehna WM, Ghabbour HA, et al. Cancer stem cells CD133 inhibition and cytotoxicity of certain 3-phenylthiazolo [3, 2-a] benzimidazoles: design, direct synthesis, crystal study and *in vitro* biological evaluation. J Enzym Inhib Med Chem 2017;32:986–91. 10.1080/14756366.2017.1347166 PMC6010115 28726519

[15] Attia MI, Eldehna WM, Afifi SA, et al. New hydrazonoindolin-2-ones: synthesis, exploration of the possible antiproliferative mechanism of action and encapsulation into PLGA microspheres. PLoS One 2017;12:e0181241. 28742842 10.1371/journal.pone.0181241 PMC5526551

[16] Abdel-Aziz HA, Eldehna WM, Keeton AB, et al. Isatin-benzoazine molecular hybrids as potential antiproliferative agents: synthesis and in vitro pharmacological profiling. Drug Des Dev Ther 2017;11:2333. 10.2147/DDDT.S140164 PMC5557401 28848327

[17] Eldehna WM, Abou-Seri SM, El Kerdawy AM, et al. Increasing the binding affinity of VEGFR-2 inhibitors by extending their hydrophobic interaction with the active site: design, synthesis and biological evaluation of 1-substituted-4-(4-methoxybenzyl)phthalazine derivatives. Eur J Med Chem 2016;113:50–62. 26922228 10.1016/j.ejmech.2016.02.029

[18] Abdel-Aziz HA, Ghabbour HA, Eldehna WM, et al. 2-((Benzimidazol-2-yl)thio)-1-arylethan-1-ones: synthesis, crystal study and cancer stem cells CD133 targeting potential. Eur J Med Chem 2015;104:1–10. 26413725 10.1016/j.ejmech.2015.09.023

[19] Abdel-Aziz HA, Ghabbour HA, Eldehna WM, et al. Synthesis, crystal structure and biological activity of *cis*/*trans* amide rotomers of (*Z*)-*N*′-(2-oxoindolin-3-ylidene)formohydrazide. J Chem 2014;2014:1. doi:10.1155/2014/760434.

[20] Eldehna WM, Fares M, Ibrahim HS, et al. Synthesis and cytotoxic activity of biphenylurea derivatives containing indolin-2-one moieties. Molecules 2016;21:762. 10.3390/molecules21060762 PMC6274071 27294903

[21] Soliman DH, Eldehna WM, Ghabbour HA, et al. Novel 6-phenylnicotinohydrazide derivatives: design, synthesis and biological evaluation as a novel class of antitubercular and antimicrobial agents. Biol Pharm Bull 2017;40:1883–93. 29093335 10.1248/bpb.b17-00361

[22] Eldehna WM, Altoukhy A, Mahrous H, Abdel-Aziz HA Design, synthesis and QSAR study of certain isatin-pyridine hybrids as potential antiproliferative agents. Eur J Med Chem 2015;90:684–94. 25499988 10.1016/j.ejmech.2014.12.010

[23] Eldehna WM, Fares M, Abdel-Aziz MM, Abdel-Aziz HA Design, synthesis and antitubercular activity of certain nicotinic acid hydrazides. Molecules 2015;20:8800–15. 25988611 10.3390/molecules20058800 PMC6272317

[24] Boyd MR, Paull KD Some practical considerations and applications of the National Cancer Institute *in vitro* anticancer drug discovery screen. Drug Dev Res 1995;34:91–109.

[25] Boyd MR The NCI human tumor cell line (60-Cell) screen. In: Teicher BA, ed. Cancer drug discovery and development: anticancer drug development guide: preclinical screening, clinical trials and approval. Totowa, NJ: Humana Press; 2014:Chapter 1, 41–62.

[26] Monks A, Scudiero D, Skehan P, et al. Feasibility of a high-flux anticancer drug screen using a diverse panel of cultured human tumor cell lines. J Natl Cancer Inst 1991;83:757–66. 2041050 10.1093/jnci/83.11.757

[27] Skehan P, Storeng R, Scudiero D, et al. New colorimetric cytotoxicity assay for anticancer-drug screening. J. Natl Cancer Inst 1990;82:1107–12. 2359136 10.1093/jnci/82.13.1107

[28] Abou-Seri SM, Eldehna WM, Ali MM, El Ella DAA 1-Piperazinylphthalazines as potential VEGFR-2 inhibitors and anticancer agents: synthesis and *in vitro* biological evaluation. Eur J Med Chem 2016;107:165–79. 26590508 10.1016/j.ejmech.2015.10.053

[29] Mosmann T Rapid colorimetric assay for cellular growth and survival: application to proliferation and cytotoxicity assays. J Immunol Methods 1983;65:55–63. 6606682 10.1016/0022-1759(83)90303-4

[30] Ismail RSM, Abou-Seri SM, Eldehna WM, et al. Novel series of 6-(2-substitutedacetamido)-4-anilinoquinazolines as EGFR-ERK signal transduction inhibitors in MCF-7 breast cancer cells. Eur J Med Chem 2018;155:782–96. 30047410 10.1016/j.ejmech.2018.06.024

[31] Eldehna WM, Abo-Ashour MF, Nocentini A, et al. Novel 4/3-((4-oxo-5-(2-oxoindolin-3-ylidene) thiazolidin-2-ylidene) amino) benzenesulfonamides: synthesis, carbonic anhydrase inhibitory activity, anticancer activity and molecular modelling studies. Eur J Med Chem 2017;139:250–62. 28802125 10.1016/j.ejmech.2017.07.073

[32] Eldehna WM, Ibrahim HS, Abdel-Aziz HA, et al. Design, synthesis and *in vitro* antitumor activity of novel *N*-substituted-4-phenyl/benzylphthalazin-1-ones. Eur J Med Chem 2015;89:549–60. 25462265 10.1016/j.ejmech.2014.10.064

[33] Eldehna WM, Nocentini A, Al-Rashood ST, et al. Tumor-associated carbonic anhydrase isoform IX and XII inhibitory properties of certain isatin-bearing sulfonamides endowed with *in vitro* antitumor activity towards colon cancer. Bioorg Chem 2018;81:425–32. 30219719 10.1016/j.bioorg.2018.09.007

[34] Ghabbour HA, Qabeel MM, Eldehna WM, et al. Design, synthesis, and molecular docking of 1-(1-(4-chlorophenyl)-2-(phenylsulfonyl)ethylidene)-2-phenylhydrazine as potent nonazole anticandidal agent. J Chem 2014;2014:1.

[35] Eldehna WM, EL-Naggar DH, Hamed AR, et al. One-pot three-component synthesis of novel spirooxindoles with potential cytotoxic activity against triple-negative breast cancer MDA-MB-231 cells. J Enzym Inhib Med Chem 2018;33:309–18. 10.1080/14756366.2017.1417276 PMC6009943 29281924

[36] Almahli H, Hadchity E, Jaballah MY, et al. Development of novel synthesized phthalazinone-based PARP-1 inhibitors with apoptosis inducing mechanism in lung cancer. Bioorg Chem 2018;77:443–56. 29453076 10.1016/j.bioorg.2018.01.034

[37] Eldehna WM, Abo-Ashour MF, Ibrahim HS, et al. [(3-indolylmethylene)hydrazono]indolin-2-ones as apoptotic anti-proliferativeagents: design, synthesis and *in vitro* biological evaluation. J Enzym Inhib Med Chem 2018;33:686–700. 10.1080/14756366.2017.1421181 PMC6010103 29560733

[38] El-Naggar M, Eldehna WM, Almahli H, et al. Novel thiazolidinone/thiazolo [3, 2-a] benzimidazolone-isatin conjugates as apoptotic anti-proliferative agents towards breast cancer: one-pot synthesis and in vitro biological evaluation. Molecules 2018;23:1420. 10.3390/molecules23061420 PMC6099623 29895744

[39] Abdel-Aziz HA, Eldehna WM, Ghabbour H, et al. Synthesis, crystal study, and anti-proliferative activity of some 2-benzimidazolylthioacetophenones towards triple-negative breast cancer MDA-MB-468 cells as apoptosis-inducing agents. Int J Mol Sci 2016;17:1221. 10.3390/ijms17081221 PMC5000619 27483243

[40] Youle RJ, Strasser A The BCL-2 protein family: opposing activities that mediate cell death. Nat Rev Mol Cell Biol 2008;9:47–59. 18097445 10.1038/nrm2308

[41] Jiang H, Zhao PJ, Su D, et al. Paris saponin I induces apoptosis via increasing the Bax/Bcl-2 ratio and caspase-3 expression in gefitinib-resistant non-small cell lung cancer *in vitro* and *in vivo*. Mol Med Rep 2014;9:2265–72. 24718383 10.3892/mmr.2014.2108

[42] Shimizu S, Narita M, Tsujimoto Y Bcl-2 family proteins regulate the release of apoptogenic cytochrome c by the mitochondrial channel VDAC. Nature 1999;399:483–7. 10365962 10.1038/20959

[43] Fridman JS, Lowe SW Control of apoptosis by p53. Oncogene 2003;22:9030–40. 14663481 10.1038/sj.onc.1207116

[44] McIlwain DR, Berger T, Mak TW Caspase functions in cell death and disease. Cold Spring Harb Perspect Biol 2013;5:a008656. 23545416 10.1101/cshperspect.a008656 PMC3683896

[45] Cohen GM Caspases: the executioners of apoptosis. Biochem J 1997;326:1–16. 9337844 10.1042/bj3260001 PMC1218630

[46] Evan GI, Vousden KH Proliferation, cell cycle and apoptosis in cancer. Nature 2001;411:342–8. 11357141 10.1038/35077213

[47] Vermes I, Haanen C, Steffens-Nakken H, Reutellingsperger C A novel assay for apoptosis flow cytometric detection of phosphatidylserine expression on early apoptotic cells using fluorescein labelled annexin V. J Immunol Methods 1995;184:39–51. 7622868 10.1016/0022-1759(95)00072-i

