# Supplementary material for: Synthesis and in vitro anticancer activity of certain novel 1-(2-methyl-6-arylpyridin-3-yl)-3-phenylureas as apoptosis-inducing agents
Source: J Enzyme Inhib Med Chem. 2019 Feb 5;34(1):322–32. doi: 10.1080/14756366.2018.1547286 (PMC6366416; doi:10.1080/14756366.2018.1547286)

Developmental Therapeutics Program One Dose Mean Graph	NSC: D-793909 / 1	Conc: 1.00E-5 Molar	Test Date: Nov 14, 2016
	Experiment ID: 1611OS15		Report Date: Dec 01, 2016

Panel/Cell Line	Growth Percent
**Leukemia**	
CCRF-CEM	75.01
HL-60(TB)	89.79
K-562	50.61
MOLT-4	67.98
RPMI-8226	48.50
SR	56.41
**Non-Small Cell Lung Cancer**	
A549/ATCC	47.62
EKVX	83.64
HOP-62	69.63
HOP-92	112.57
NCI-H226	82.60
NCI-H23	76.11
NCI-H322M	85.14
NCI-H460	66.82
NCI-H522	40.79
**Colon Cancer**	
COLO 205	113.32
HCC-2998	106.10
HCT-116	49.84
HCT-15	58.26
HT29	57.60
KM12	63.87
SW-620	101.74
**CNS Cancer**	
SF-268	94.80
SF-295	83.34
SF-539	102.58
SNB-19	85.66
SNB-75	91.86
U251	69.66
**Melanoma**	
LOX IMVI	63.78
MALME-3M	138.38
M14	55.47
MDA-MB-435	81.95
SK-MEL-2	90.71
SK-MEL-28	80.01
SK-MEL-5	68.68
UACC-257	62.66
UACC-62	60.67
**Ovarian Cancer**	
IGROV1	95.61
OVCAR-3	59.26
OVCAR-4	70.88
OVCAR-5	115.85
OVCAR-8	71.31
NCI/ADR-RES	80.28
SK-OV-3	87.65
**Renal Cancer**	
786-0	90.48
A498	87.40
RXF 393	108.41
SN12C	74.28
TK-10	95.03
UO-31	85.53
**Prostate Cancer**	
PC-3	45.02
DU-145	89.50
**Breast Cancer**	
MCF7	70.06
MDA-MB-231/ATCC	72.09
HS 578T	103.60
BT-549	97.30
T-47D	55.51
MDA-MB-468	68.77
Mean	78.59
Delta	37.80
Range	97.59

Mean Growth Percent - Growth Percent

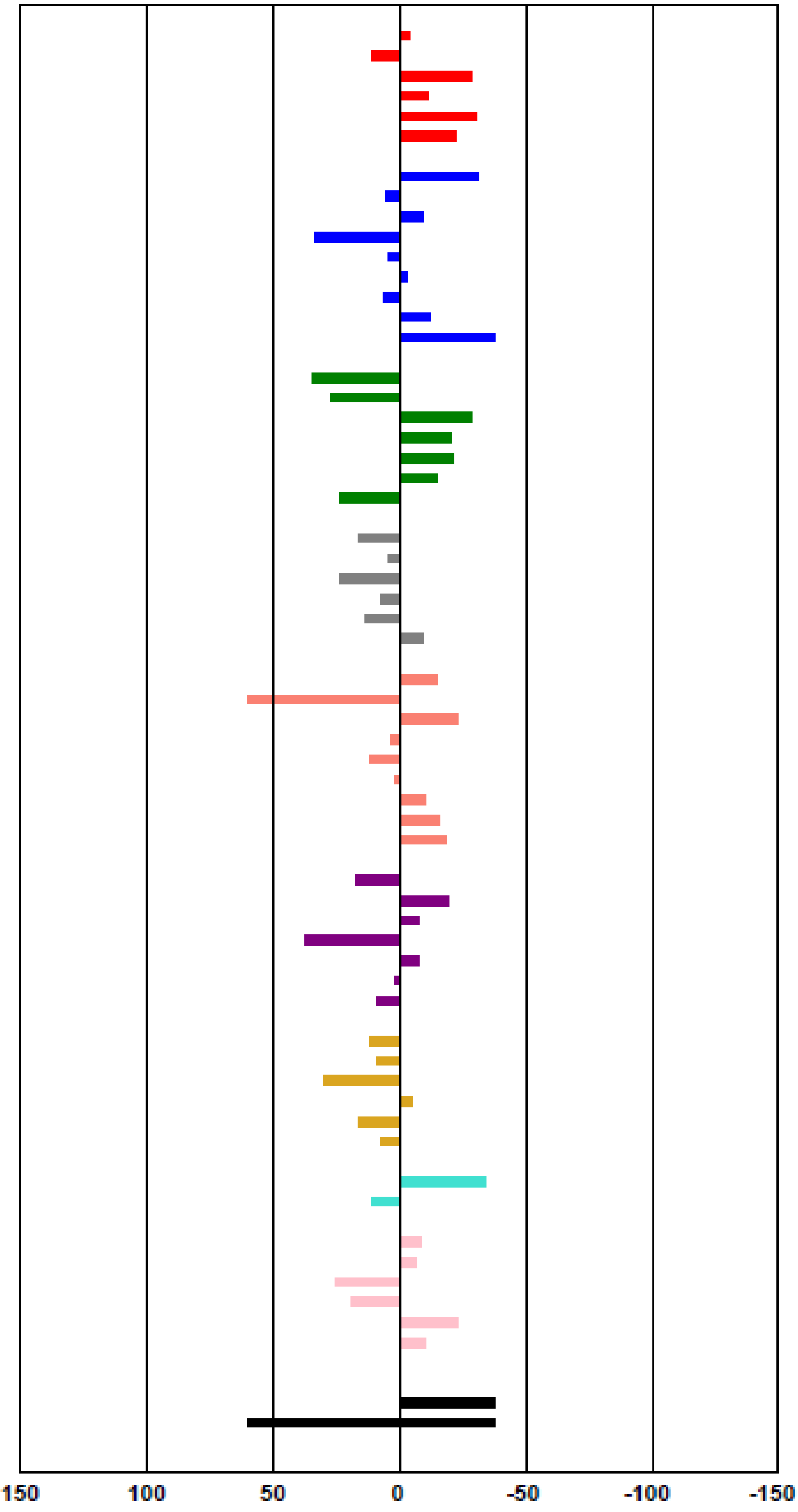

Developmental Therapeutics Program<br>One Dose Mean Graph	NSC: D-793920 / 1	Conc: 1.00E-5 Molar	Test Date: Nov 14, 2016
	Experiment ID: 1611OS15		Report Date: Dec 01, 2016

Panel/Cell Line	Growth Percent
**Leukemia**	
CCRF-CEM	98.62
HL-60(TB)	87.78
K-562	92.52
MOLT-4	100.33
RPMI-8226	87.90
SR	84.31
**Non-Small Cell Lung Cancer**	
A549/ATCC	75.94
EKVX	111.14
HOP-62	92.26
HOP-92	138.72
NCI-H226	88.92
NCI-H23	105.64
NCI-H322M	113.18
NCI-H460	96.60
NCI-H522	76.40
**Colon Cancer**	
COLO 205	105.51
HCC-2998	138.51
HCT-116	93.85
HCT-15	92.17
HT29	82.95
KM12	93.51
SW-620	117.53
**CNS Cancer**	
SF-268	108.18
SF-295	98.14
SF-539	96.70
SNB-19	101.62
SNB-75	113.91
U251	96.04
**Melanoma**	
LOX IMVI	76.97
MALME-3M	130.58
M14	62.57
MDA-MB-435	100.98
SK-MEL-2	97.05
SK-MEL-28	92.97
SK-MEL-5	62.21
UACC-257	71.05
UACC-62	71.72
**Ovarian Cancer**	
IGROV1	105.34
OVCAR-3	117.19
OVCAR-4	112.52
OVCAR-5	155.59
OVCAR-8	105.11
NCI/ADR-RES	107.90
SK-OV-3	97.79
**Renal Cancer**	
786-0	109.13
A498	127.05
RXF 393	98.61
SN12C	106.68
TK-10	107.97
UO-31	116.06
**Prostate Cancer**	
PC-3	97.32
DU-145	107.68
**Breast Cancer**	
MCF7	80.92
MDA-MB-231/ATCC	109.96
HS 578T	99.19
BT-549	115.80
T-47D	114.37
MDA-MB-468	105.12
Mean	100.90
Delta	38.69
Range	93.38

Mean Growth Percent - Growth Percent

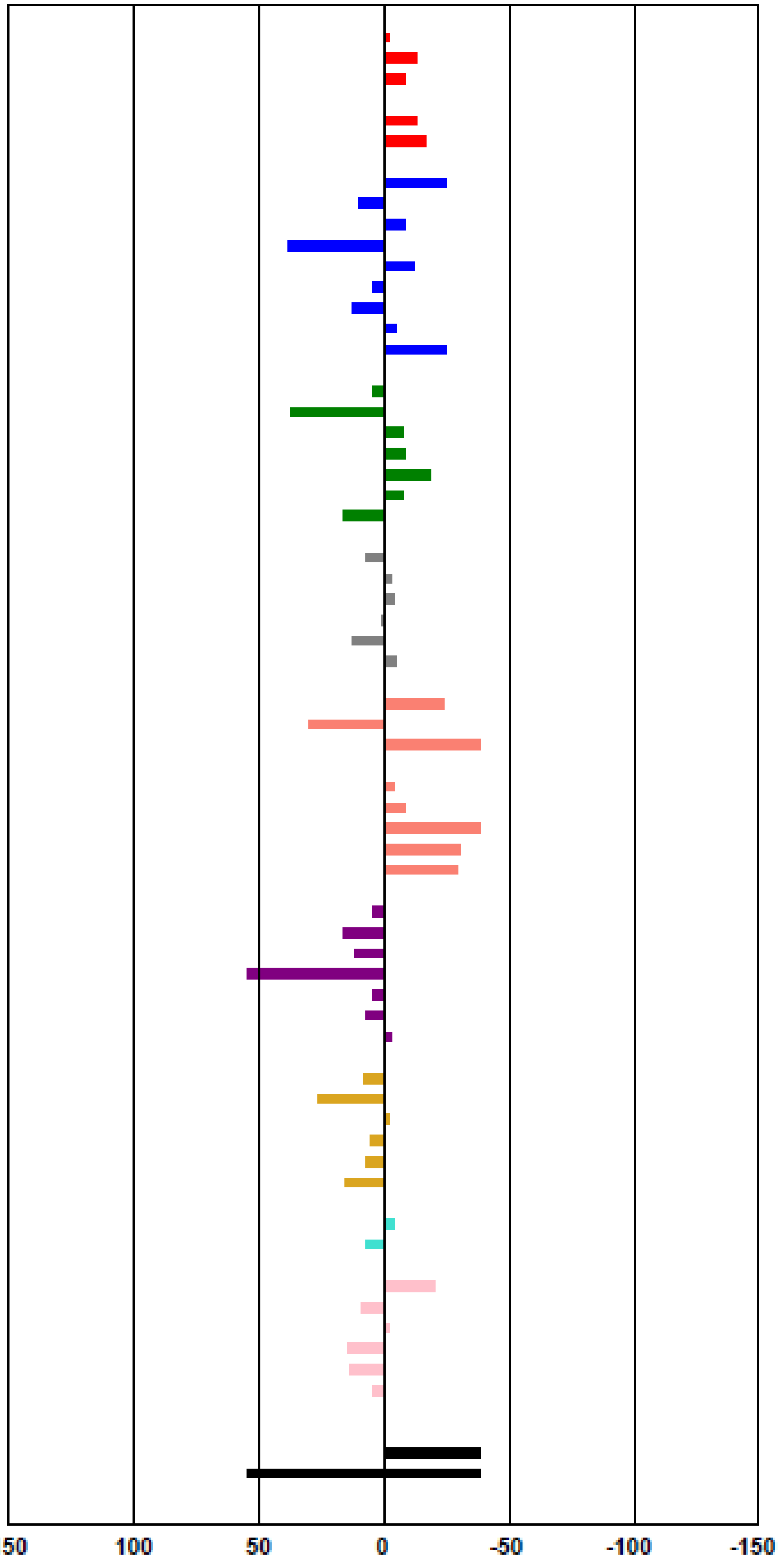

Developmental Therapeutics Program One Dose Mean Graph	NSC: D-793913 / 1	Conc: 1.00E-5 Molar	Test Date: Nov 14, 2016
	Experiment ID: 1611OS15		Report Date: Dec 01, 2016

Panel/Cell Line	Growth Percent
**Leukemia**	
CCRF-CEM	97.79
HL-60(TB)	92.35
K-562	77.32
MOLT-4	92.08
RPMI-8226	93.40
SR	89.05
**Non-Small Cell Lung Cancer**	
A549/ATCC	87.36
EKVX	112.03
HOP-62	94.14
HOP-92	125.22
NCI-H226	95.56
NCI-H23	108.71
NCI-H322M	107.06
NCI-H460	105.04
NCI-H522	55.64
**Colon Cancer**	
COLO 205	123.89
HCC-2998	106.33
HCT-116	83.80
HCT-15	98.36
HT29	86.48
KM12	89.38
SW-620	112.94
**CNS Cancer**	
SF-268	103.81
SF-295	102.50
SF-539	99.32
SNB-19	110.97
SNB-75	111.63
U251	96.49
**Melanoma**	
LOX IMVI	66.73
MALME-3M	149.08
M14	63.40
MDA-MB-435	105.65
SK-MEL-2	96.05
SK-MEL-28	92.12
SK-MEL-5	81.72
UACC-257	85.99
UACC-62	80.30
**Ovarian Cancer**	
IGROV1	116.82
OVCAR-3	112.87
OVCAR-4	129.18
OVCAR-5	136.39
OVCAR-8	111.52
NCI/ADR-RES	105.38
SK-OV-3	106.87
**Renal Cancer**	
786-0	102.21
A498	125.43
RXF 393	106.02
SN12C	100.09
TK-10	105.57
UO-31	104.30
**Prostate Cancer**	
PC-3	91.75
DU-145	109.03
**Breast Cancer**	
MCF7	90.95
MDA-MB-231/ATCC	119.47
HS 578T	106.69
BT-549	92.57
T-47D	100.36
MDA-MB-468	105.39
Mean	101.01
Delta	45.37
Range	93.44

Mean Growth Percent - Growth Percent

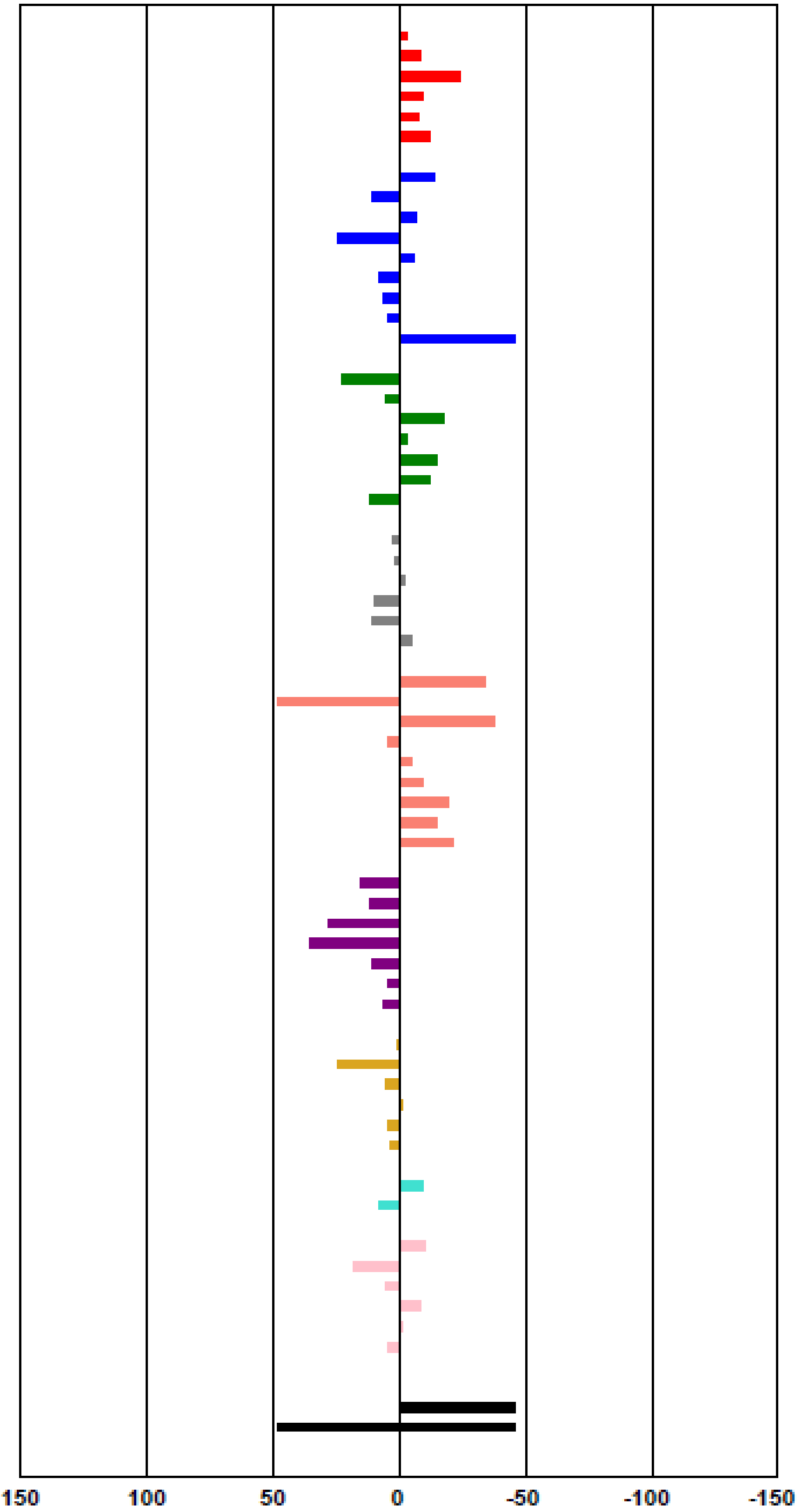

Developmental Therapeutics Program
One Dose Mean Graph

NSC: D-793911 / 1	Conc: 1.00E-5 Molar	Test Date: Nov 14, 2016
Experiment ID: 1611OS15		Report Date: Dec 01, 201

Panel/Cell Line	Growth Percent
**Leukemia**	
CCRF-CEM	78.61
HL-60(TB)	79.66
K-562	80.57
MOLT-4	88.22
RPMI-8226	85.33
SR	59.58
**Non-Small Cell Lung Cancer**	
A549/ATCC	93.12
EKVX	100.68
HOP-62	108.76
HOP-92	96.45
NCI-H226	97.89
NCI-H23	95.61
NCI-H322M	108.58
NCI-H460	64.46
NCI-H522	52.87
**Colon Cancer**	
COLO 205	120.84
HCC-2998	107.43
HCT-116	74.46
HCT-15	82.09
HT29	75.70
KM12	100.80
SW-620	89.56
**CNS Cancer**	
SF-268	98.36
SF-295	98.42
SF-539	94.13
SNB-19	107.92
SNB-75	92.83
U251	76.74
**Melanoma**	
LOX IMVI	92.87
MALME-3M	126.31
M14	100.78
MDA-MB-435	103.41
SK-MEL-2	95.39
SK-MEL-28	109.74
SK-MEL-5	95.30
UACC-257	98.91
UACC-62	95.60
**Ovarian Cancer**	
IGROV1	110.63
OVCAR-3	110.56
OVCAR-4	107.62
OVCAR-5	121.35
OVCAR-8	98.92
NCI/ADR-RES	98.15
SK-OV-3	110.00
**Renal Cancer**	
786-0	100.86
A498	95.16
RXF 393	104.08
SN12C	93.73
TK-10	90.66
UO-31	90.75
**Prostate Cancer**	
PC-3	80.47
DU-145	97.56
**Breast Cancer**	
MCF7	80.99
MDA-MB-231/ATCC	115.50
HS 578T	103.00
BT-549	98.64
T-47D	87.45
MDA-MB-468	98.52
Mean	95.22
Delta	42.35
Range	73.44

Mean Growth Percent - Growth Percent

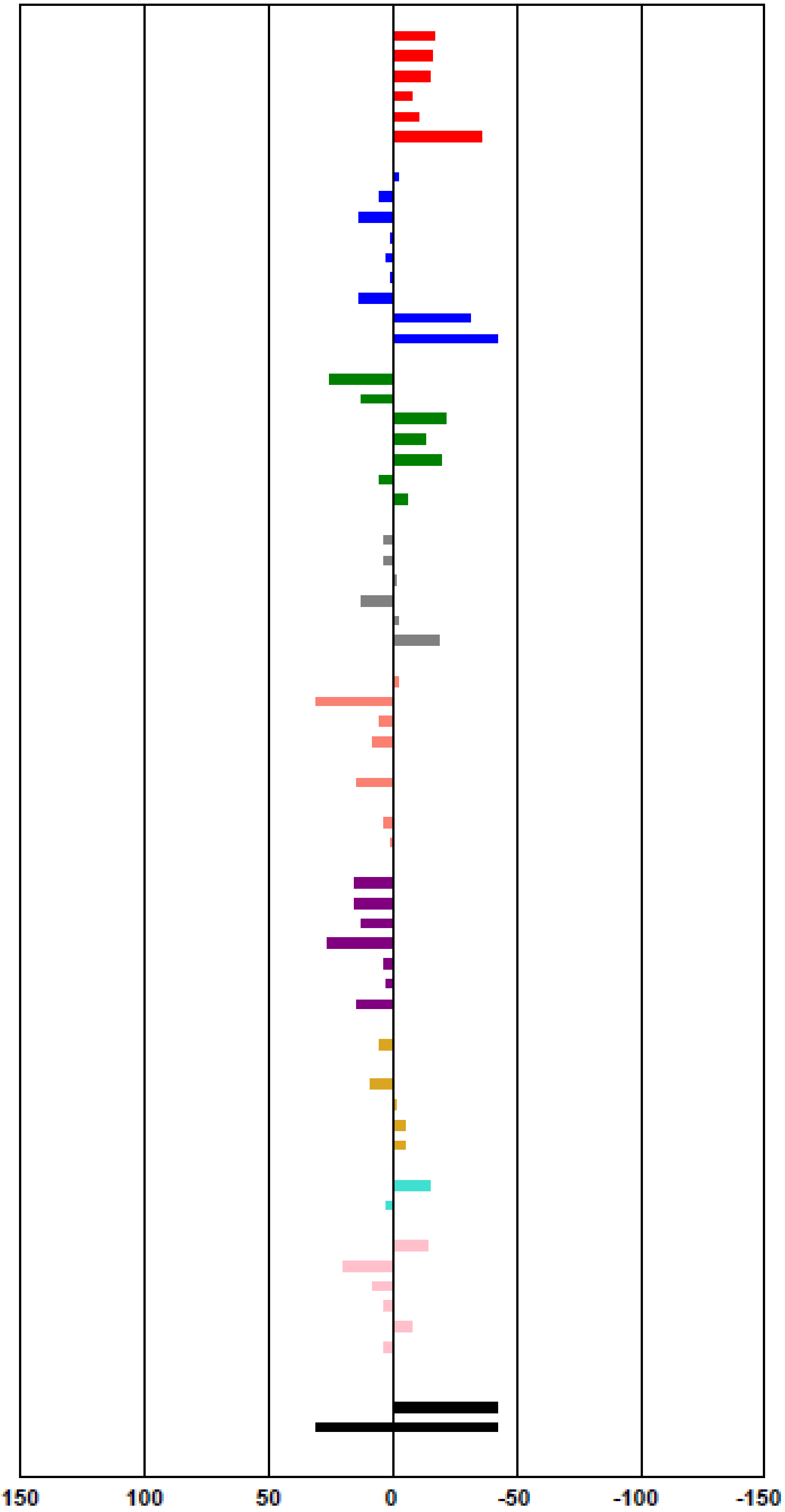

Developmental Therapeutics Program

One Dose Mean Graph

NSC: D-793923 / 1	Conc: 1.00E-5 Molar
Test Date: Nov 14, 2016	Experiment ID: 1611OS15
Report Date: Dec 01, 2016	

Panel/Cell Line	Growth Percent
**Leukemia**	
CCRF-CEM	82.08
HL-60(TB)	88.86
K-562	87.10
MOLT-4	86.53
RPMI-8226	86.09
SR	72.87
**Non-Small Cell Lung Cancer**	
A549/ATCC	75.91
EKVX	98.93
HOP-62	90.83
HOP-92	162.31
NCI-H226	81.63
NCI-H23	103.33
NCI-H322M	97.18
NCI-H460	102.45
NCI-H522	60.33
**Colon Cancer**	
COLO 205	103.65
HCC-2998	128.85
HCT-116	79.92
HCT-15	92.58
HT29	77.83
KM12	92.28
SW-620	115.16
**CNS Cancer**	
SF-268	102.17
SF-295	100.19
SF-539	106.31
SNB-19	108.44
SNB-75	117.24
U251	104.63
**Melanoma**	
LOX IMVI	79.60
MALME-3M	126.71
M14	62.03
MDA-MB-435	101.99
SK-MEL-2	93.36
SK-MEL-28	87.16
SK-MEL-5	62.55
UACC-257	74.92
UACC-62	73.73
**Ovarian Cancer**	
IGROV1	119.09
OVCAR-3	106.51
OVCAR-4	99.08
OVCAR-5	123.59
OVCAR-8	100.26
NCI/ADR-RES	108.29
SK-OV-3	106.19
**Renal Cancer**	
786-0	96.96
A498	95.30
RXF 393	79.69
SN12C	97.29
TK-10	107.91
UO-31	106.06
**Prostate Cancer**	
PC-3	97.20
DU-145	102.38
**Breast Cancer**	
MCF7	77.69
MDA-MB-231/ATCC	110.64
HS 578T	105.30
BT-549	100.59
T-47D	104.86
MDA-MB-468	91.51
Mean	96.62
Delta	36.29
Range	101.98

Mean Growth Percent - Growth Percent

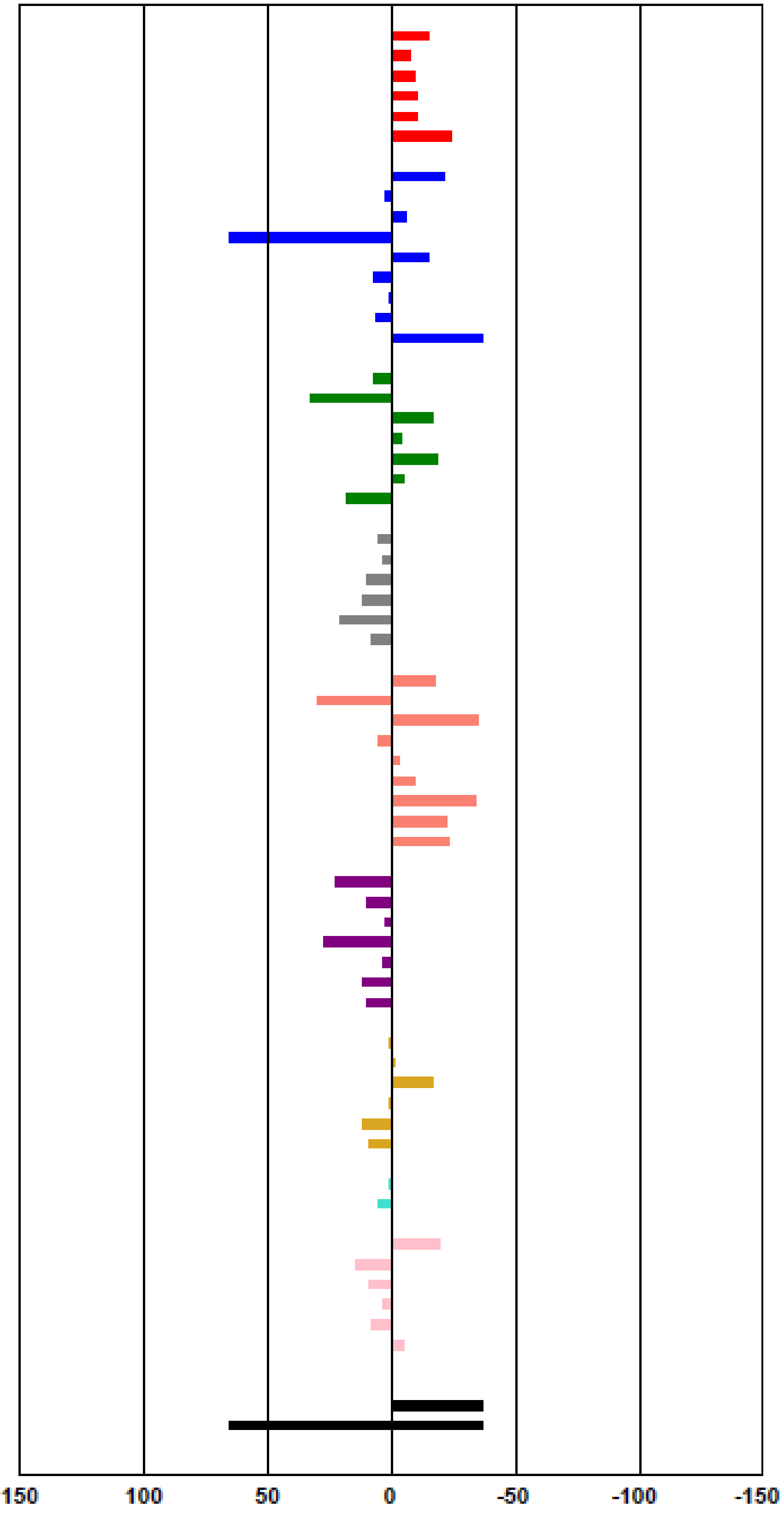

Developmental Therapeutics Program	NSC: D-793918 / 1	Conc: 1.00E-5 Molar	Test Date: Nov 14, 2016
One Dose Mean Graph	Experiment ID: 1611OS15		Report Date: Dec 01, 2016

Panel/Cell Line	Growth Percent
Leukemia	
CCRF-CEM	97.50
HL-60(TB)	76.33
K-562	79.97
MOLT-4	82.58
RPMI-8226	97.87
SR	78.31
Non-Small Cell Lung Cancer	
A549/ATCC	68.34
EKVX	114.01
HOP-62	98.95
HOP-92	152.05
NCI-H226	101.05
NCI-H23	106.37
NCI-H322M	102.86
NCI-H460	110.69
NCI-H522	62.72
Colon Cancer	
COLO 205	129.40
HCC-2998	127.28
HCT-116	103.37
HCT-15	101.46
HT29	89.61
KM12	92.20
SW-620	119.03
CNS Cancer	
SF-268	103.12
SF-295	99.78
SF-539	103.16
SNB-19	111.44
SNB-75	101.05
U251	96.47
Melanoma	
LOX IMVI	64.48
MALME-3M	155.82
M14	71.38
MDA-MB-435	108.58
SK-MEL-2	103.47
SK-MEL-28	95.23
SK-MEL-5	83.70
UACC-257	75.15
UACC-62	73.92
Ovarian Cancer	
IGROV1	110.56
OVCAR-3	111.66
OVCAR-4	99.08
OVCAR-5	133.01
OVCAR-8	105.45
NCI/ADR-RES	101.70
SK-OV-3	108.43
Renal Cancer	
786-0	91.83
A498	111.22
RXF 393	107.57
SN12C	102.48
TK-10	101.67
UO-31	97.53
Prostate Cancer	
PC-3	95.56
DU-145	108.43
Breast Cancer	
MCF7	91.22
MDA-MB-231/ATCC	108.64
HS 578T	103.84
BT-549	105.91
T-47D	101.47
MDA-MB-468	101.62
Mean	100.65
Delta	37.93
Range	93.10

Mean Growth Percent - Growth Percent

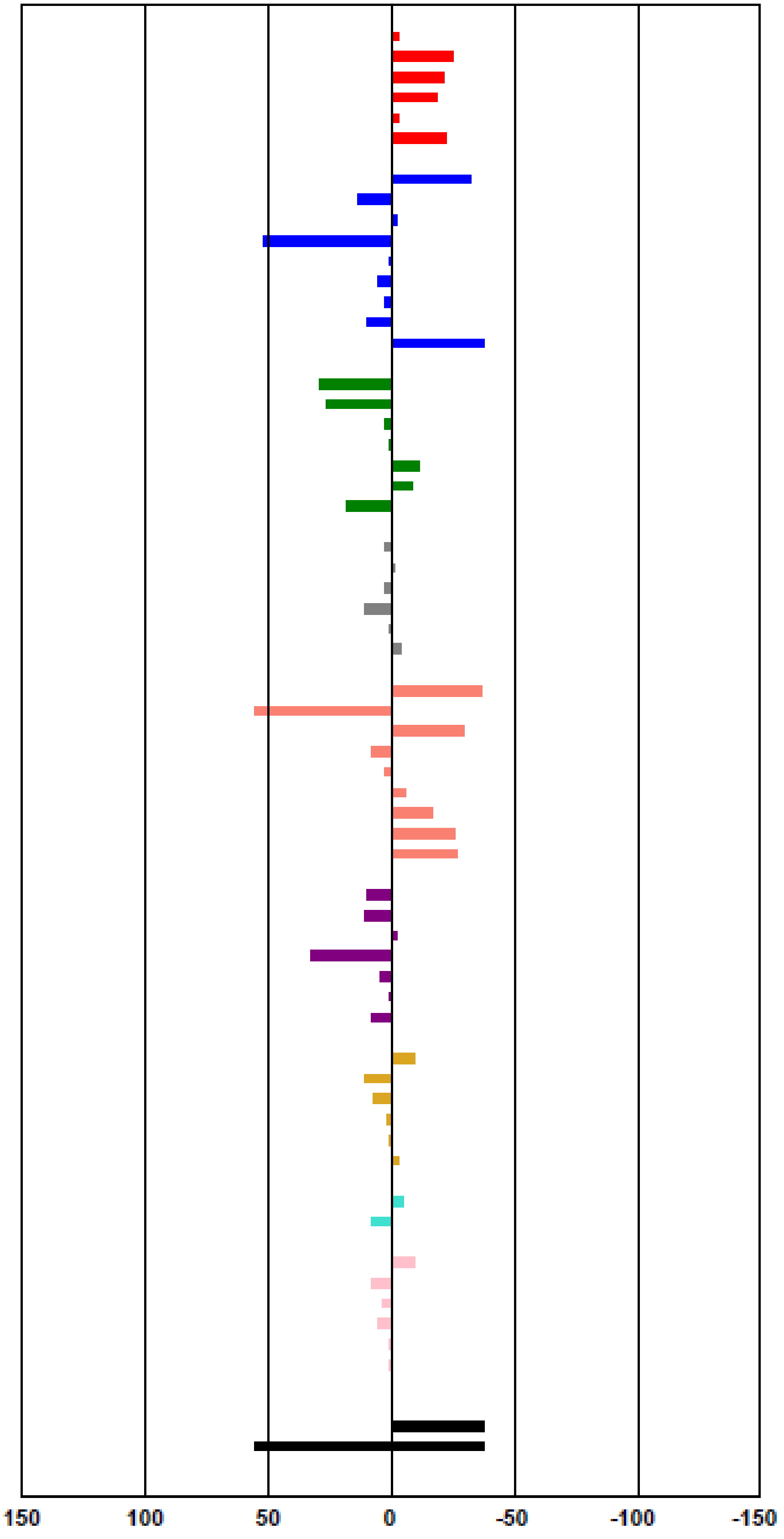

Developmental Therapeutics Program One Dose Mean Graph	NSC: D-793922 / 1	Conc: 1.00E-5 Molar	Test Date: Nov 14, 2016
	Experiment ID: 1611OS15		Report Date: Dec 01, 2016

Panel/Cell Line	Growth Percent
**Leukemia**	
CCRF-CEM	90.48
HL-60(TB)	85.82
K-562	78.51
MOLT-4	71.52
RPMI-8226	76.70
SR	82.76
**Non-Small Cell Lung Cancer**	
A549/ATCC	68.68
EKVX	102.07
HOP-62	105.75
HOP-92	134.33
NCI-H226	95.19
NCI-H23	84.36
NCI-H322M	104.78
NCI-H460	101.60
NCI-H522	61.67
**Colon Cancer**	
COLO 205	117.78
HCC-2998	86.14
HCT-116	78.39
HCT-15	74.35
HT29	88.55
KM12	82.88
SW-620	104.95
**CNS Cancer**	
SF-268	103.08
SF-295	100.75
SF-539	106.52
SNB-19	109.02
SNB-75	103.11
U251	93.25
**Melanoma**	
LOX IMVI	96.36
MALME-3M	134.60
M14	95.02
MDA-MB-435	104.66
SK-MEL-2	76.38
SK-MEL-28	102.30
SK-MEL-5	88.12
UACC-257	80.98
UACC-62	82.90
**Ovarian Cancer**	
IGROV1	116.05
OVCAR-3	89.82
OVCAR-4	95.44
OVCAR-5	120.99
OVCAR-8	96.50
NCI/ADR-RES	97.38
SK-OV-3	106.27
**Renal Cancer**	
786-0	95.90
A498	94.94
RXF 393	119.79
SN12C	100.06
TK-10	104.39
UO-31	87.82
**Prostate Cancer**	
PC-3	81.06
DU-145	107.97
**Breast Cancer**	
MCF7	78.30
MDA-MB-231/ATCC	113.99
HS 578T	105.11
BT-549	104.14
T-47D	85.14
MDA-MB-468	84.15
Mean	95.51
Delta	33.84
Range	72.93

Mean Growth Percent - Growth Percent

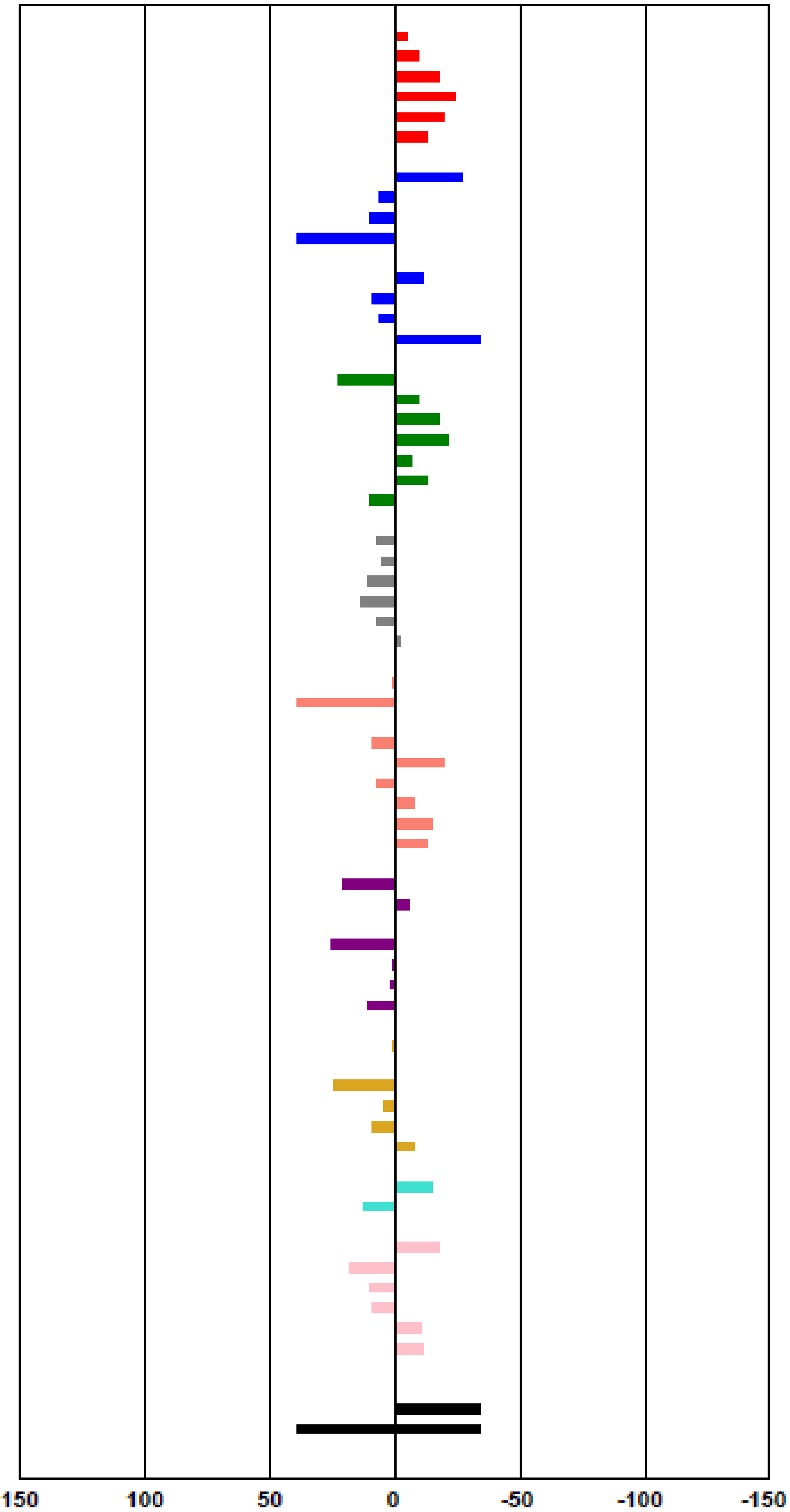

Developmental Therapeutics Program
One Dose Mean Graph

NSC: D-793910 / 1	Conc: 1.00E-5 Molar	Test Date: Nov 14, 2016
Experiment ID: 1611OS15		Report Date: Dec 01, 2016

Panel/Cell Line	Growth Percent
**Leukemia**	
CCRF-CEM	95.31
HL-60(TB)	80.37
K-562	93.81
MOLT-4	85.93
RPMI-8226	102.86
SR	94.25
**Non-Small Cell Lung Cancer**	
A549/ATCC	71.62
EKVX	94.87
HOP-62	93.23
HOP-92	112.02
NCI-H226	106.24
NCI-H23	98.88
NCI-H322M	93.45
NCI-H460	102.09
NCI-H522	72.79
**Colon Cancer**	
COLO 205	120.46
HCC-2998	116.19
HCT-116	100.35
HCT-15	93.10
HT29	93.01
KM12	98.36
SW-620	101.11
**CNS Cancer**	
SF-268	101.36
SF-295	98.15
SF-539	97.77
SNB-19	102.13
SNB-75	89.50
U251	91.28
**Melanoma**	
LOX IMVI	98.62
MALME-3M	116.31
M14	98.74
MDA-MB-435	99.27
SK-MEL-2	98.32
SK-MEL-28	95.64
SK-MEL-5	97.79
UACC-257	77.86
UACC-62	81.45
**Ovarian Cancer**	
IGROV1	111.47
OVCAR-3	91.55
OVCAR-4	92.63
OVCAR-5	102.68
OVCAR-8	101.15
NCI/ADR-RES	103.77
SK-OV-3	96.01
**Renal Cancer**	
786-0	93.54
A498	97.56
RXF 393	112.01
SN12C	98.42
TK-10	99.56
UO-31	93.27
**Prostate Cancer**	
PC-3	92.28
DU-145	101.21
**Breast Cancer**	
MCF7	88.32
MDA-MB-231/ATCC	101.47
HS 578T	103.10
BT-549	105.13
T-47D	87.32
MDA-MB-468	83.69
Mean	96.91
Delta	25.29
Range	48.84

Mean Growth Percent - Growth Percent

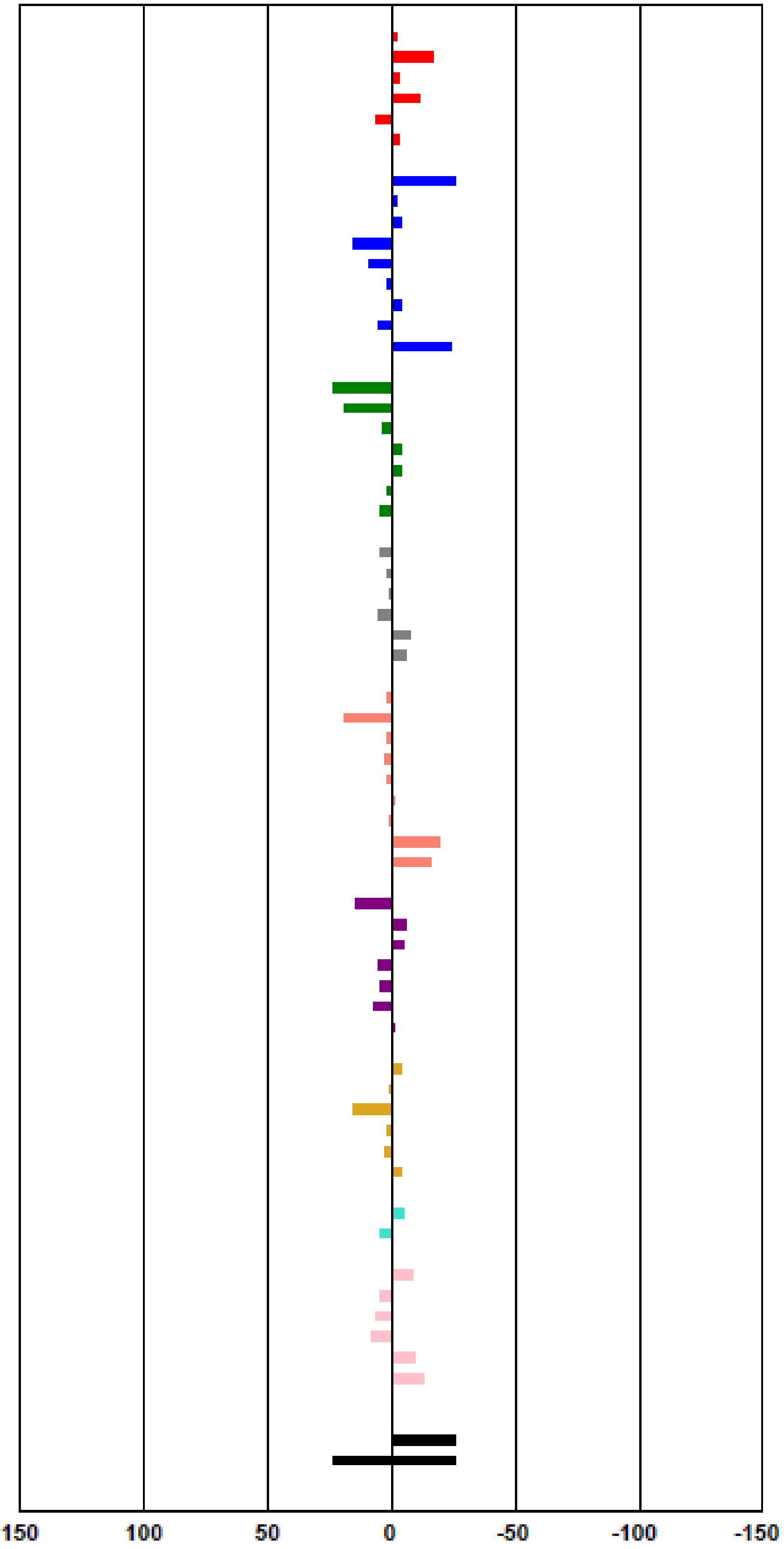

Developmental Therapeutics Program One Dose Mean Graph	NSC: D-793921 / 1	Conc: 1.00E-5 Molar	Test Date: Nov 14, 2016
	Experiment ID: 1611OS15		Report Date: Dec 01, 2016

Panel/Cell Line	Growth Percent
Leukemia	
CCRF-CEM	50.50
HL-60(TB)	90.80
K-562	58.87
MOLT-4	56.13
RPMI-8226	44.61
SR	54.38
Non-Small Cell Lung Cancer	
A549/ATCC	65.69
EKVX	101.35
HOP-62	93.65
HOP-92	108.87
NCI-H226	104.45
NCI-H23	100.46
NCI-H322M	83.05
NCI-H460	57.61
NCI-H522	48.56
Colon Cancer	
COLO 205	117.91
HCC-2998	107.56
HCT-116	73.12
HCT-15	70.19
HT29	81.99
KM12	82.73
SW-620	111.46
CNS Cancer	
SF-268	100.98
SF-295	99.17
SF-539	98.43
SNB-19	103.60
SNB-75	104.20
U251	79.14
Melanoma	
LOX IMVI	90.58
MALME-3M	128.24
M14	62.31
MDA-MB-435	108.03
SK-MEL-2	89.77
SK-MEL-28	94.78
SK-MEL-5	78.41
UACC-257	91.86
UACC-62	94.03
Ovarian Cancer	
IGROV1	109.52
OVCAR-3	91.15
OVCAR-4	124.76
OVCAR-5	120.46
OVCAR-8	105.27
NCI/ADR-RES	107.40
SK-OV-3	102.46
Renal Cancer	
786-0	88.54
A498	102.38
RXF 393	106.67
SN12C	102.07
TK-10	96.44
UO-31	98.68
Prostate Cancer	
PC-3	48.80
DU-145	108.42
Breast Cancer	
MCF7	79.88
MDA-MB-231/ATCC	110.34
HS 578T	115.62
BT-549	85.00
T-47D	92.68
MDA-MB-468	87.85
Mean	90.89
Delta	46.28
Range	83.63

Mean Growth Percent - Growth Percent

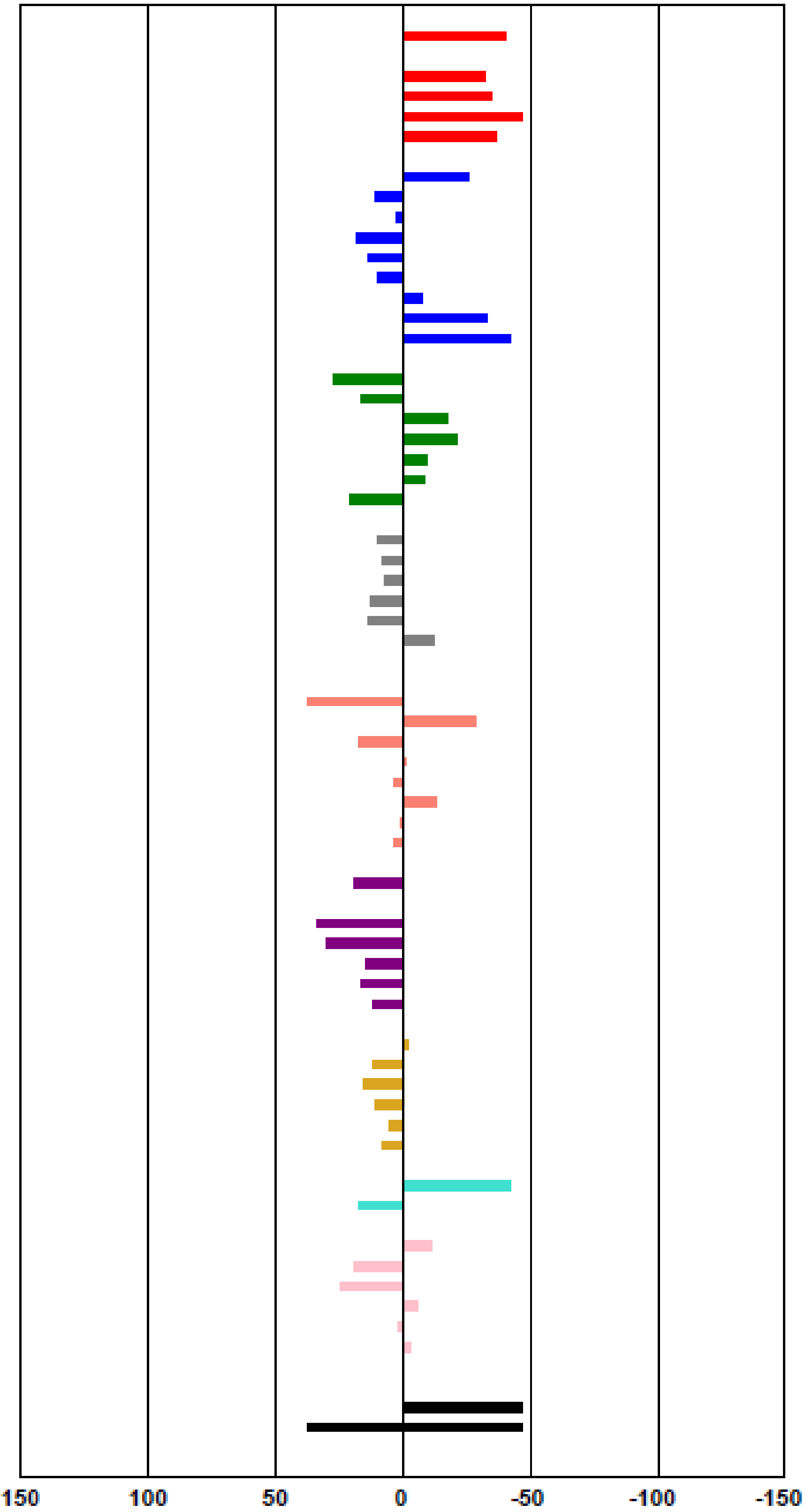

Developmental Therapeutics Program	NSC: D-795340 / 1	Conc: 1.00E-5 Molar	Test Date: Jan 17, 2017
One Dose Mean Graph	Experiment ID: 1701OS42		Report Date: Feb 07, 2017

Panel/Cell Line	Growth Percent
**Leukemia**	
CCRF-CEM	40.17
HL-60(TB)	56.64
K-562	32.92
MOLT-4	20.15
RPMI-8226	45.66
SR	46.37
**Non-Small Cell Lung Cancer**	
A549/ATCC	39.70
EKVX	77.65
HOP-62	50.13
HOP-92	149.34
NCI-H226	108.46
NCI-H23	78.15
NCI-H322M	82.58
NCI-H460	18.95
NCI-H522	59.63
**Colon Cancer**	
COLO 205	47.99
HCC-2998	73.75
HCT-116	26.08
HCT-15	29.91
HT29	35.16
KM12	49.53
SW-620	46.03
**CNS Cancer**	
SF-268	58.28
SF-295	49.55
SF-539	46.76
SNB-19	58.91
SNB-75	54.14
U251	35.20
**Melanoma**	
LOX IMVI	14.14
MALME-3M	107.18
M14	58.77
MDA-MB-435	59.79
SK-MEL-2	90.09
SK-MEL-28	62.41
SK-MEL-5	52.64
UACC-257	75.96
UACC-62	57.43
**Ovarian Cancer**	
IGROV1	60.30
OVCAR-3	54.00
OVCAR-4	75.25
OVCAR-5	91.81
OVCAR-8	41.66
NCI/ADR-RES	67.69
SK-OV-3	64.99
**Renal Cancer**	
786-0	60.57
A498	93.63
ACHN	52.67
SN12C	64.82
TK-10	69.41
UO-31	64.92
**Prostate Cancer**	
PC-3	49.83
DU-145	66.05
**Breast Cancer**	
MCF7	25.47
MDA-MB-231/ATCC	79.46
HS 578T	92.95
BT-549	67.19
T-47D	74.43
MDA-MB-468	77.77
Mean	60.19
Delta	46.05
Range	135.20

Mean Growth Percent - Growth Percent

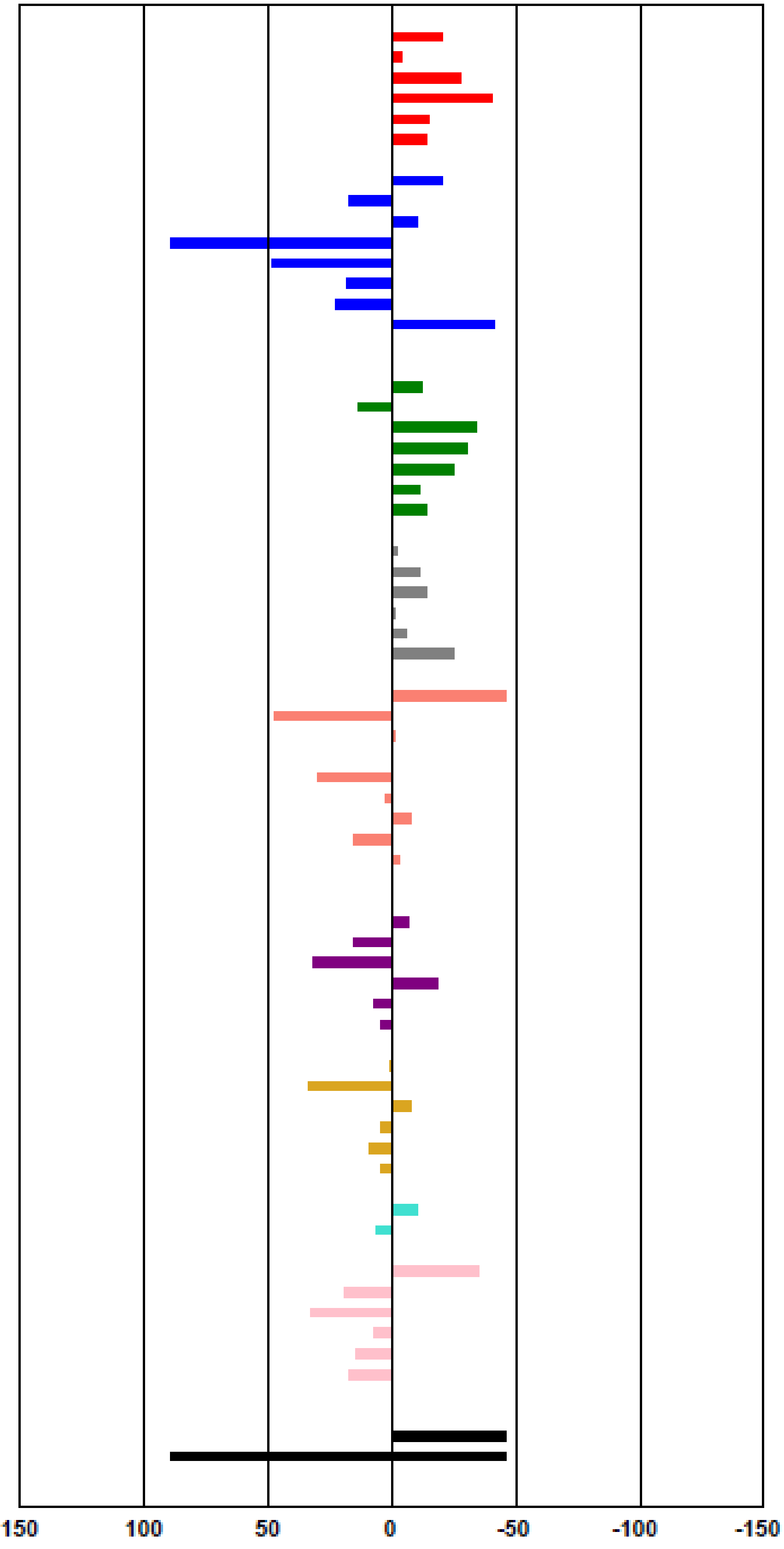

Supplement: Supplemental Material [file IENZ_A_1547286_SM0793.pdf]
