# Supplementary material for: Synthesis and in vitro anticancer activity of certain novel 1-(2-methyl-6-arylpyridin-3-yl)-3-phenylureas as apoptosis-inducing agents
Source: J Enzyme Inhib Med Chem. 2019 Feb 5;34(1):322–32. doi: 10.1080/14756366.2018.1547286 (PMC6366416; doi:10.1080/14756366.2018.1547286)
Supplement: Supplemental Material [file IENZ_A_1547286_SM0749.pdf]

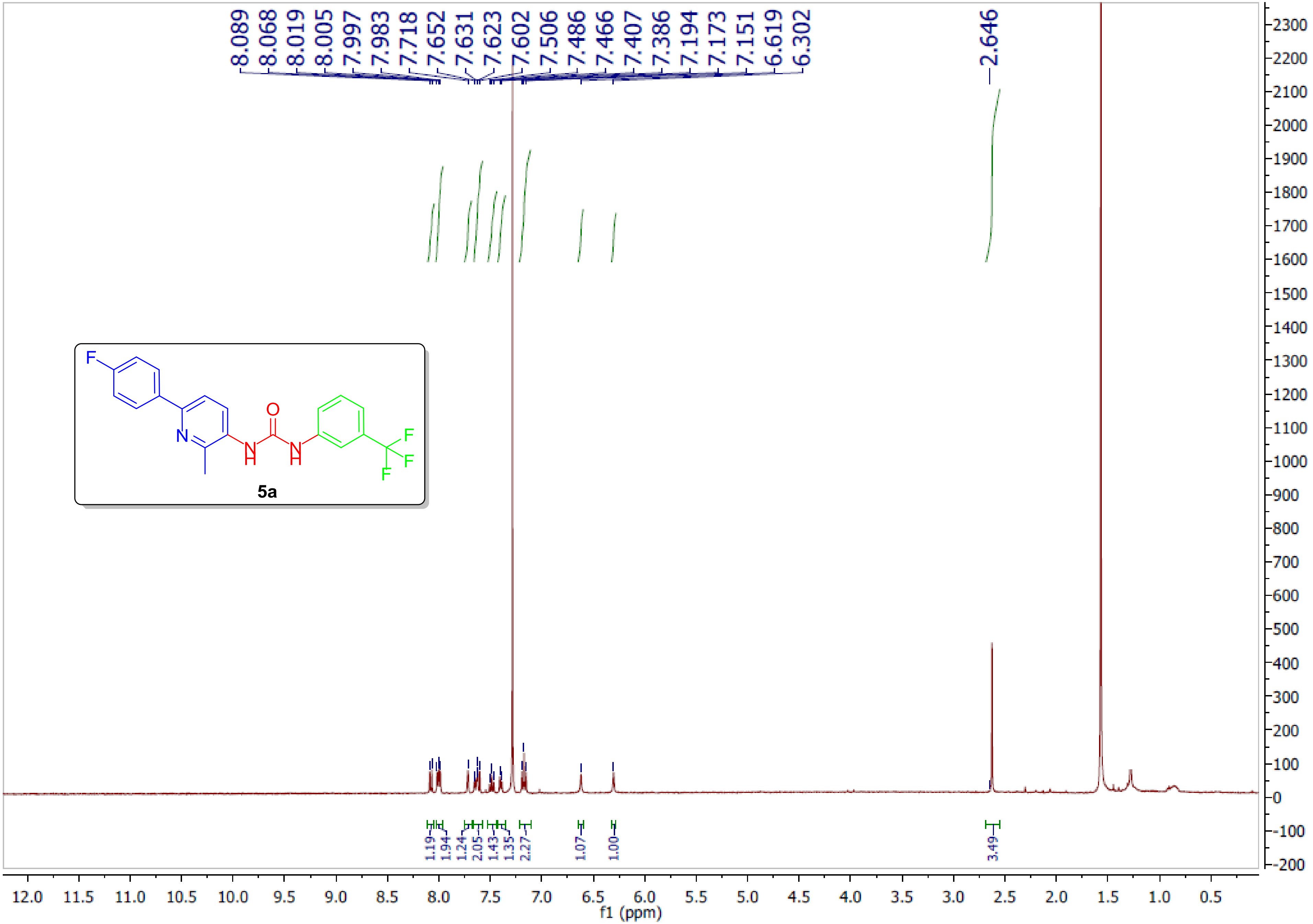
8.089
8.068
8.019
8.005
7.997
7.983
7.718
7.652
7.631
7.623
7.602
7.506
7.486
7.466
7.407
7.386
7.194
7.173
7.151
6.619
6.302
2.646
F
N
O
N
H
N
H
F
F
F
5a
1.19
1.94
1.24
2.05
1.43
1.35
2.27
1.07
1.00
3.49
12.0
11.5
11.0
10.5
10.0
9.5
9.0
8.5
8.0
7.5
7.0
6.5
6.0
5.5
5.0
4.5
4.0
3.5
3.0
2.5
2.0
1.5
1.0
0.5
f1 (ppm)
2300
2200
2100
2000
1900
1800
1700
1600
1500
1400
1300
1200
1100
1000
900
800
700
600
500
400
300
200
100
0
-100
-200

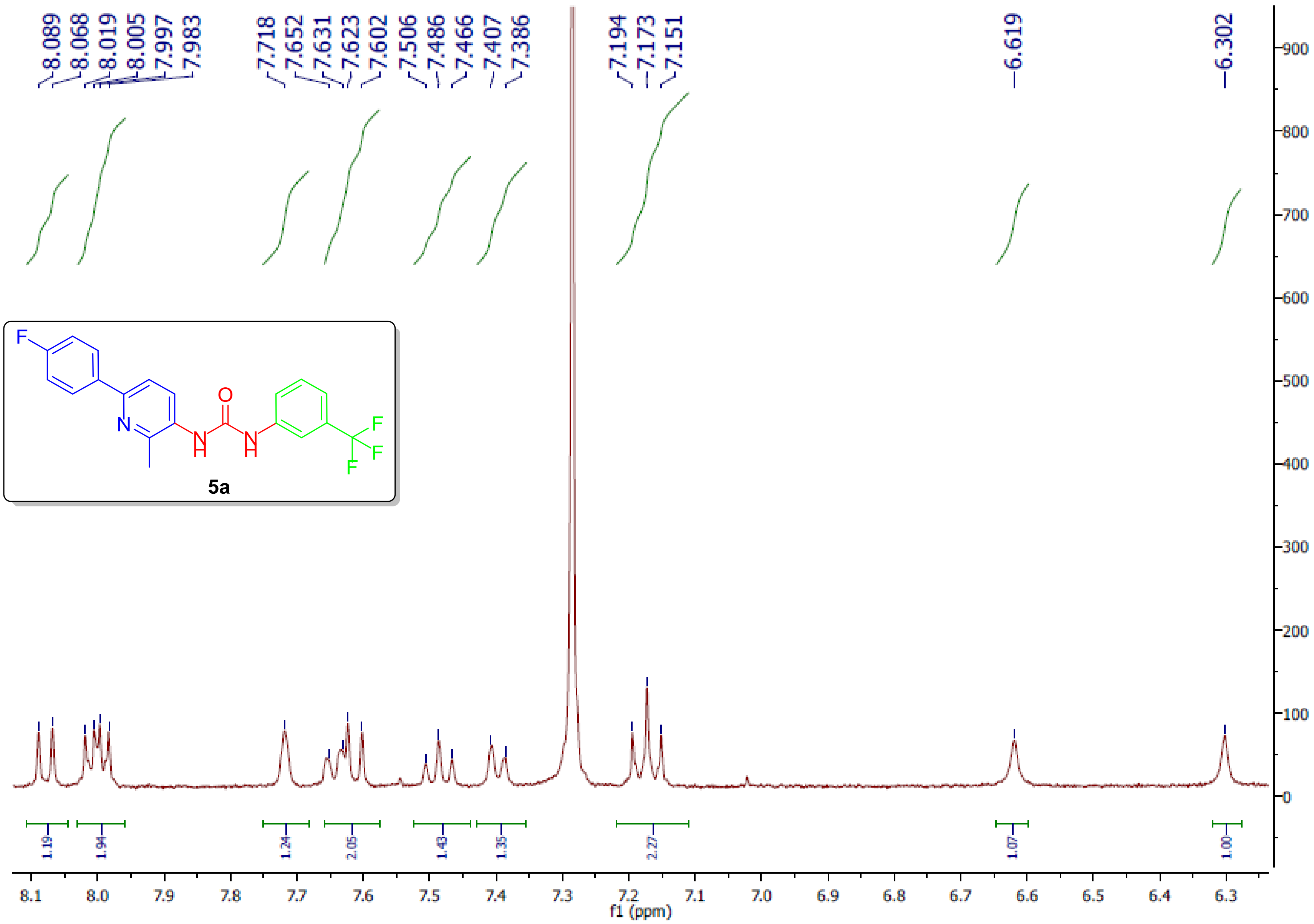
8.089
8.068
8.019
8.005
7.997
7.983
7.718
7.652
7.631
7.623
7.602
7.506
7.486
7.466
7.407
7.386
7.194
7.173
7.151
6.619
6.302
F
N
O
N
H
N
H
F
F
F
5a
1.19
1.94
1.24
2.05
1.43
1.35
2.27
1.07
1.00
8.1
8.0
7.9
7.8
7.7
7.6
7.5
7.4
7.3
7.2
7.1
7.0
6.9
6.8
6.7
6.6
6.5
6.4
6.3
f1 (ppm)
900
800
700
600
500
400
300
200
100
0

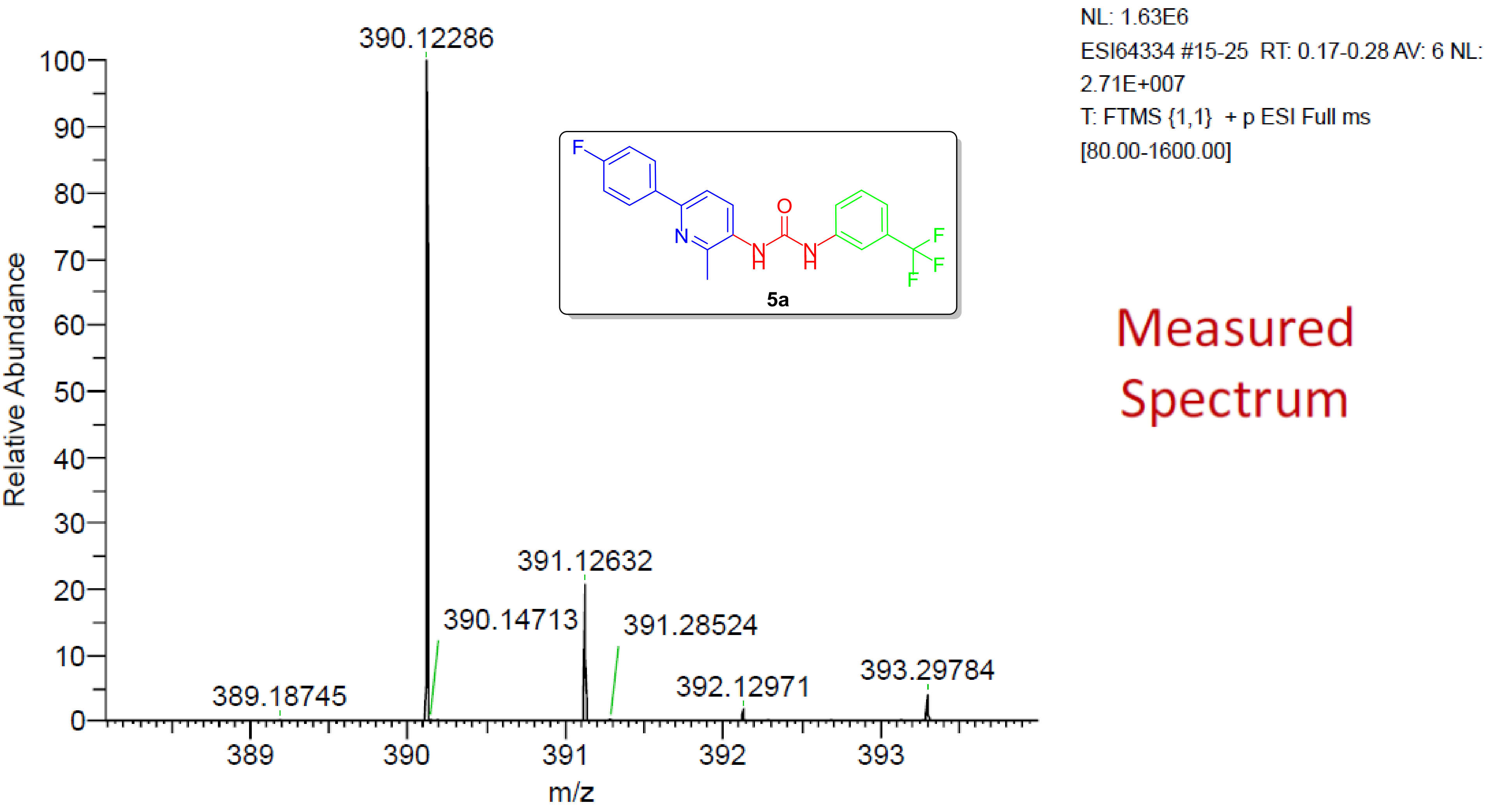

Measured Spectrum

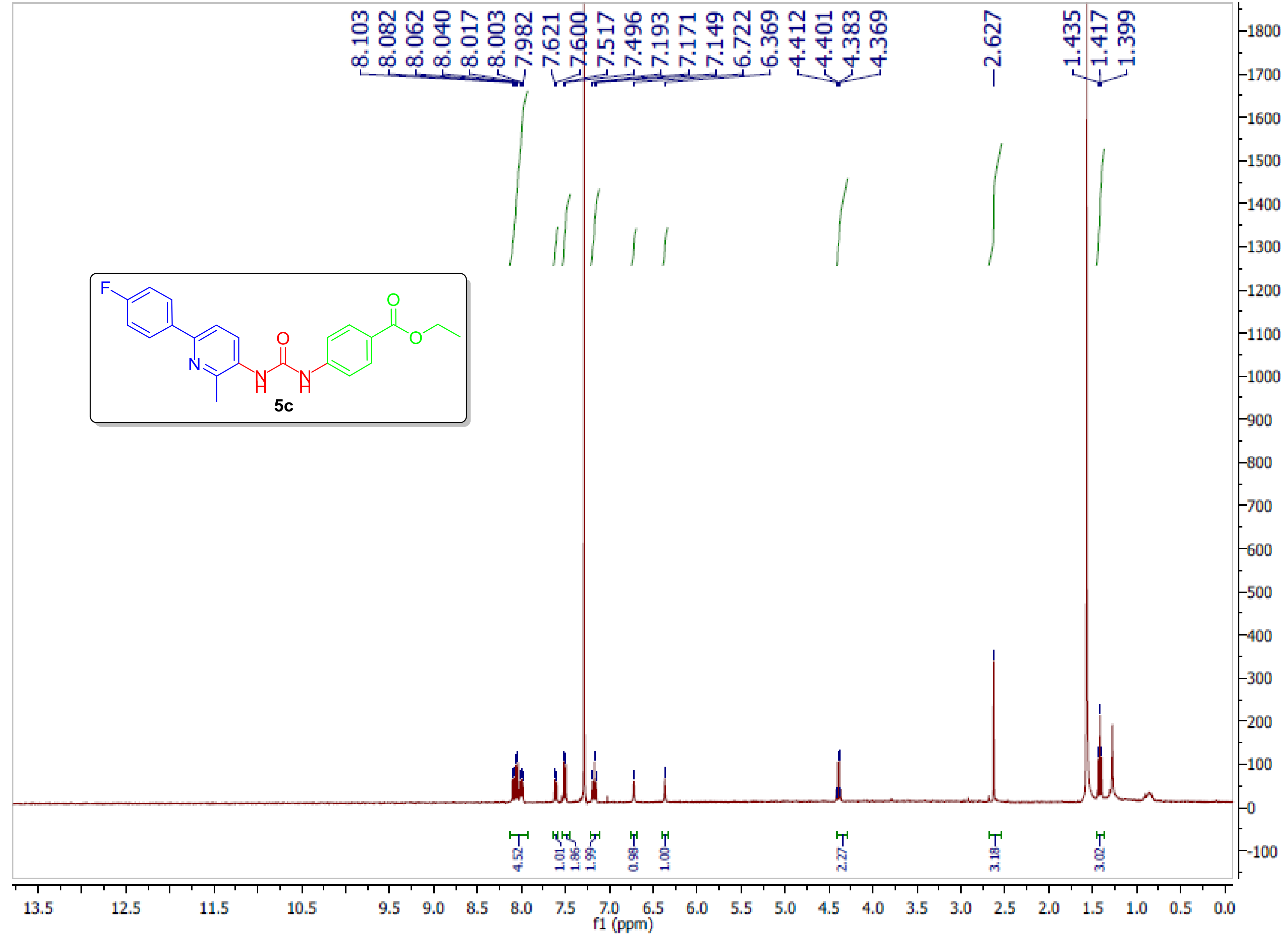
8.103
8.082
8.062
8.040
8.017
8.003
7.982
7.621
7.600
7.517
7.496
7.193
7.171
7.149
6.722
6.369
4.412
4.401
4.383
4.369
2.627
1.435
1.417
1.399
F
N
O
N
H
N
H
O
O
5c
4.52
1.01
1.86
1.99
0.98
1.00
2.27
3.18
3.02
f1 (ppm)

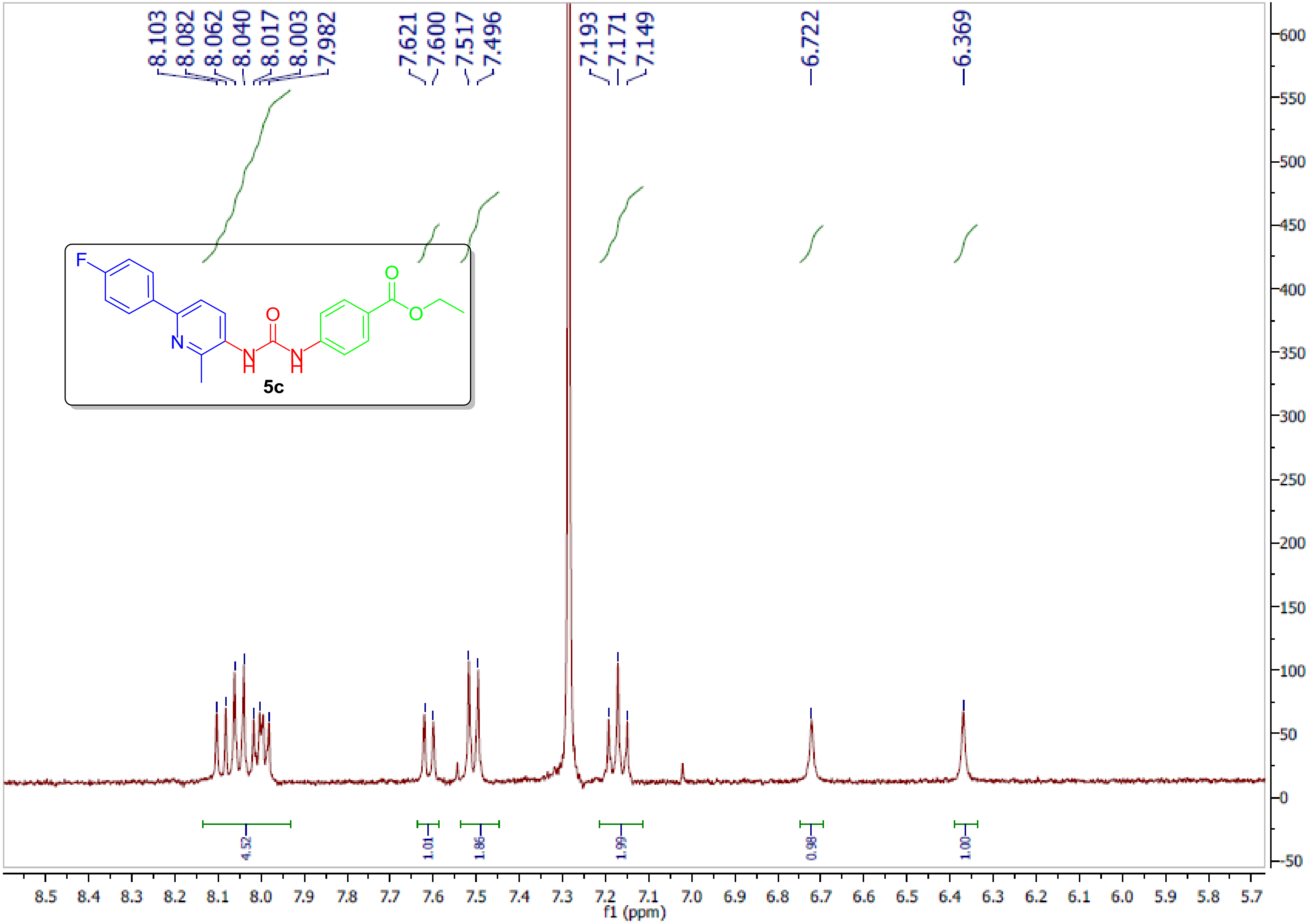

8.103
8.082
8.062
8.040
8.017
8.003
7.982
7.621
7.600
7.517
7.496
7.193
7.171
7.149
6.722
6.369
5c
4.52
1.01
1.86
1.99
0.98
1.00
f1 (ppm)

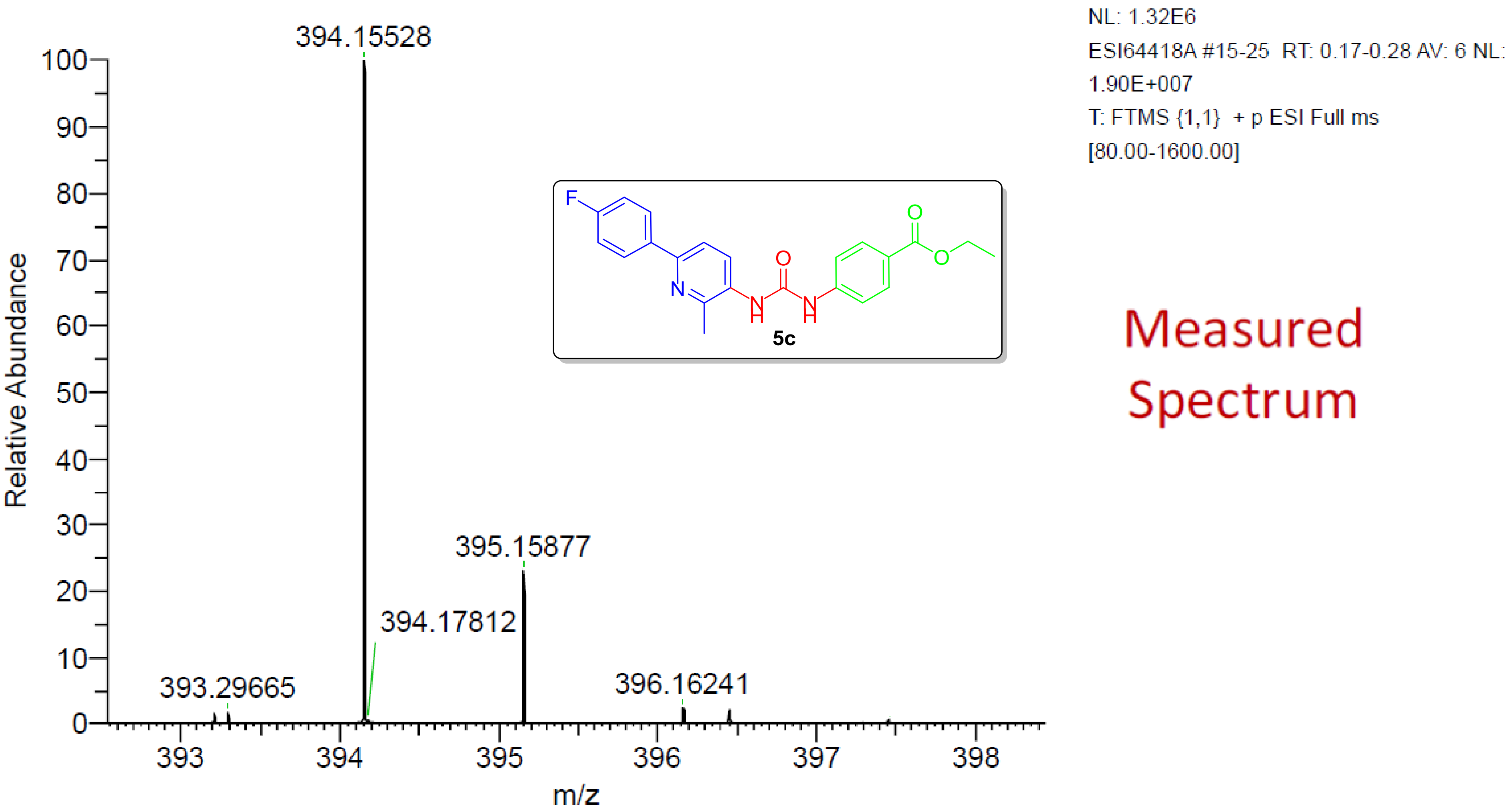
NL: 1.32E6
ESI64418A #15-25 RT: 0.17-0.28 AV: 6 NL:
1.90E+007
T: FTMS {1,1} + p ESI Full ms
[80.00-1600.00]
394.15528
395.15877
394.17812
393.29665
396.16241
Relative Abundance
m/z
F
N
O
N
H
N
H
O
O
5c
Measured
Spectrum

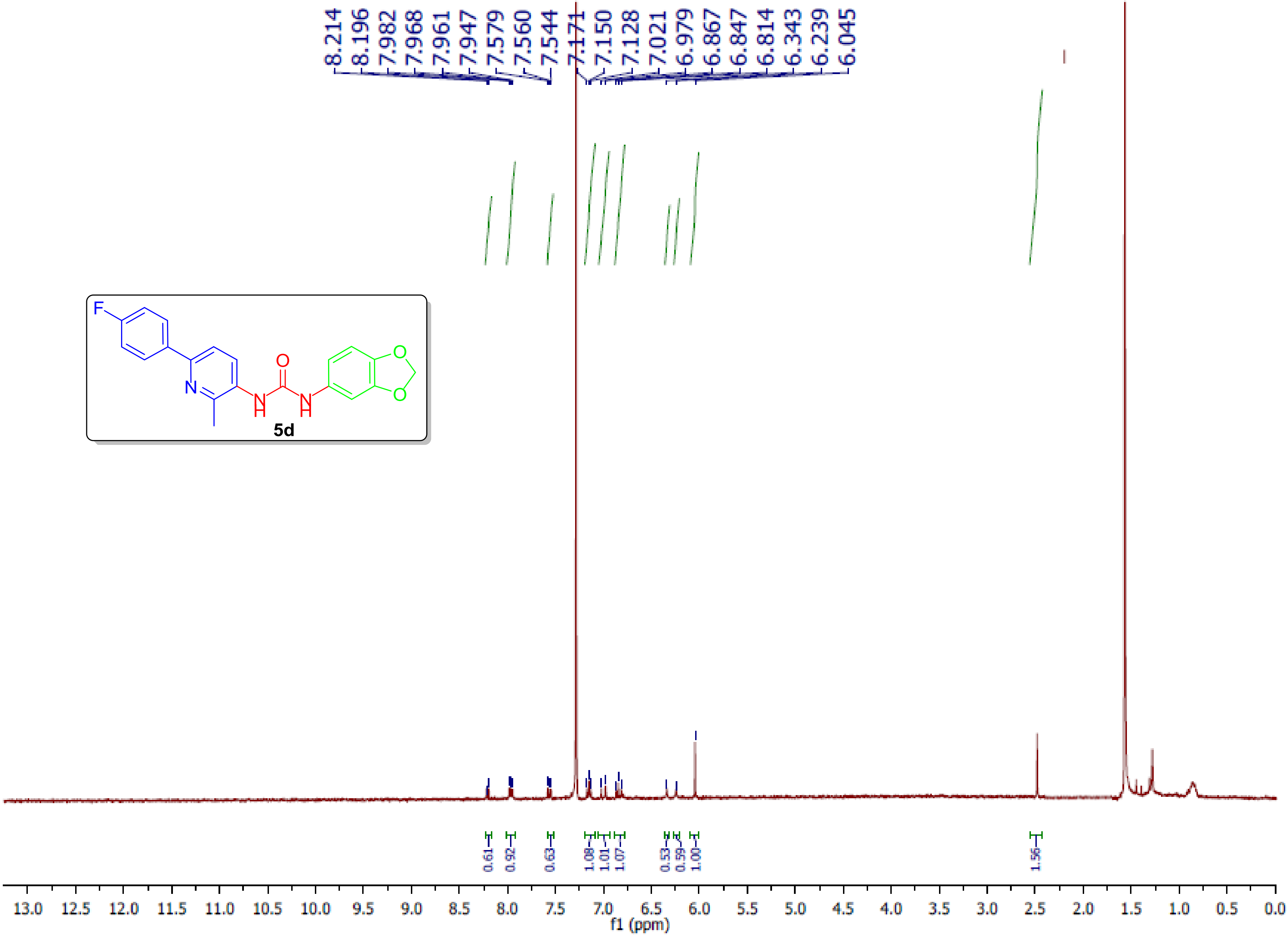
8.214
8.196
7.982
7.968
7.961
7.947
7.579
7.560
7.544
7.171
7.150
7.128
7.021
6.979
6.867
6.847
6.814
6.343
6.239
6.045
5d
F
N
O
N
H
0.61
0.92
0.63
1.08
1.01
1.07
0.53
0.59
1.00
1.56
13.0 12.5 12.0 11.5 11.0 10.5 10.0 9.5 9.0 8.5 8.0 7.5 7.0 6.5 6.0 5.5 5.0 4.5 4.0 3.5 3.0 2.5 2.0 1.5 1.0 0.5 0.0
f1 (ppm)

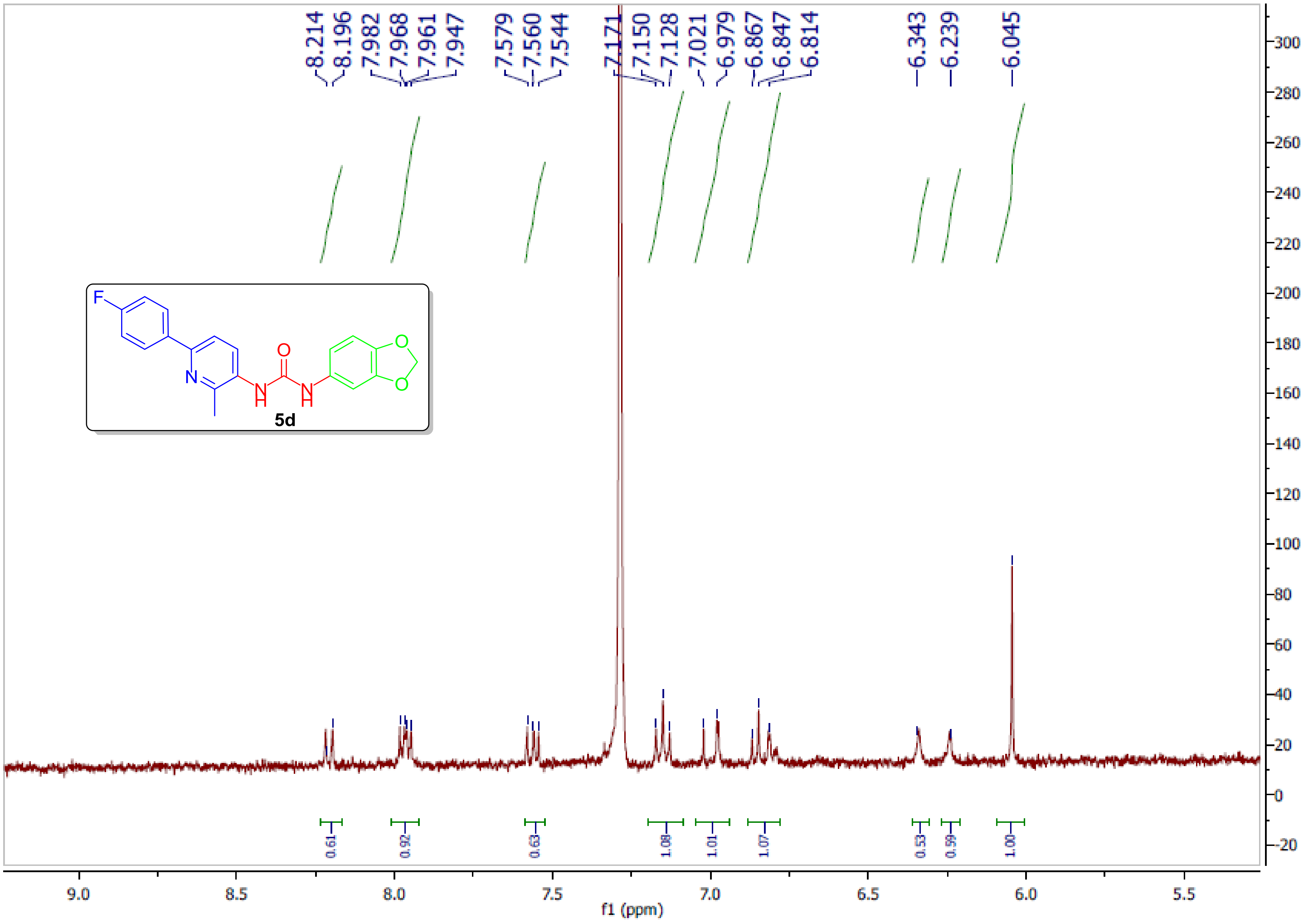
8.214
8.196
7.982
7.968
7.961
7.947
7.579
7.560
7.544
7.171
7.150
7.128
7.021
6.979
6.867
6.847
6.814
6.343
6.239
6.045
F
N
O
N
H
N
H
O
O
5d
0.61
0.92
0.63
1.08
1.01
1.07
0.53
0.59
1.00
9.0
8.5
8.0
7.5
7.0
6.5
6.0
5.5
f1 (ppm)

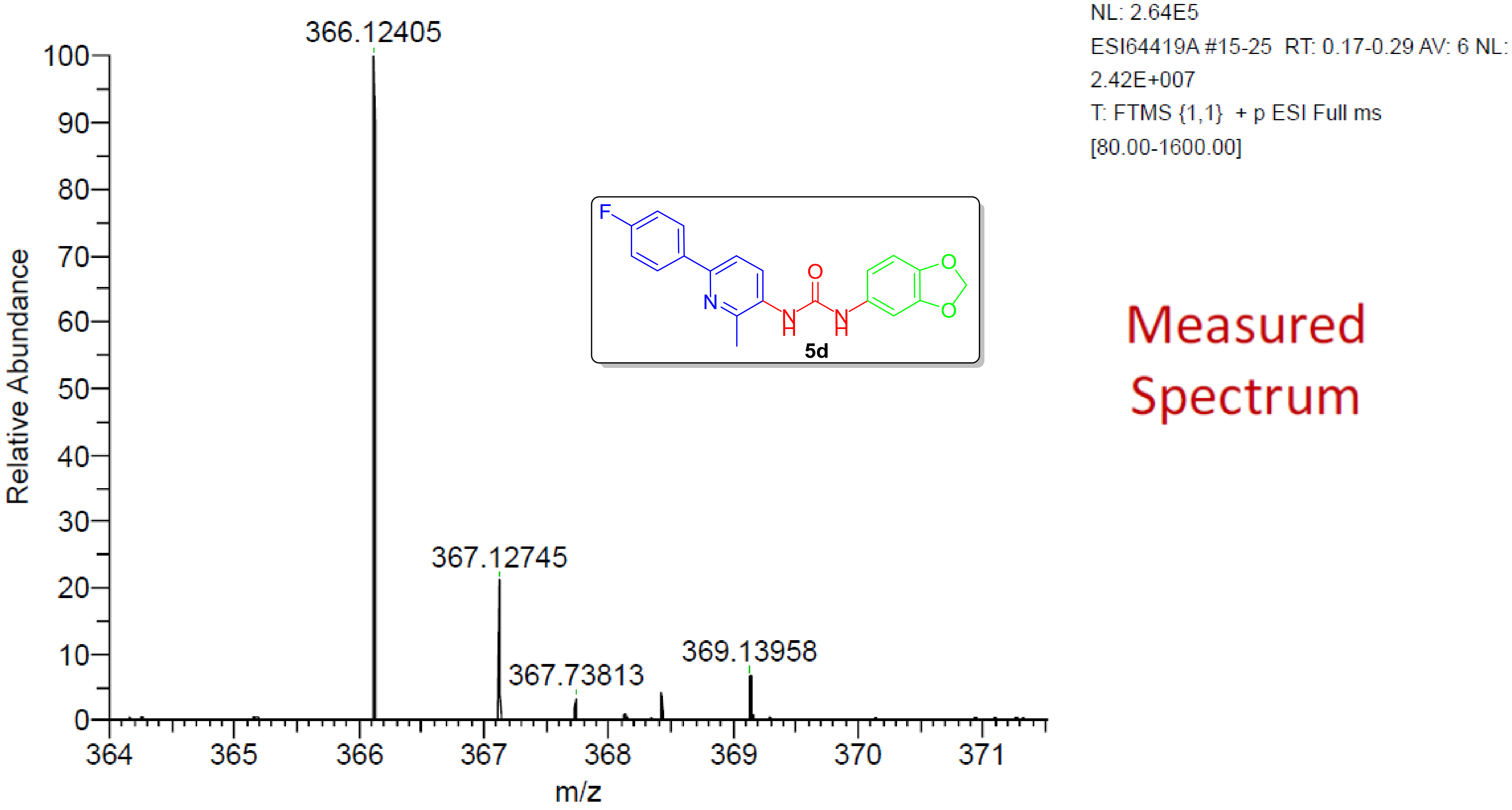
366.12405
367.12745
367.73813
369.13958
Relative Abundance
m/z
0
10
20
30
40
50
60
70
80
90
100
364
365
366
367
368
369
370
371
F
N
O
N
H
N
H
O
O
5d
NL: 2.64E5
ESI64419A #15-25 RT: 0.17-0.29 AV: 6 NL: 2.42E+007
T: FTMS {1,1} + p ESI Full ms [80.00-1600.00]
Measured Spectrum

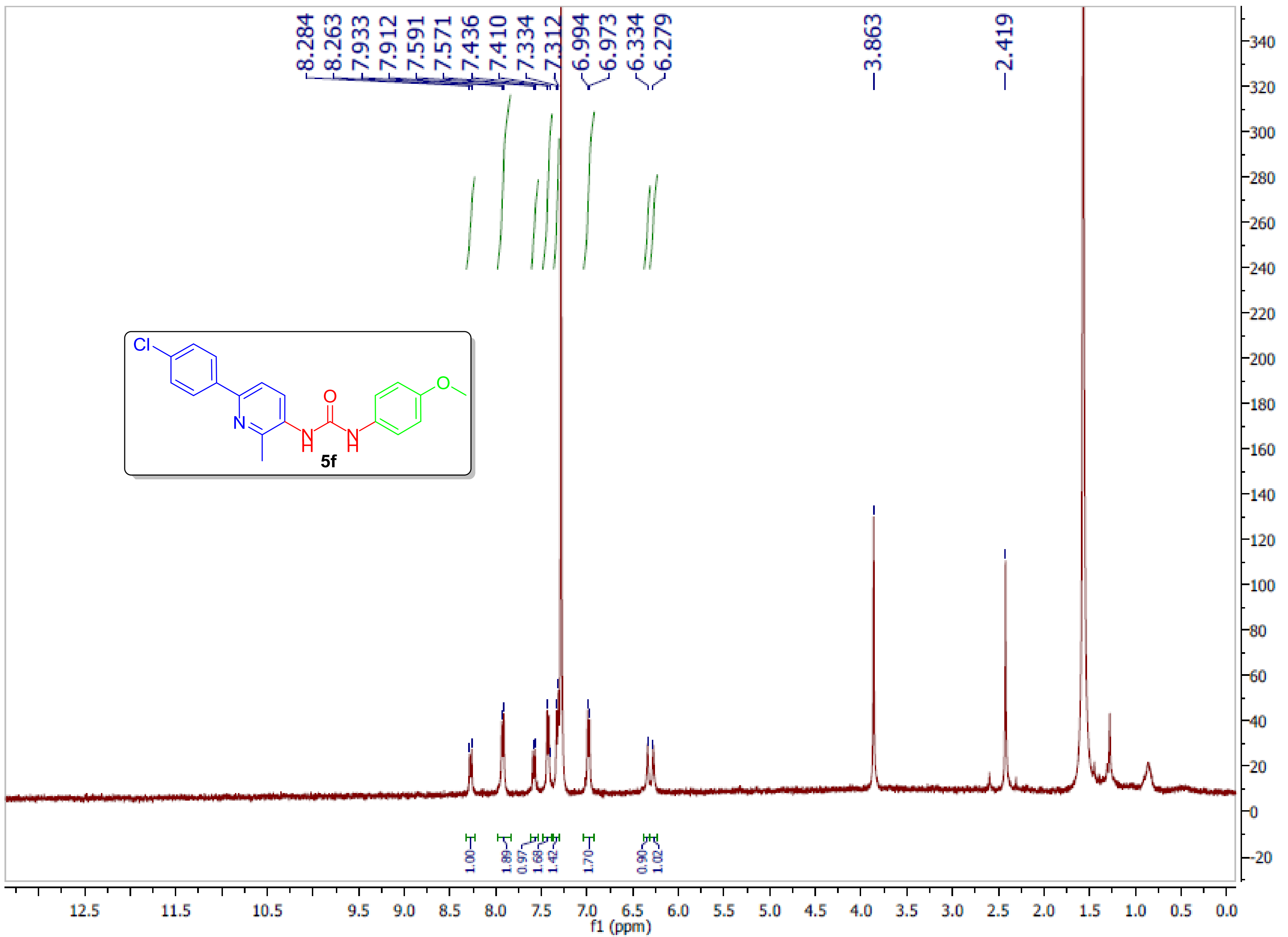

8.284
8.263
7.933
7.912
7.591
7.571
7.436
7.410
7.334
7.312
6.994
6.973
6.334
6.279
3.863
2.419
Cl
N
O
N
H
N
H
O
5f
1.00
1.89
0.97
1.68
1.42
1.70
0.90
1.02
f1 (ppm)

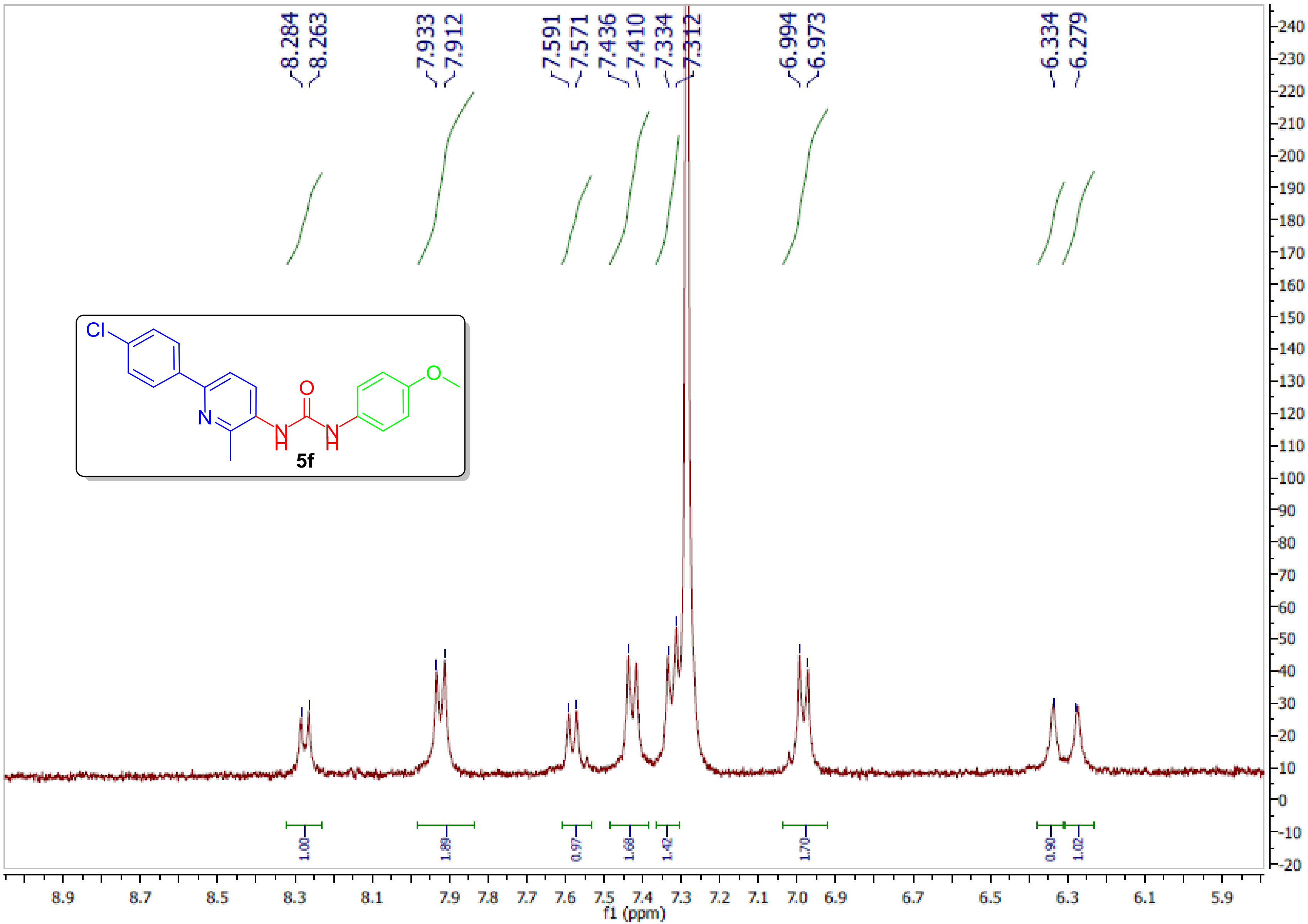

8.284
8.263
7.933
7.912
7.591
7.571
7.436
7.410
7.334
7.312
6.994
6.973
6.334
6.279
1.00
1.89
0.97
1.68
1.42
1.70
0.90
1.02
Cl
N
O
N
H
N
H
O
5f
f1 (ppm)

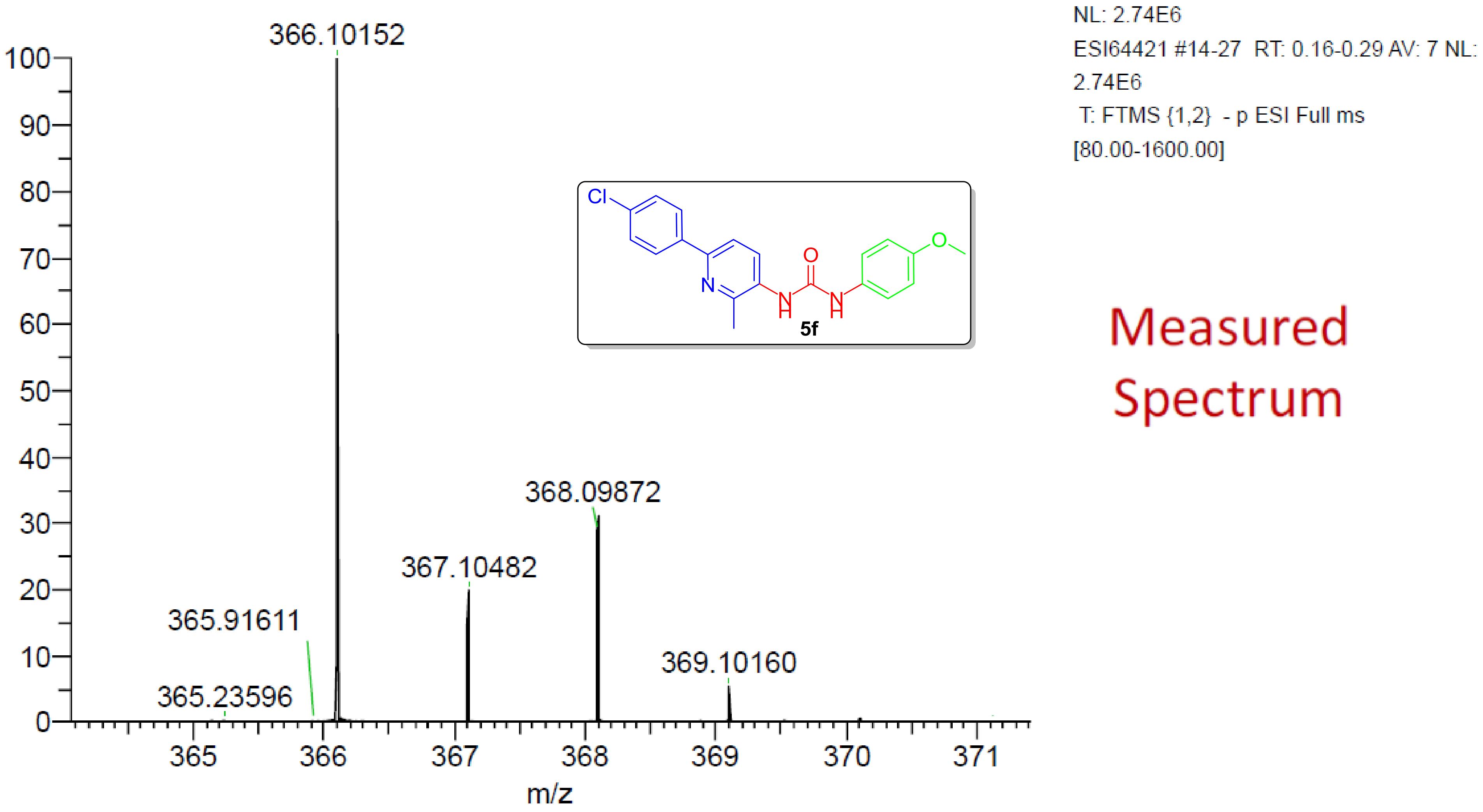

NL: 2.74E6
ESI64421 #14-27 RT: 0.16-0.29 AV: 7 NL: 2.74E6
T: FTMS {1,2} - p ESI Full ms [80.00-1600.00]
366.10152
368.09872
367.10482
365.91611
365.23596
369.10160
m/z
Cl
N
O
N
H
N
H
O
5f
Measured Spectrum

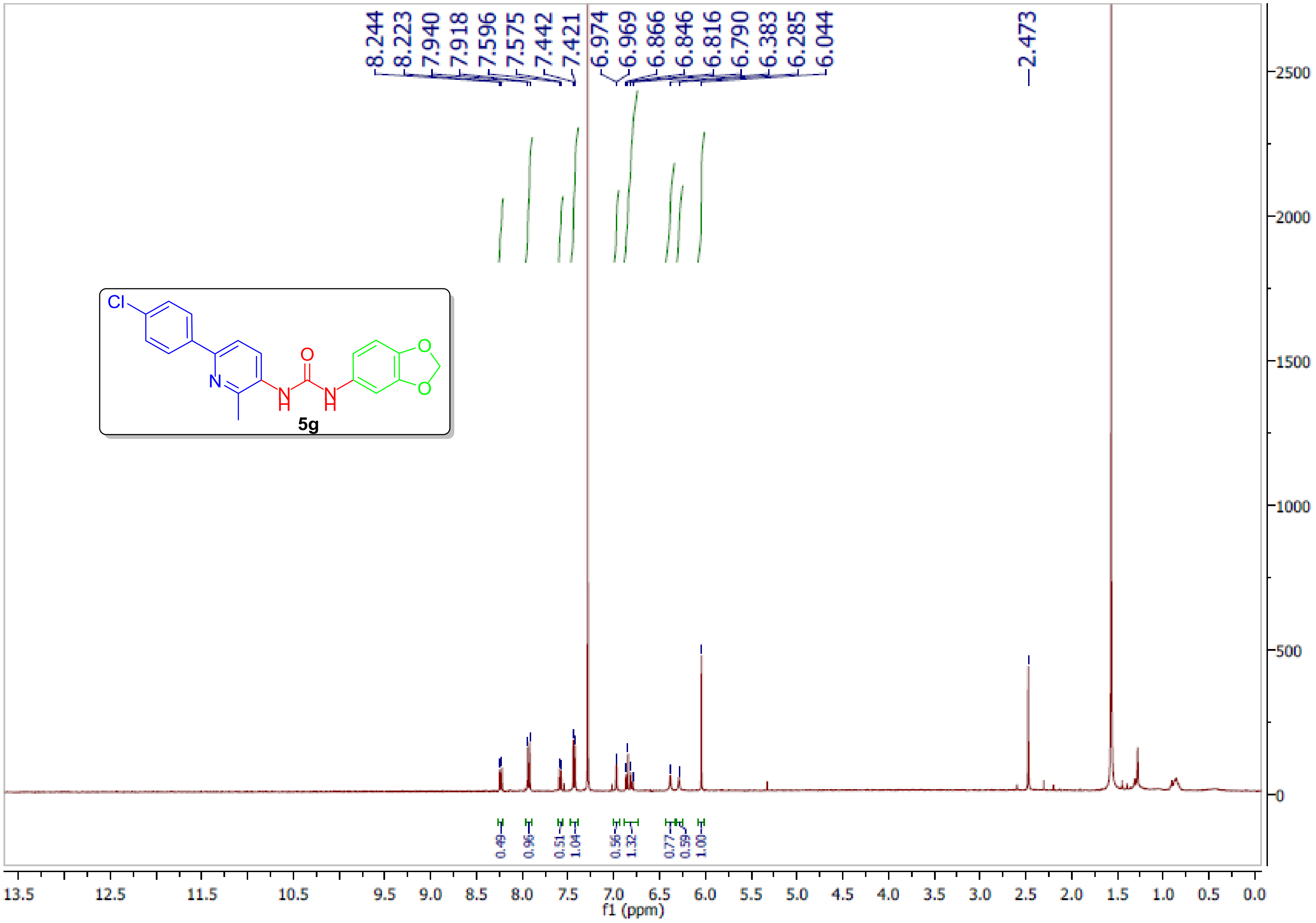
8.244
8.223
7.940
7.918
7.596
7.575
7.442
7.421
6.974
6.969
6.866
6.846
6.816
6.790
6.383
6.285
6.044
2.473
Cl
N
O
N
H
N
H
O
O
5g
0.49
0.96
0.51
1.04
0.56
1.32
0.77
0.59
1.00
2500
2000
1500
1000
500
0
13.5
12.5
11.5
10.5
9.5
9.0
8.5
8.0
7.5
7.0
6.5
6.0
5.5
5.0
4.5
4.0
3.5
3.0
2.5
2.0
1.5
1.0
0.5
0.0
f1 (ppm)

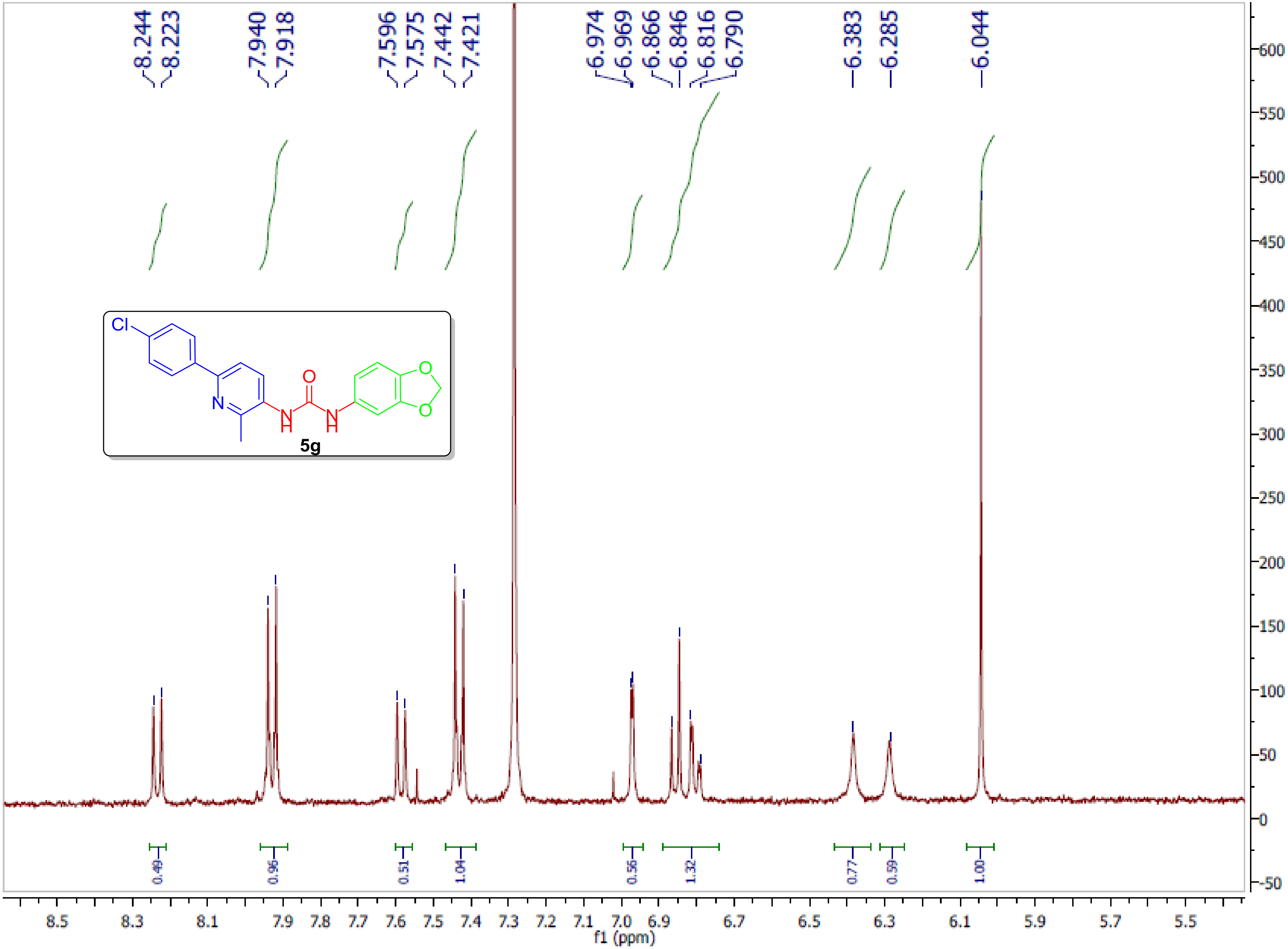

8.244
8.223
7.940
7.918
7.596
7.575
7.442
7.421
6.974
6.969
6.866
6.846
6.816
6.790
6.383
6.285
6.044
Cl
N
O
N
H
N
H
O
O
5g
0.49
0.96
0.51
1.04
0.56
1.32
0.77
0.59
1.00
f1 (ppm)

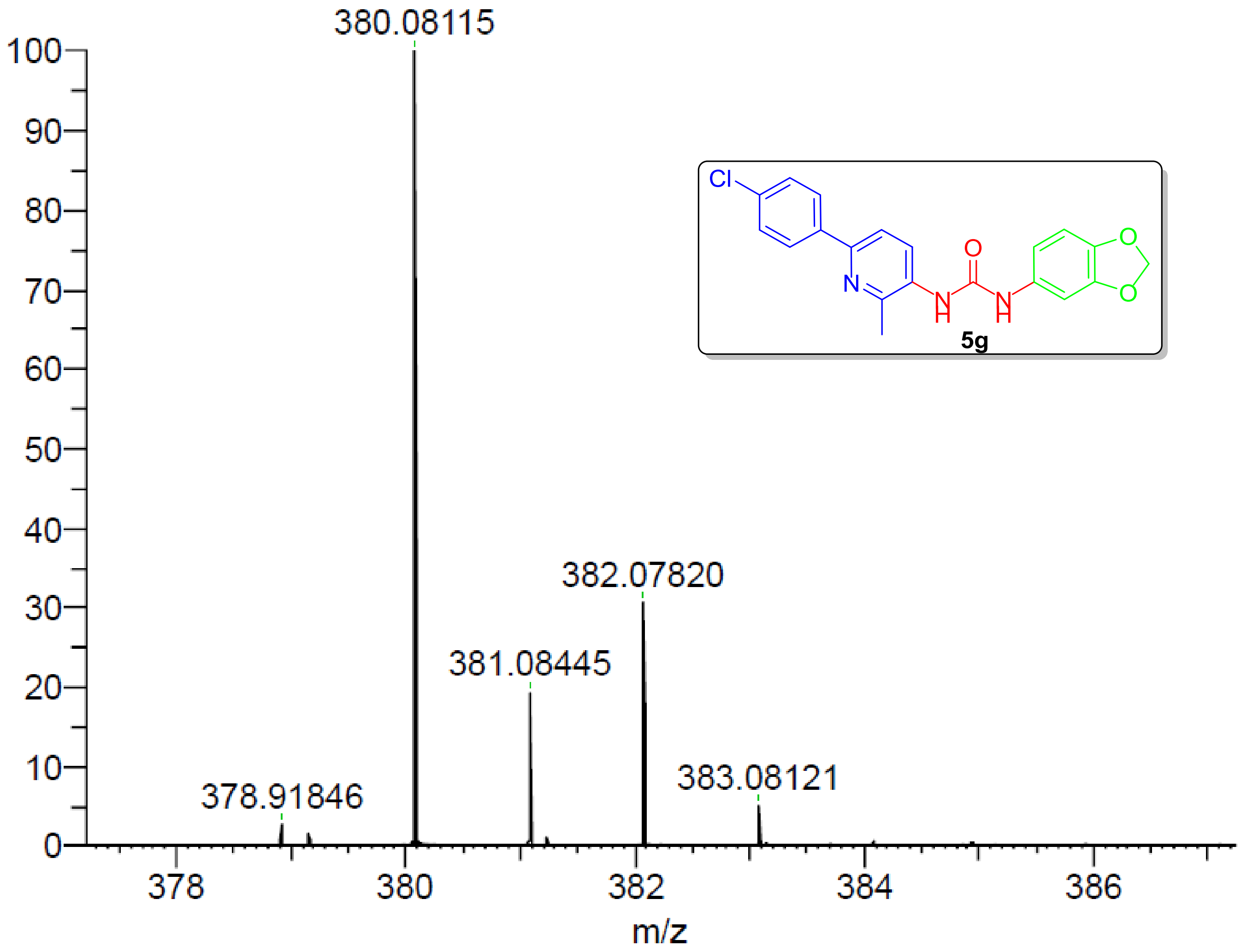

NL: 9.32E5
ESI64422 #13-27 RT: 0.16-0.3 AV: 7 NL: 9.32E5
T: FTMS {1,2} - p ESI Full ms [80.00-1600.00]

Measured Spectrum

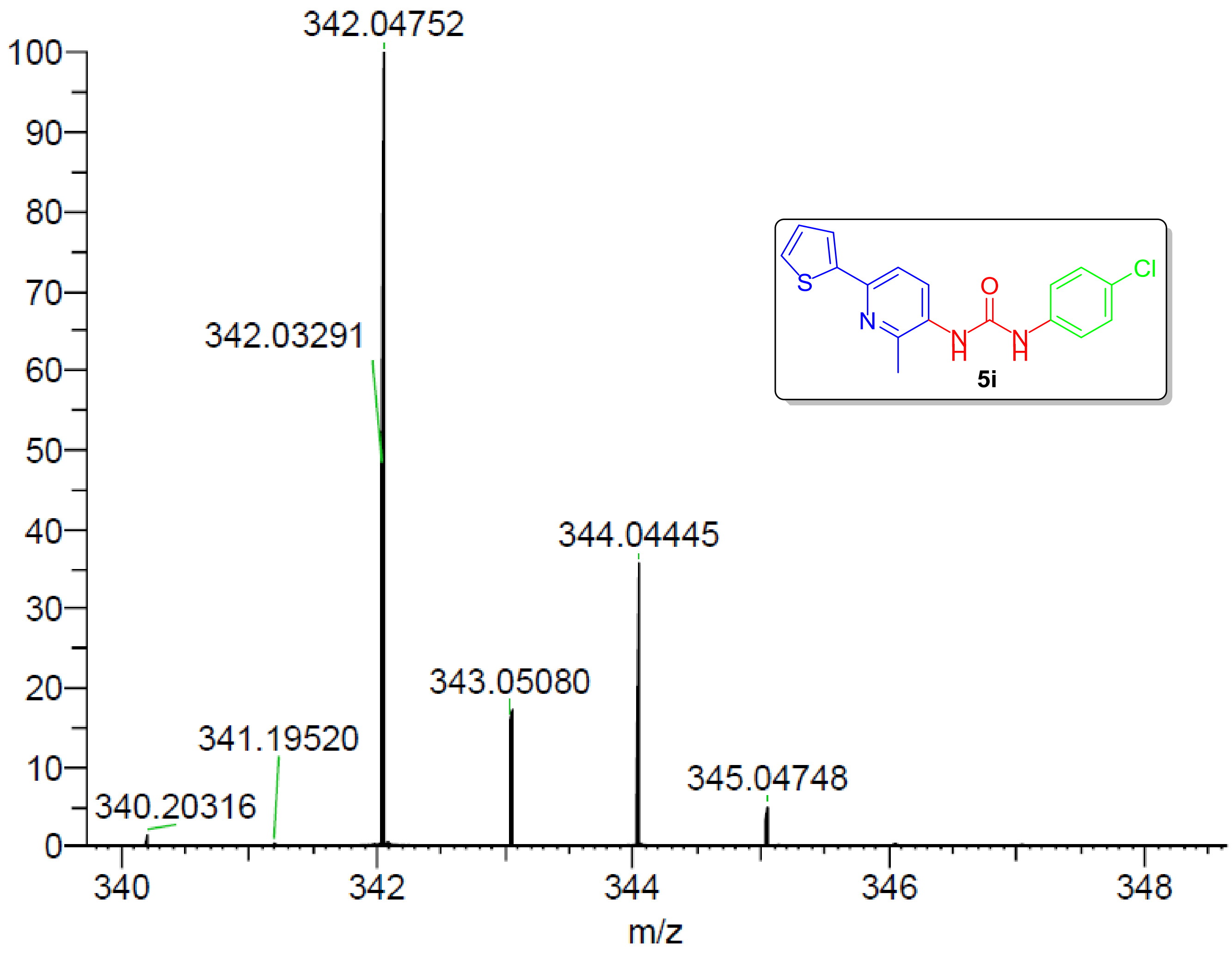

NL: 1.72E6
ESI64426 #11-46 RT: 0.13-0.53 AV: 18 NL: 1.72E6
T: FTMS {1,2} - p ESI Full ms [80.00-1600.00]

Measured Spectrum

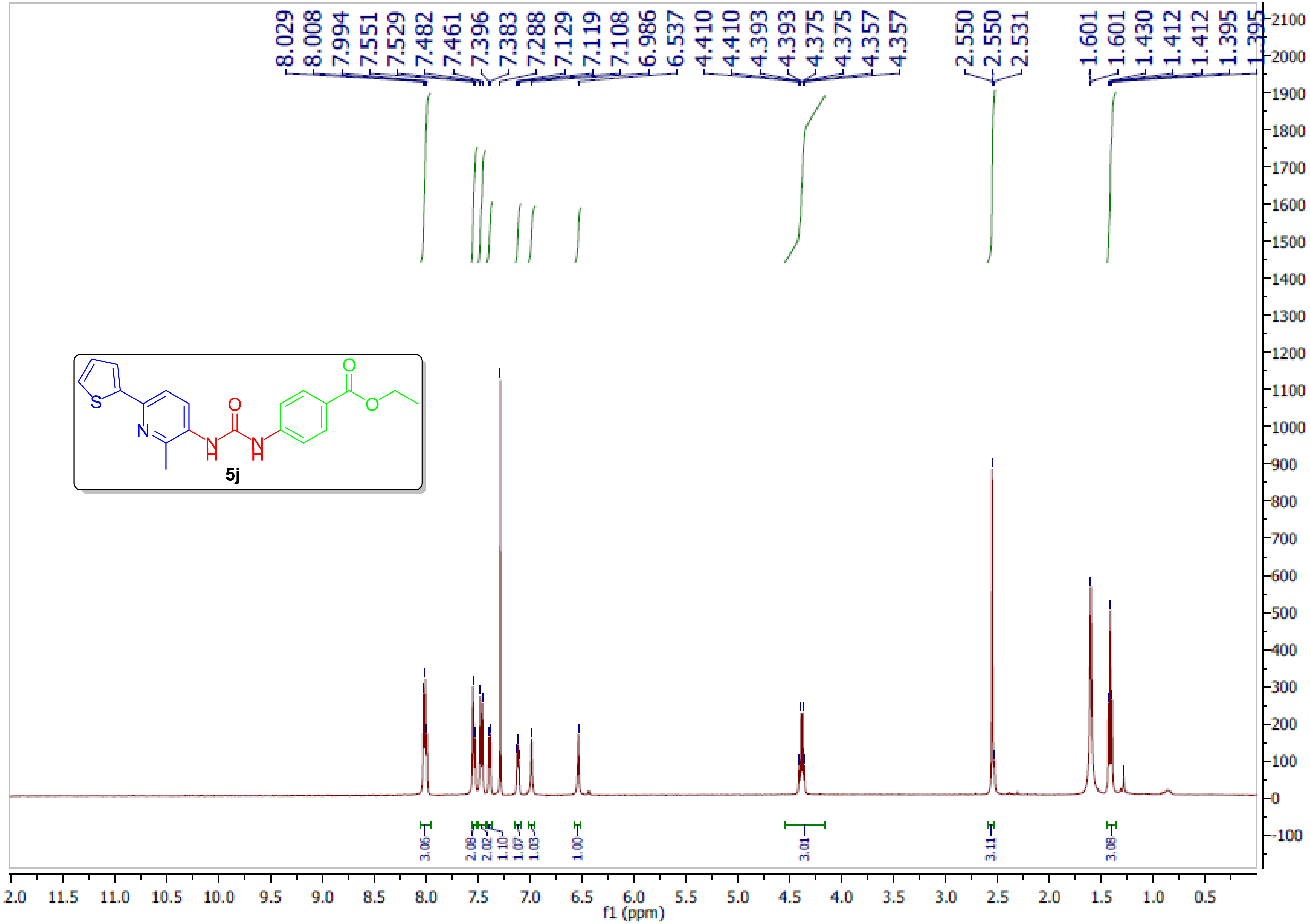
8.029
8.008
7.994
7.551
7.529
7.482
7.461
7.396
7.383
7.288
7.129
7.119
7.108
6.986
6.537
4.410
4.410
4.393
4.393
4.375
4.375
4.357
4.357
2.550
2.550
2.531
1.601
1.601
1.430
1.412
1.412
1.395
1.395
5j
3.06
2.08
2.02
1.10
1.07
1.03
1.00
3.01
3.11
3.08
f1 (ppm)

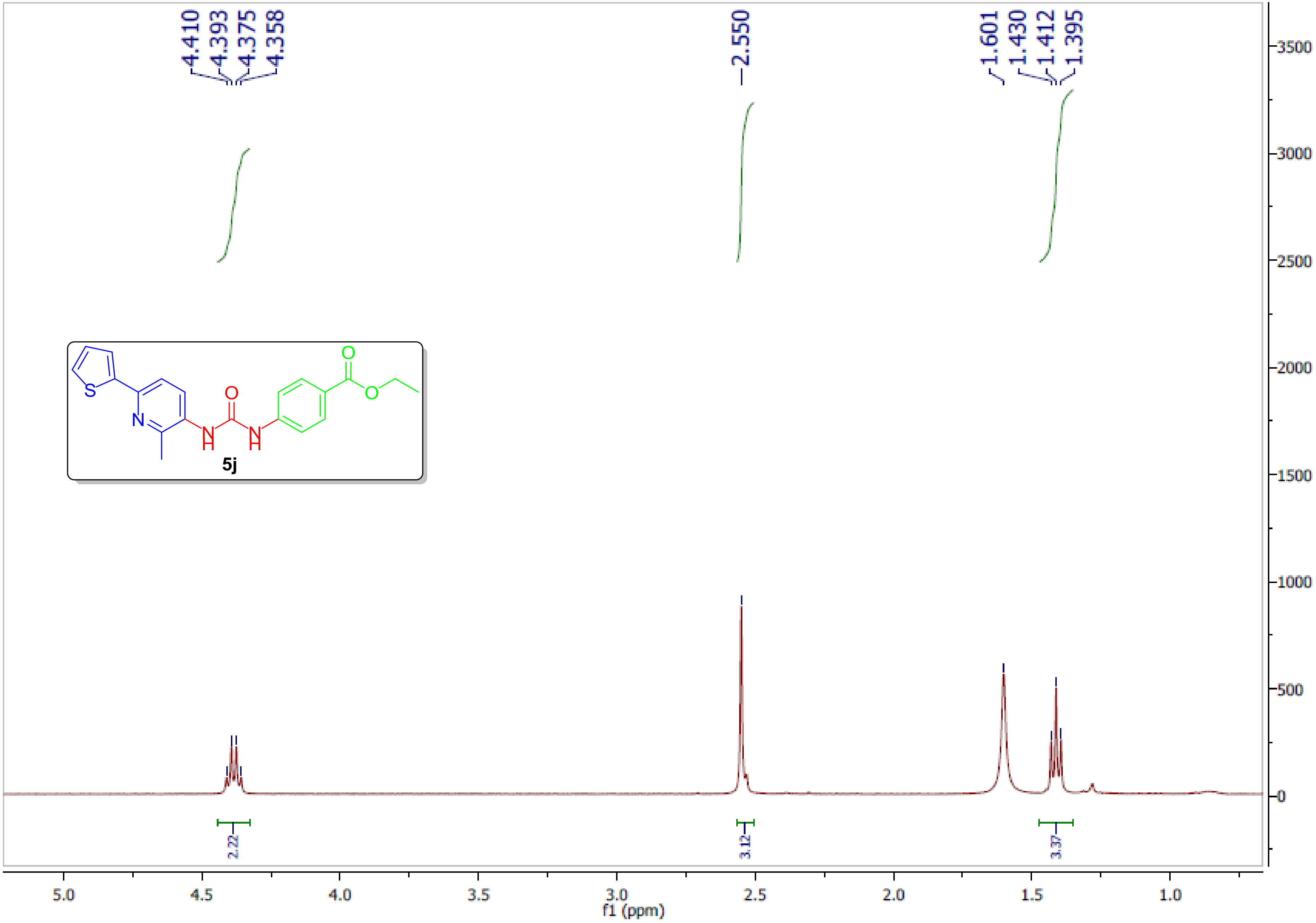
4.410
4.393
4.375
4.358
2.550
1.601
1.430
1.412
1.395
5j
2.22
3.12
3.37
5.0
4.5
4.0
3.5
3.0
2.5
2.0
1.5
1.0
f1 (ppm)
3500
3000
2500
2000
1500
1000
500
0

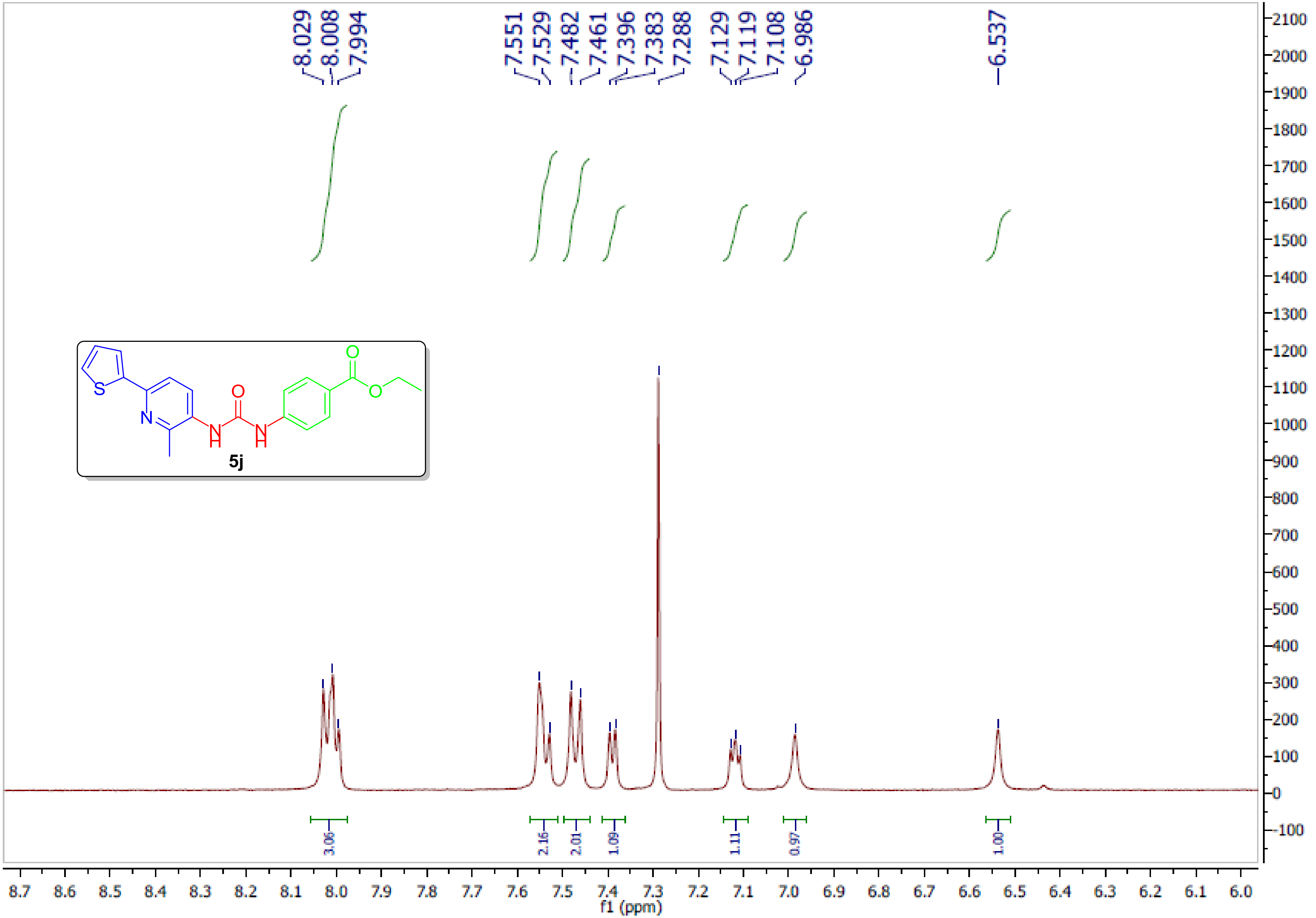
8.029
8.008
7.994
7.551
7.529
7.482
7.461
7.396
7.383
7.288
7.129
7.119
7.108
6.986
6.537
3.06
2.16
2.01
1.09
1.11
0.97
1.00
5j
f1 (ppm)

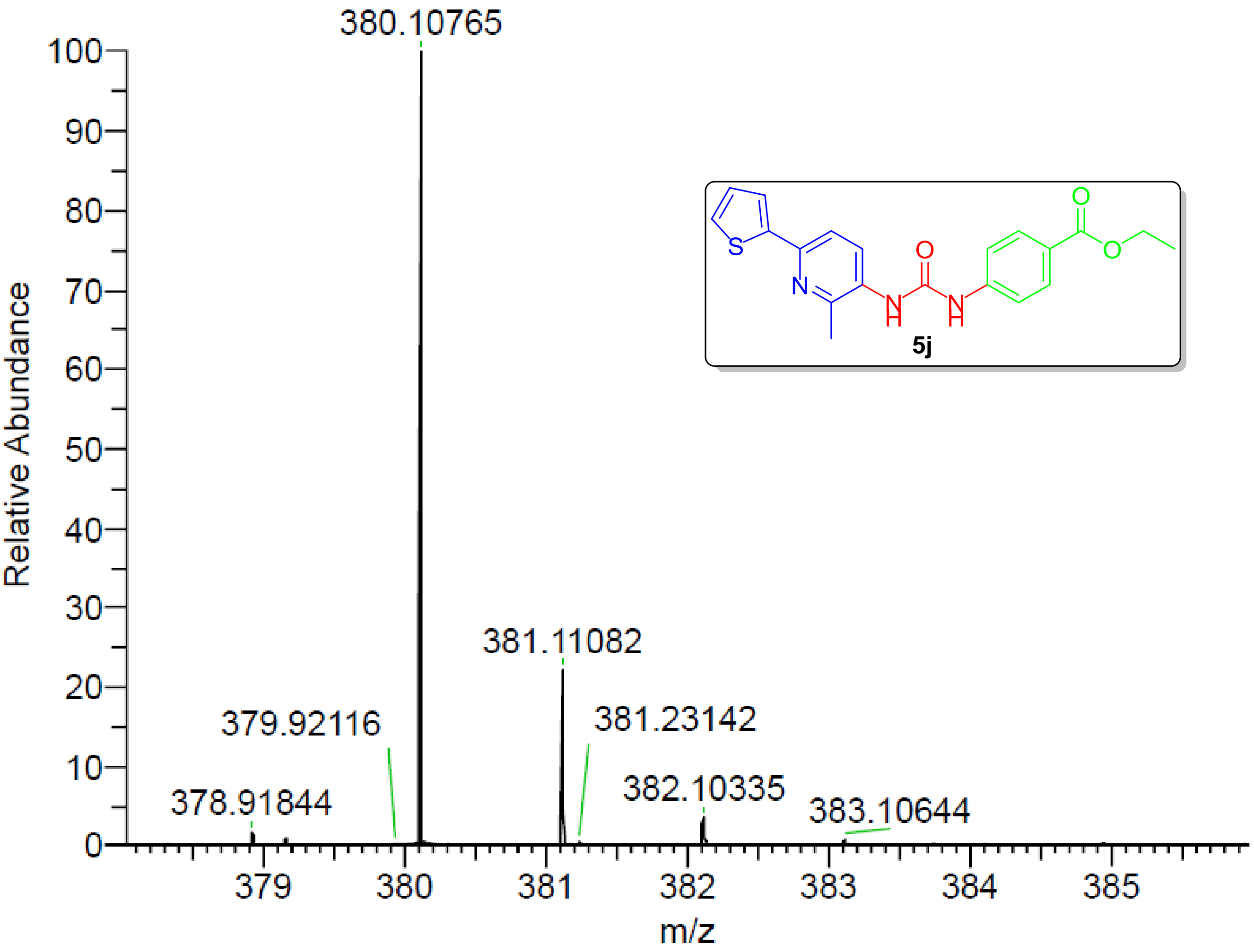

NL: 2.37E6
ESI64424 #11-37 RT: 0.13-0.41 AV: 13 NL: 2.37E+006
T: FTMS {1,2} - p ESI Full ms [80.00-1600.00]

Measured Spectrum

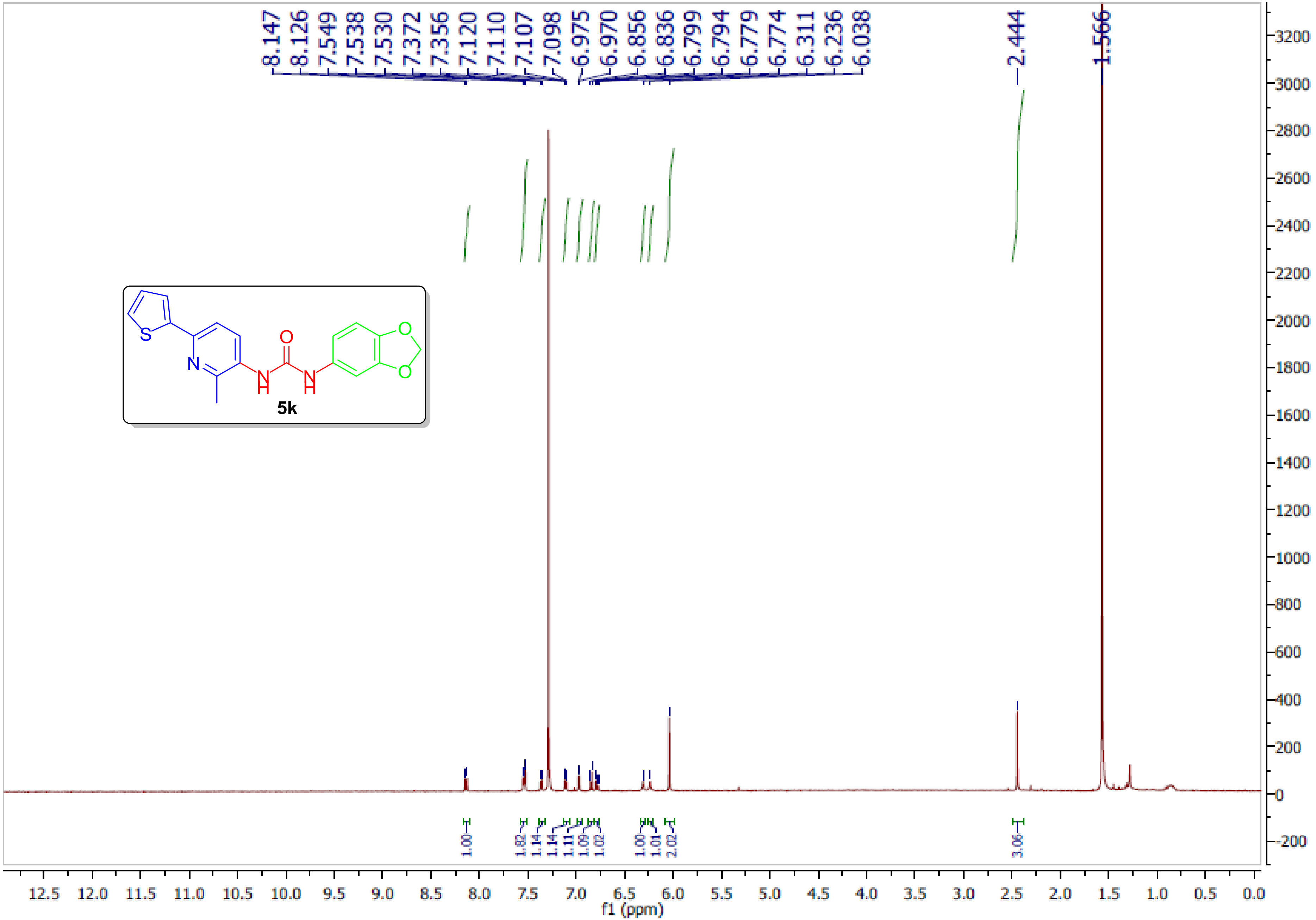
8.147
8.126
7.549
7.538
7.530
7.372
7.356
7.120
7.110
7.107
7.098
6.975
6.970
6.856
6.836
6.799
6.794
6.779
6.774
6.311
6.236
6.038
2.444
1.566
5k
1.00
1.82
1.14
1.14
1.11
1.09
1.02
1.00
1.01
2.02
3.06
f1 (ppm)

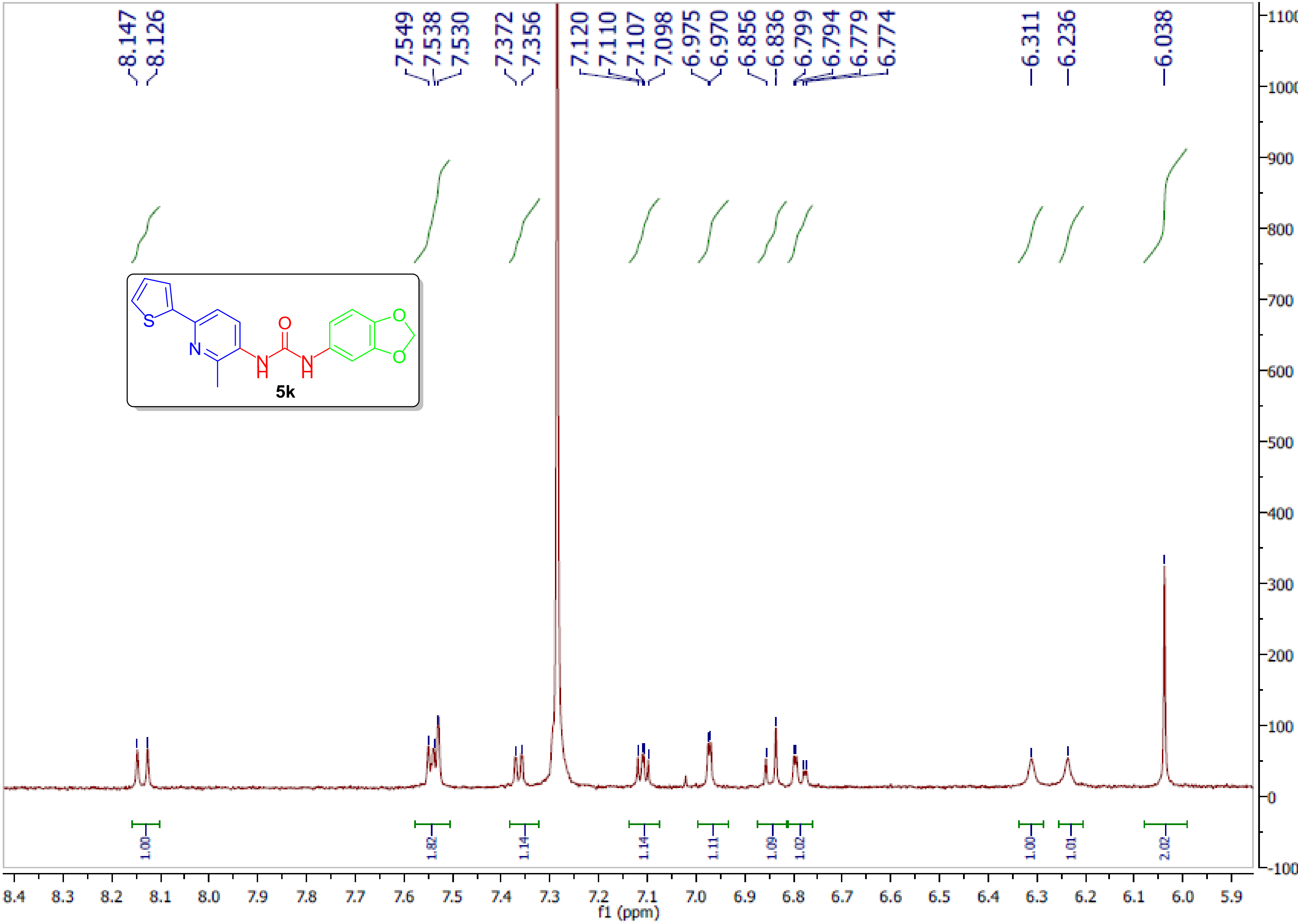

8.147
8.126
7.549
7.538
7.530
7.372
7.356
7.120
7.110
7.107
7.098
6.975
6.970
6.856
6.836
6.799
6.794
6.779
6.774
6.311
6.236
6.038
1.00
1.82
1.14
1.14
1.11
1.09
1.02
1.00
1.01
2.02
5k
f1 (ppm)

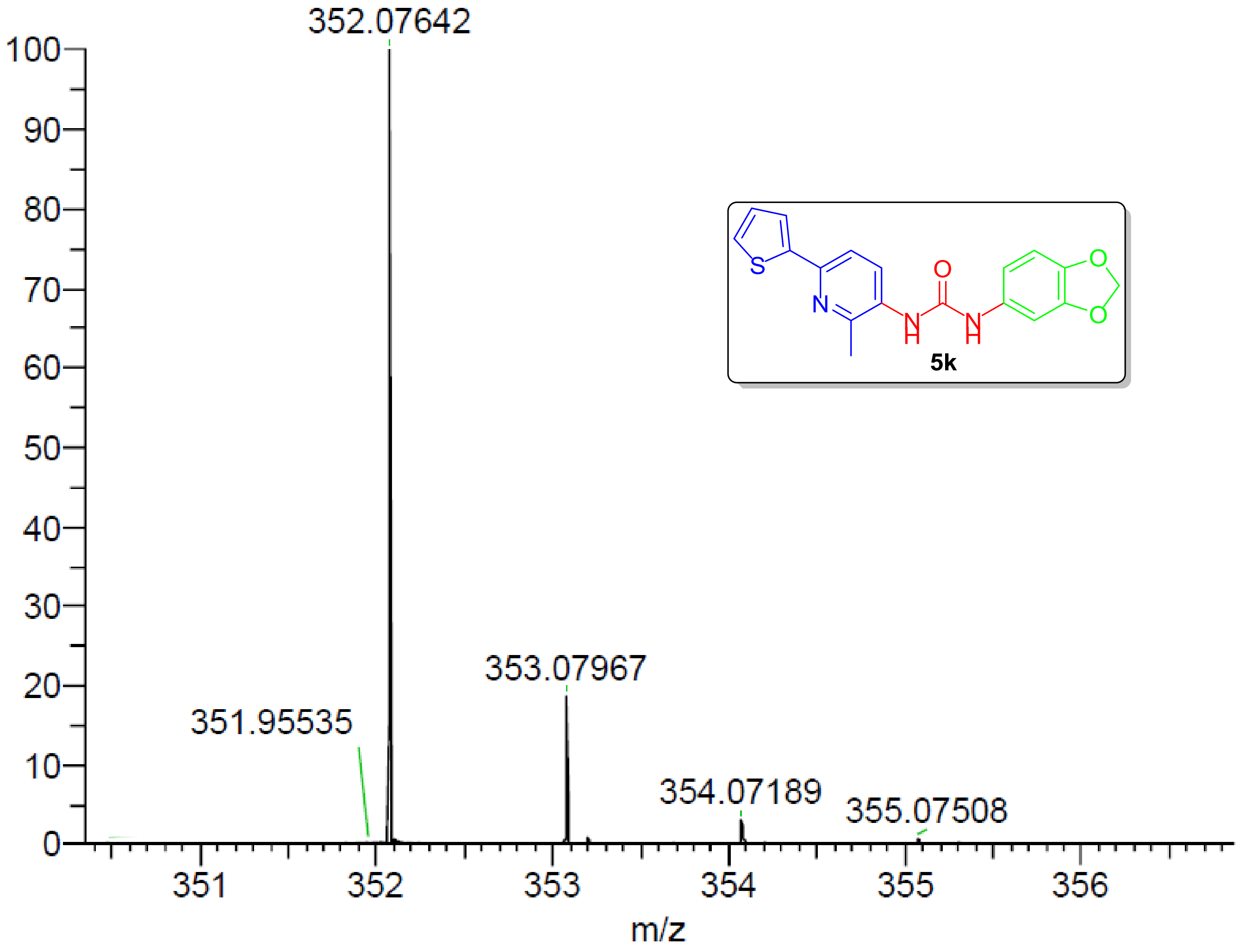

NL: 2.11E6
ESI64425 #14-29 RT: 0.16-0.32 AV: 8 NL: 2.11E6
T: FTMS {1,2} - p ESI Full ms [80.00-1600.00]

Measured Spectrum
